# *In Silico* Toxicology Data Resources to Support Read-Across and (Q)SAR

**DOI:** 10.3389/fphar.2019.00561

**Published:** 2019-06-11

**Authors:** Gopal Pawar, Judith C. Madden, David Ebbrell, James W. Firman, Mark T. D. Cronin

**Affiliations:** School of Pharmacy and Biomolecular Sciences, Liverpool John Moores University, Liverpool, United Kingdom

**Keywords:** databases, *in silico*, chemicals, drugs, safety assessment

## Abstract

A plethora of databases exist online that can assist in *in silico* chemical or drug safety assessment. However, a systematic review and grouping of databases, based on purpose and information content, consolidated in a single source, has been lacking. To resolve this issue, this review provides a comprehensive listing of the key *in silico* data resources relevant to: chemical identity and properties, drug action, toxicology (including nano-material toxicity), exposure, omics, pathways, Absorption, Distribution, Metabolism and Elimination (ADME) properties, clinical trials, pharmacovigilance, patents-related databases, biological (genes, enzymes, proteins, other macromolecules etc.) databases, protein-protein interactions (PPIs), environmental exposure related, and finally databases relating to animal alternatives in support of 3Rs policies. More than nine hundred databases were identified and reviewed against criteria relating to accessibility, data coverage, interoperability or application programming interface (API), appropriate identifiers, types of *in vitro, in vivo*,-clinical or other data recorded and suitability for modelling, read-across, or similarity searching. This review also specifically addresses the need for solutions for mapping and integration of databases into a common platform for better translatability of preclinical data to clinical data.

**GRAPHICAL ABSTRACT:**
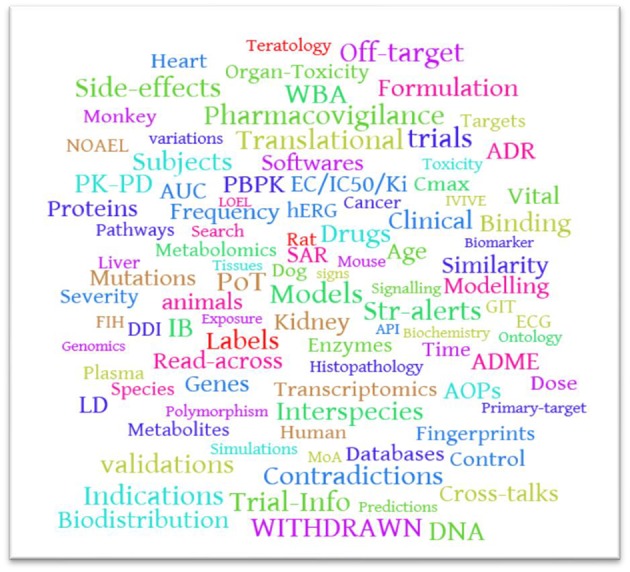
Word cloud of key words in the databases reviewed.

## Introduction

Chemical risk assessment refers to the quantification of any potential adverse effects to humans or environmental species related to exposure to chemicals, drugs, pesticides, consumer products, or any other substances. Traditionally, the assessment of chemicals, including pharmaceuticals, relied on data from animal testing; however, there are many motivations to move to a society free of such testing. In part, the new paradigm for safety assessment embraces the ethos of twenty-first century Toxicology whereby every effort is made to maximise the information that may be obtained without animal testing (Embry et al., 2014). This information may include existing knowledge on the chemical in question, or similar chemicals (the process of read-across), as well as *in vitro* and high throughput determination relating to mechanisms of action and effects at the cellular or organ level (Cronin et al., 2009; Kongsbak et al., 2014). Existing and experimental data are also supplemented by predictions, which may relate to toxicity, mechanisms or exposure that are collectively termed “*in silico*.” There is no formal definition for the process or practice of *in silico* chemical safety assessment; however, it needs to encompass existing knowledge and outputs from predictions of both hazard and exposure as a means of making a decision. There is also increasing interest in making this type of information gathering and assessment more translational, to gain knowledge from all sources to understand the effects on humans—patients in the case of pharmaceuticals—and how that can be translated to mechanisms and assays etc.

There are various types of data that may be considered in modern *in silico* chemical safety assessment. Historical, or legacy, data from toxicological testing provide one of the most important sources of information for modelling and read-across. In theory, data should be available for all endpoints that have been tested across a variety of guideline and non-standard approaches. Such data may be either available openly or be confidential business information and may encompass toxicological and physico-chemical information. These data have been the cornerstone of *in silico* modelling in the past and remain essential for performing safety assessment of existing chemicals. At the other end of the spectrum are upcoming resources that capture mechanistic understanding of chemicals. Such understanding has, in part at least, been facilitated by the so-called “New Approach Methodologies” (NAMs) including *in vitro* High-Throughput Screening (HTS) methods (bioactivity or toxicity profiling bioassays) and omics data generated by more specific genome sequencing, transcriptomics, proteomics, and metabolomics studies (Hartung et al., 2017). These, and other large data repositories, such as clinical effects and adverse drug reactions are routinely referred to as being big data. The term “big data” implies a huge volume of data collected from multiple resources and characterised by their complexity and heterogenous nature. Computational tools often manage big data or algorithms that help to capture, store, search, and analyse the data more rapidly.

Capturing a chemical's physico-chemical properties, bioactivity, and safety profiles or toxicity within databases has become a necessary part of research across many industrial sectors including pharmaceuticals, personal care products, petro-chemicals, and biocides. As a result, *in silico* resources have been reviewed and assessed previously by many researchers, as indicated in Table 1, which identifies 48 of these recent reviews. For example, Young (2002) reviewed web-based resources at the US National Library of Medicine (NLM) including MEDLINE®, PUBMED®, Gateway, Entrez, and TOXNET. As systems biology emerged many gene expression repositories and software were also developed (Anderle et al., 2003; Judson, 2010; Benigni et al., 2013; Fostel et al., 2014). Efforts were not limited to only gene or protein expression databases, but also included organ specific toxicity databases. The review by Fotis et al. (2018) discussed databases relating to genomics, proteomics, metabolomics, multiomics whilst the review by Papadopoulos et al. (2016) focused on such databases specifically relating to the kidney. In relation to other major organs, liver, and heart-related toxicity databases have been discussed by Luo et al. (2017) and Sato et al. (2018), respectively. These diverse types of databases have been further expanded or designed in such a way as to enable interaction with other public resources so improving accessibility for end users. Many resources have emerged that try to link or integrate the chemistry-based databases with bioactivity, pathways of toxicity, ADME, and omics data sets. The chemistry-based databases on small molecules or new compounds were discussed in detail in a number of reviews (Jonsdottir et al., 2005; Williams, 2008; Hersey et al., 2015). Some of the databases that allow for mining of the chemical information (such as 2D, 3D structures, physico-chemical properties etc.) are ChEMBL, ChEBI, PubChem, DrugBank, ZINC, etc. In drug discovery, the number of databases for target identification or prediction of activity, has grown tremendously (Oprea and Tropsha, 2006; Loging et al., 2011; Chen and Butte, 2016; Chen et al., 2016; Katsila et al., 2016; Cha et al., 2018). Other databases containing information on proteins associated with drug therapeutic effects, adverse drug reactions, and ADME properties has facilitated systematic curation and analysis of complex ligand-target data (Ji et al., 2003a). Some of the ADME, potential drug-drug interaction (DDI) information and pharmacogenomics-related databases have been cited in a number of review articles (Ekins et al., 2005; Bauer-Mehren et al., 2009; Ekins and Williams, 2010; Sim et al., 2011; Peach et al., 2012; Wishart, 2014; Ayvaz et al., 2015; Zhang et al., 2015; Przybylak et al., 2018). Fouretier et al. (2016) identified human drug safety data resources, or pharmacovigilance databases, specific to every country or subcontinent. The European Union's Innovative Medicines Initiative 2 Joint Undertaking (IMI 2) “Enhancing TRANslational SAFEty Assessment through Integrative Knowledge Management (eTRANSAFE)” project is developing an integrative data infrastructure to combine and utilise data resources, hence has stimulated the work described in this paper. The aim of eTRANSAFE is to drastically improve the feasibility and reliability of translational safety assessment during the drug development process using both publicly available resources in addition to data provided by its partners to facilitate acceptance by stakeholders, including regulatory agencies and international organisations.

**Table 1:** Previous review articles for identification of databases relevant to chemistry and toxicology.

**Reference**	**Categories covered**	**No. of DBs covered**	**Remarks**
Alexander-Dann et al., 2018	Gene expression	12	Microarray software, database management systems
Ayvaz et al., 2015	Potential DDI information resources	14	Clinical, natural language corpora, pharmacovigilance data sources
Benigni et al., 2013	Chemical mutagenicity and carcinogenecity	18	QSAR, Cluster of toxicity databases, risk assessment
Bianco et al., 2013	Genetic disease research databases	18	Sample sequence, gene expression and post-transcriptional regulation
Bower et al., 2017	Toxicity databases		Toxicity data resources and format (ToxML) discussed
Cha et al., 2018	Drug repurposing databases	29	Drugs and disease (omics, genomics, transcriptomics, proteomics, epigenetic) databases, omics tools also available
Chen et al., 2016	Drug-target interaction databases	15	Webserver databases and computational models included
Cheng et al., 2017	Drug Target interaction databases	28	3D structure, binding affinities Db, screening programs and data repositories, Curated drug-target interactions
Cronin, 2005	Toxicology databases	26	Sources of chemical structures also described
Cronin, 2010	Toxicology databases	33	Data for QSAR modelling purposes
Ekins and Williams, 2010	ADME/Tox databases	13	Targeted data types required for ADME/Tox and PK databases
Ekins et al., 2005	Systems biology and ADMET	33	HT techniques, systems biology modelling and ADMET modelling included
Ekins et al., 2011	Tuberculosis (TB) databases	13	Computational databases, pathways, cheminformatics tools for TB
Fostel et al., 2014	Toxicogenomics	14	Relevant Databases and Consortia Supporting Systems Toxicology Research
Fouretier et al., 2016	Pharmacovigilance (PV)	11	North American PV databases not covered
Fotis et al., 2018	Omic repositories	48	Omics and pathways, tools provided
González-Medina et al., 2017	Chemical biology databases	11	Online servers and tools for mining chemical and target spaces
Hersey et al., 2015	Chemical databases	10	Bioactivity, Patents, drugs and target, available compound and other
Ji et al., 2003b	Proteins associated with drug therapeutic effects, ADR and ADME	44	Targets related databases and their websites
Jonsdottir et al., 2005	Prediction methods, cheminformatics DBs	23	General, screening compounds, medicinal agents, physicochemical and ADMET properties
Judson, 2010	Toxicology databases	15	*in vitro, in vivo* toxicity and ontology-based databases
Kiyosawa et al., 2006	Microarray databases	7	Large scale toxicogenomics databases
Koutsoukas et al., 2011	Bioactivity and target predictions	20	Bioactivity and target-based databases, WS for target prediction of small molecules
Katsila et al., 2016	Drug target identification databases	19	Human metabolome, pathway analysis, chemogenomic data, drug-target, protein, disease specific target DB, pharmacogenomic, toxicogenomic, target-toxin, protein expression, therapeutic target
Loging et al., 2011	Drug repurposing	11	Public resources
Luo et al., 2017	DILI databases	11	Liver specific injury and broader drug databases
Madden, 2013	Toxicity, reactivity, chemical property and structural data	30	Assessment of quality data *provided* (Klimisch score criteria)
Madden et al., 2019	PBPK and ADME Resources	~100	Resources to predict external exposure, physico-chemical properties, ADME properties, physiological/anatomical parameters and model structures for specific organs, PBPK modelling softwares, similar chemicals
Nicola et al., 2012	Medicinal chemistry databases	12	Databases of binding and bioactivity data for small molecules
Opassi et al., 2018	Chemical-Biology databases	28	Virtually accessible chemical spaces, biology databases
Oprea and Tropsha, 2006	Target, chemical and bioactivity	24	Integration of the databases
Papadopoulos et al., 2016	Omics databases on kidney disease	18	General omics and kidney specific databases
Peach et al., 2012	Metabolism related content	11	Software for metabolism predictions
Polen et al., 2008	Online drug databases	14	Drug databases for infectious disease therapies
Rana et al., 2012	Receptor and binding databases	26	Websites for computational, GPCR specific and nuclear receptors
Rigden et al., 2016	Molecular biology databases	157	Nucleic acids, genetic basis of cancer, patented drugs, their side effects, withdrawn drugs, and potential drug targets
Sato et al., 2018	hERG inhibitors, cardiotoxicity	4	hERG inhibition by small molecules
Sim et al., 2011	Pharmacogenetics	7	Pharmacogenomics, CYP, NAT, Transporters, UGT, ADME Dbs
Smalter Hall et al., 2013	Chemical and biological databases	20	Protein interaction, pathways, drug discovery, mathematical models databases, data formats for proteomics and genomics and cheminformatics provided.
Toropov et al., 2014	Drug toxicity databases	27	Software for QSAR analysis of toxic endpoints also given
Williams, 2008	Chemical property databases	15	Publicly available databases
Wishart, 2014	Drug metabolism research	13	Online databases and prediction software for drug metabolism
Wooden et al., 2017	Big data analysis resources	18	Big data for gastro intestinal and liver diseases
Young, 2002	Genetic toxicology web resources	13	EPA, FDA, US NLM toxicity databases discussed
Zou et al., 2015	Biological databases for human research	>100	DNA, RNA, Proteins, expressions, pathways, disease, ontology and literature-based databases listed
Zhang et al., 2015	Pharmacogenomics	8	Web resources

In spite of many previous reviews of data sources for specific types of data (chemistry based, toxicology, omics, ADME etc.), or those predominantly focussed on a specific type of data, no review exists covering all data resources that may be required for twenty-first century toxicology and translational sciences in drug discovery. Moreover, many reviews have failed to address the importance of the full identification, mapping and integration of chemical and biological spaces. Thus, the aim of this study was to provide a comprehensive and consolidated list of *in silico* data resources for chemical safety assessment. This review aimed to encompass all data resources including those based on chemistry, pharmacological space, genomics, and adverse events, as well as those relevant to toxicology and human effects or clinical safety studies. The databases were assessed in groups based on their purpose and information content; data relating to each resource was recorded and summarised. It is intended that this review will provide a valuable starting point for researchers wishing to gain knowledge about a chemical substance and its exposure/effect in preclinical and clinical studies.

## Methods

Initially, the different types of databases to be reviewed were established. The categories chosen for investigation were chemistry-based databases containing information on: toxicology (preclinical studies for chemicals and drugs), genes or enzymes, pathways or AOP-related, omics, protein-protein interactions, ADME, drug discovery, clinical trials, pharmacovigilance, patent-based, environmental chemical exposure, nanomaterial toxicity and animal alternatives, or 3Rs related databases. These categories were utilised to facilitate the searching and grouping/clustering of databases. An iterative process was followed to identify the multiple independent, disparate databases by searching for specific category-based databases in published review papers (Table 1), regulatory-based websites (US FDA, EPA etc.), chemical/pharmaceutical company websites and some of the specific resources on databases such as Toxnet^1^, Pathguide^2^, Fair sharing^3^, VLS3D^4^, Wikipedia^5^, Oxford Journal's biological databases^6^, and AltTox.org^7^ etc.

The criteria by which the databases were assessed were established and are summarised in Table 2; the criteria stipulate both essential and desirable features. Each database was screened using these criteria and only those meeting minimum requirements are included within this review. The most important criterion on which to select a database was its ease of accessibility. The databases could be considered as “open” (free to accesss or use, right to share and re-use) or “partially open” (where only partial metadata are available to access or download or not intended for commercial use). Some of the databases for which the URL links are retired were removed from the list. The availability of information on the type of Application Programming Interface (API) or the programming codes used to develop the databases was also considered as a criterion in selecting the databases. Other essential criteria include- appropriate chemical identifiers (such as SMILES, InChIs etc.), readily converted ontologies and the relevance of the endpoint(s). The desirable attributes included access to the metadata, study protocols, information on data quality assessment, ease of navigation, and other database statistics relating to the frequency of updates and number of compounds or drugs reported. Additional criteria related to the nature of the information provided or the potential use of the database and covers the type of data recorded (*in vitro/ in vivo*/ biomarkers/ omics/ clinical data etc.). The type of data incorporated in the database was limited not only to experimental data (*in vitro, in vivo*) but also included predicted data, quantitative structure-activity relationship (QSAR) models, similarity searching for chemicals or genes and read-across methods.

**Table 2:** Considerations for characterising the databases.

**ESSENTIALS**
Accessibility (open access; registration; license required)
Interoperability (linkage via API or importable)
Acceptable ontology and units (or readily converted)
Appropriate identifiers used (e.g., InChI)
Relevance of endpoint (s) project: physico-chemical properties; ADME (including metabolite data); pharmacological activity; toxicity; clinical trial data; adverse events reports
**DESIRABLES**
Access to metadata
Information provided on study protocols/statistics
Data quality assessment and accuracy of information
Ease of use/navigation
Appropriate classification codes (e.g., therapeutic group classification)
Currency of information (historical; frequency of updates); size of resource (amount of data / level of detail)
**NATURE OF INFORMATION/POTENTIAL USE**
Type of data recorded (*in vitro/in vivo*/biomarker/omics/targets) etc.
Relevance to overall aim of any project (e.g., extrapolation from preclinical to clinical)
Experimental vs. predicted values
Insights into mechanisms of action/elicitation of molecular initiating event
Suitability for modelling, read-across, or similarity searching

The list of more than 900 databases is provided in Table 3 and other detailed information of each database is compiled in an Excel spreadsheet (Supplementary Information). The information included: the name of the database; the owner of the database; any licensing requirements or restrictions on use; the URL or other information on database location; access rights (e.g., registration requirement, free access, potential to download all data etc.); endpoints covered; number and type of compounds included; granularity of data (e.g., test results, dose-response data, experimental conditions etc.) and details on data quality assurance (e.g., details of curation and assignment of quality score such as Klimisch scores) where applicable. Klimisch scores (Klimisch et al., 1997) are assigned to toxicological or physico-chemical data to assess their adequacy, relevance and reliability. The scores are as follows: 1 = reliable without restrictions; 2 = reliable with restrictions; 3 = not reliable 4 = not assignable. These scores are based on criteria such as whether the study was conducted under Good Laboratory Practice (GLP) and if key information on test substances, experimental conditions and statistical evaluation was stated.

**Table 3:** Complete listing of all databases identified in this study grouped according to content (URL links available on-line).

**Chemistry (80)**			**Toxicological (57)**			**ADME (38)**
AuroraFineChem	DOCK Blaster		OCHEM	ACToR	Lhasa Carcinogenecity Db	ADME-AP
Biovia ACD	Danish QSAR Db		OSDDChem	Acute Tox	LIVERTOX	ADME Db
Biovia SCD	Elsevier Reaxys		Organic syntheses	Akos Toxicity	LTKB	ADMET SAR
BioByte	eChemPortal		PubChem	Biovia Toxicity	MDL-Toxicity / met	ADMET Lab
CAS SciFinder	e-Drug3D		PPD	CPDB	NCI-60 (DTP)	ADMENET
CCID	eMolecules		PCDDB	COSMOS	NCTRIcdb	Akos Metabolites
Cfam	eQuilibrator		Probes & drugs portal	CTD	Open TG-GATEs	AMED Cardiotoxicity
ChemDB	FDB-17		Probe Miner	CEBS	OEHHA Chemical	Bioprint
CCDS	FilTer BaSe		R & D Chemicals	ChemTunes	pCEC	BBB
Chemistry Dashboard	FDA UNII		SDBS	Coptis Tox	PROTOX	CYP DI table
ChEMBL	GDB Db		Spresi	CCRIS	PAFA	CYP P450 Inhibitors
ChemAgora	GOSTAR MedChem		Sigma-Aldrich	CREST Db	RTECS	DIDB
ChemSpider	HDAC Inhibitors Base		Symmetry @Otterbein	DSSTox	Repdose	EDETOX Db
Chemfinder	IUPAC-NIST Solubility		SPIM	diXa	Super Toxic	e-PK gene
ChEBI	IUCLID		SIDS	DevTox	SAR Genetox Db	ECVAM KinParDB
Common chemistry	JRC QSAR Model Db		Wikipedia	Drug Matrix	SAR Carcinogenicity	FINDbase
Chemspace	LipidBank		WebReactions	DART	Toxline	HIA
Chembiobase	Lipidomics Gateway		ZINC15	eTox	ToxDB	IDAAPM
Chemexper	LookChem			EADB	Toxygates	IIMDB
CCCBDB	Massbank			EDKB	Toxbank	Liceptor
ChEBI	MolPort			ETOXNET	Tox 21	Microsomal Stability
CERES	METLIN			EASIS	Toxcast	METRABASE
Chem synthesis	mzCloud			EDCs DataBank	ToxRefDB	OI-DDI
COD	MERCK INDEX Online			ECVAM Geno	T3DB	pKa DB
CheLIST	MMsINC			FeDTex	TerraTox	PACT-F
ChemIDplus	NCI-OPEN Db			Gene-Tox	Vitic Nexus	PK/DB
Chemistry Guide	NICEATM Ref Chem Lists			HESS		Pharmapendium
ChemACX	NMRShiftDB			ISSTOX		PDSP Ki
ChemSink	NIST Chemical Kinetics			ITER		Tox-database.net
ChemSub Online	NIST Chemistry Web Book			JECDB		TransportDB
Common Compound Library	NIST, Atomic Spectra			Leadscope		TCDB
						TR MetaDrug
**ADME**		**Drug discovery (157)**				**Clinical trials/ PV (116)**
TP-Search	Allosteric database	Cyclonet	Ensembl Protists	Metabase	PoSSuM	ATC-DDD
TTD	ASDCD	CPRG	ExCAPE-DB	Metacore	PathogenBox	AACT DB
TRANSFORMER	AffinDB	ChEMBL-NTD	e-Drug3D	MOSAIC	RepurposeDB	ALFRED
UCSF ph'genetics	APD	CancerDRD	EMBASE	MEDock	Rx Nav	AFND
UCSF-FDA TransPortal	AutoBind	Cell Image Library	ELDD	MICAD	Sc-PDB	AutDB
WOMBAT-PK	ARDB	CCGD	FDA NDC	MTB	SuperDRUG 2.0	BfArM (UAW-Datenbank)
XMetDb	Abiofilm	Chemical Probes	FDA Orange Book	mSignatureDB	Super Target	BmDR
	Autism Chromosome Rearrangement	CARD	FaCD Online	mutLBSgeneDB	SM2miR	Bioportal
	ADHDgene	cBioPortal	Flow Repository	MK4MDD	SuperPred/target/toxic	BRCA Exchange
	Allergome	CCDB	GenomeCRISPR	MethyCancer	SuperPain	BioLINCC
	AutismKB	CS-DEGs	GOLD	NPASS	Swiss Bioisotere	BioProject
	Biovia MDDR	DART	Gene DB	NCATS	SIMAP	Colorectal Cancer Atlas
	Biovia CMC	DPD	GDKD	NLDB	Swiss Dock	CKB
	Binding DB	Drugs@FDA	GLIDA	NCCN	Swiss Sidechain	CancerPPD
	Binding MOAD	DT-Web	GDSC	NCGC	SFARI Gene	Clinical Codes
	BioByte	DTOME	HMDC	NeuroMorpho.Org	TDR Targets	CVRG
	BioLiP	DRH	HIV-1 Human Int	NIF	TBDTBD	C-Path
	Brenda	DTC	HIV Drug Resistance	NPACT	THPdb	CPRD
	Biomodels	DrugEBIlity	Human TFDB	NCG6.0	TCM-ID	CPIC
	BioMart	DrugMiner	IntSide	Neuron DB	TPDB	ClinVar
	BCNTB Bioinformatics	DCDB	ICGC	OBO Foundry	TTD	ClinicalTrials.gov
	BARD	Drug Bank	Integrity	Oral Cancer Gene Db	TAG	CDSCO
	BIG Data Centre	DSigDB	IQ Consortium	Open Target	TCMID	COSMIC
	BDGene	DrugCentral	Influenza Research	OpenPHACTS	TADB 2.0	CTRP
	CLiBE	D3R	KDBI	Orphanet	TissGDB	ChemDB HIV
	CCD vault	DBAASP	KinMutBase	PICKLES	VKCDB	DSUR
	CTD^2^	Drug2gene	KLIFS	PhID	VIPR	Daily Med
	CancerResource	DGIdb	Kinase SARfari	PLDB	WHO Drug Info	DISQOVER
	canSAR	DNASU	LigDig	PHAROS	TADB 2.0	DAAB
	CARLSBAD	DriverDBv2	LiverAtlas	PharmGKB		Decipher
	CREDO	Disease Meth	MetaADEDB	PROMISCUOUS		Drug Information Portal
	CAMPR3	EuPathDB	Malaria Data	PDBbind-CN DB		
**Biological databases (268)**						
Drug Trials Snapshots	MedLine	SPC/PIL	AbMiner		Candidate Cancer Gene	Eye Gene
Drug Safety Labeling Changes	Micromedex	STRIDE (STARR)	AntiJen		CarpeDB	EuMMCR
Drug consumption Db	Medsafe (SMARS)	Safeguard -DSRU	AlzhCPI		CRISPRlnc	Ebola
Disease Ontology	MedWatch	Sundhedsstryrelsen	Allen BrainMapAtlas		CirGRDB	ExplorEnz
Drug Product Db	MeSH	STEP	Antibody Registry		CCDS	ERGR
ENCePP	NDF-RT	SUSARs	ABCdb		DisProt	EPDnew
EPOCRATES	NAPDI	T R ‘s Cortellis	AHTPDB		DDBJ	Fraggle
EudraVigilance	ORDO	TGA (DAEN)	ADPriboDB		dbPTM	Fusion GDB
EAHD -CF-DB	Ontobee	TCIA	AntigenDB		Directory of CYP P450	GenBank/WGS
EudraCT	Open FDA	Trialtrove	Aaindex		dbSNP	GlycoEpitope
EU CTR	Opportu Inf and TB Ther D	TERIS	AVPdb		dbGaP	GLASS
EORTC Clinical Trials Db	OSB	UBERON	Addgene		dbVar	GEO
EFO	Pharmacy One Resource	USP-NF	Alliance of Genome Resources		dbNP	GPCRdb
First Databank	PSUR	UMLS	AlloMAPS		DBTSS	Genome 3D
FDA's IND/NDA/ANDA	Protect ADR DB	VarCards	Array Map		DIDA	GTEx portal
FAERS	PMDA	VAERS	AH-DB		DDMGD	GENT
GUDID	PBRER	Vigibase	ACLAME		dbDEMC	GO
GePaRD	PANDRUGS	WHO ICTRP	ADHDgene		DEPOD	Gencode
GHR	Physio Para for Older Adults	WITHDRAWN	bioDBnet		D2P2	GlyTouCan
Gold Standard Drug DB	PEPID	Yellow card	BUSCO		ExoCarta	GtRNAdb
HC-SC (MedEffect)	PharmaVar		Brain Transcriptome		ENCODE	Genome properties
HPO	Pharmacopoeias		Bio Wiki		ExAC	GENATLAS
HEROD	PILLBOX		Broad Bioimage		Enzyme Portal	GermOnline
ICD-10	PDBSE		BioMuta		EMDB	GPMdb
iSAEC	PhysioBank		BDB		Eidogen-Sertanty	GlycoNAVI
IB	PPMI		CPDB		ECO	GeneSeeker
IDA	PDX-Finder		CASBAH		Ensembl	GenomeRNAi
JH APX Guide	PedAM		CAMEO		ENZYME	HAMAP
LOINC	RxNorm		CanEvolve		EGA	HGNC
Lareb	SIDER 4.1		CHOPIN		Enzyme Portal	Human Protein Atlas
MedDRA	SNOMED-CT		CanGem		ENA	HPRD
Medicines Complete	Swiss Var		CATH-Plus		EMPIAR	Human Genome Project
HERvD	Kinweb	MINAS	PDBTM		RMDB	tRFdb
HORDE	KIDFamMap	MIPModDB	PsychEncode		RAID	THPdb
HAGR	LABOME	Modomics	Pro kinase resource		SAGD	The Antibody registry
HEMD	LGICdb	Metal PDB	ProtChemSI		Stanford Tissue Microarray	Telomerase
HPM	Lipidomics Gateway	NRR	PHI		SCDE	The MaxQuant
HIV Molecular Immu DB	LncRNADisease v2.0	NPD	Peptides Guide		Super Hapten	UbiProt
HomloGene	LOCATE	NextProt	PDB		SysteMHC Atlas	UniProtKB
hPSCreg	MGC	NURSA	PRIDE		SNPeffect	UniProbe DB
HGVD	MITOMAP	NC-IUBMB	PROXiMATE		Si Records	ValidatorDB
Histome	miRWalk	NPIDB	PHOSIDA		Superfamily	ViralZone
HEDD	MGnify	NIH 3D Print Exchange	PSCDB		Swiss Lipids	VariO
HGVS	MHCBN	NODE	Proteome Isoelectric Point		SSBD	VDJdb
H-InvDB	MitoProteome	Nextprot	PED		SBCDDB	WebTB.org
iPTMnet	MEROPS	Noncode	Plasmid		starBase	Wnt Db
IEDB	MultitaskProtDB	NATsDB	Probe		SelenoDB	1000,000 Genome Project
IUPHAR	MatrixDB	O-GlycBase	Platinum		SBKB	3did
IMGT	MPSTRUC	OrthoDB	PeroxisomeDB		SWISS-Model	5S RNA
InterPro	MiRBase	Over gene Db	PDBsum		Syn Sys Net	
iProClass	MRMAssayDB	ORDB	Pfam		SynLethDB	
ImmPort	Metagene	Onco Db HCC	Polbase		Sc-PDB	
InSiGHT variant Db	MitoMiner	Organelle DB	PolymiRTS		STRENDA	
IntEnz	Morphinome	Organelle Genome	ProtoNet		SDAP	
IPD	MeDReaders	Open SNP	PrimerBank		SYFPEITHI	
IMGT/GENE-DB	MSDD	OGEE	ProtCID		STRING	
IMGT/mAb-DB	Meth HC	PANTHER	PPT-DB		SM2miR	
IMOTA	miRandola	PRINTS	Rhea		tRNAdb	
Interferome	MetalPDB	PSP	RoadMapepigenomics		TSGene	
IDR	MicrobiomeDB	PRO	RBPDB		topPTM	
JGI Genome portal	Mitocheck	Phospho-ELM	RNAcentral		Tp53	
JGA	Membranome	Phospho 3D	RegPhos		TCDB	
Kinase.com	MetaBase	Plasma Proteome	REPAIRtoire		TubercuList	
**Protein-protein interactions (54)**		**Omics (60)**			**Pathways (38)**	
APID	MIPS	Angiogenes	Incardiome Kb		AOP-KB	TIGER
BioGRID	MENTHA	Array Express	IPD		Aging Chart	Transpath
BCL2DB	MATADOR	Array Track	KUPKB		BioCyc	TriForC
CancerNet	MINT	BiGG	LOMA		Biocarta	TCSBN
ComPPI	ORTI	BioSample	Metabolomics workbench		DIMEdb	UCSD-Nature Signaling Gateway
CAZY	PSMDB	Biostudies	MetaCore		Effectopedia	Wiki Pathways
Complex Portal	PPI AD 2.0	BML-NMR	MutAIT		Gene Network	XTalkDB
CORUM	PiSITE	BMRDB	MitoProteome		HumanCyc	
Differential Net	ProtinDB	BioPlex	MSigDB		iPAVS	
DynaSIN	ProtChemSI	Biosystems	MobiDB		KEGG	
DIP	PINT	BioXpress	MBROLE 2.0		MetaCyc	
DOMMINO	PIMADb	C-MAP	Nephroseq		MetaboLights	
gpDB	PSILO	ccmGDB	NCI-60 (DTP)		MetaMapTox	
GWIDD	PIPs	CEGA	OMIM		Molsign	
HINT	Peptide Atlas	Cancer Genomic Hub	Omics DI		MMMP	
HIP	SCOPPI	CKDdb	PACdb		Nrf2ome	
HPRD	SKEMPI	CTRP	PRIDE		PID	
H-InvDB	SNAPPIView	Depmap	PharmacoDB		PDID	
HCSGD	TRIP 2.0	Disnor	READDB		Pathway Commons	
HitPredict	UniHI	DrugSig	RefSeq		PCD	
Innatedb	Wiki-Pi	DGVa	Rfam		Path Card	
IMEx	3did	DRUGSURV	RGED		pathDIP	
INstruct DB	2P2Idb	DisGeNet	SignaLink2		PathwaysWeb	
IRView		DGIdb	Signor		PathArt	
IntAct		Expression Atlas	TB DRM Db		Path Base	
I2D		FiehnLib Db	TCGA		REACTOME	
IIIDB		GMD	TCAG		Mal Card	
iMOTdb		Gene Card	Uni Carb-DB		yAPOPTOSIS	
iRefWeb		HKUPP	UPdb		SMPDB	
KBDOCK		HMA			STITCH	
miRTarBase		HMDB 4.0			TiPs	
**Patents (9)**	**Environmental exposure (30)**	**Animal alternative methods (39)**	**Nanomaterials toxicity (22)**			
DWPI	ATSDR	ALTBIB	OMIA		CBNI	
EPO	ASTDR MRLs	AWIC	RGD		caNanoLab	
Google U.S Patents	CEDI/ADI DB	AnimAlt-ZEBET	SEFREC		DaNa	
JAPIO	CHE TDD	Animal Research.info	TSAR		Good Nano Guide	
PATDPAFULL	EAFUS	Atlases-Pathology Images	3R		JRC NMs Repository	
SCRIPDB	EWAG-BBD	AnimalTFDB	US EPA Physiological parameters-PBPK		NANoREG- eNanoMapper	
SureChemBL	Exposome	Bgee	VLN		NHECD	
USPTO	ECHA Summaries	Cellosaurus	ZFIN		Nano Database	
WIPO	ECODRUG	Cefic LRI AMBIT			Nano Safety DB	
	enviPath	CEFIC LRI CEMAS			Nano techn standards	
	FDA PAFA DBs	CCLE			Nanowerk	
	heatDB	CellLineNavigator			Nanodic.com	
	Haz-Map	DB-ALM			Nano	
	HSDB	EVA			NM registry	
	HCIS	Eagle-i			NIL	
	Household Products DB	EUROECOTOX Db of bioassays			NanoHub	
	HPVIS	EMMA			NCL	
	IRIS	FCS-free Database			Nanosafety Cluster	
	IARC	Humane endpoints			NECID	
	iPiE DB	Inventory of 3Rs Kno Sources			Nano data	
	LINCS	IMPC			Stat Nano	
	LactMed	ICLAC			Smart Nano Tox	
	OECD-QSAR	Interspecies Db				
	PHAROS	IMSR				
	RiskIE	IGRhCellID				
	REM DB	KERIS				
	RITA	LifeMap Discovery®, Cells & Tiss				
	TEDX	Mouse Atlas of Gene Expression				
	TRI	MPD				
	US EPA ECOTOX	Non neoplastic Lesion ATLAS				
		Organ system heterogeneity DB				

## Results

**Figure 1:**
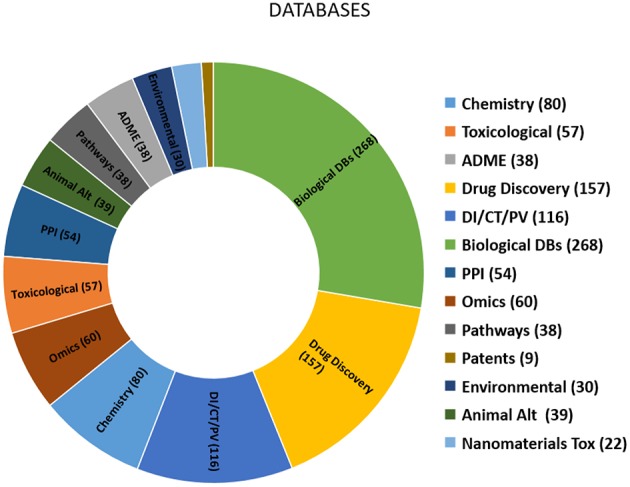
Chart showing the number of databases within each group. DI, Drug Information; CT, Clinical trials; PV, Pharmacovigilance; PPI, Protein-protein interactions; Animal Alt, Animal alternatives.

Based on the criteria established herein for selecting databases, a comprehensive list of more than 900 databases was compiled (Table 3) and consolidated. The types and relative proportion of databases identified are summarised in Figure 1. The key features of the databases within each of the 13 groups identified are summarised below.

- **Chemistry Databases**
  Eighty chemistry databases were identified which relate to resources containing a large library of diverse chemicals or compounds with additional information such as name, molecular formula, structure (2D/3D), key identifiers (CAS Registry Number, IUPAC name, InChI, SMILES), physico-chemical properties, and associations with their bioactivities^8^. Generally, such databases enable users to perform chemical similarity searches based on different fingerprinting methods such as MACCs, Atom pairs, and Topological Torsion Fingerprints etc. However, despite potentially high numbers of compounds, many of these databases are very sparsely populated particularly with regard to high quality toxicity data. Such data resources do provide a useful and usable gateway to multiple databases such as ChEMBL, KEGG, GeneTox, Daily Med etc.
  The applicability, efficiency, and diversity of any database depends on the extent of coverage of chemical space. Within the chemical databases, PubChem (Butkiewicz et al., 2017) includes the highest number of compounds (97 million compounds, 238 million substances, 264 million bioactivities, 3 million patents, and 633 data sources) in comparison to ChemSpider (Pence and Williams, 2010) (68 million chemical structures, 252 data sources), and ChEMBL (Gaulton et al., 2017) (1.8 million compounds and 1.1 million assays). However, the uniqueness of these chemistry-based databases lies in their associations with bioactivities. For example, ChEMBL provides data on bioactive molecules (drug-like properties) and links chemical, bioactivity, and genomic data. ChemSpider provides information on physical properties, biological activities (where available), interactive spectra, the name of chemical suppliers, and other miscellaneous information.
  The chemistry-based databases also possess additional tools such as drawing tools, the capability to search for similar structures (both 2-D and 3-D) using similarity scorings, facilities for structure clustering, identifier exchange services, classification browsers, facilities enabling bulk download etc. For the purposes of virtual screening of compounds, building blocks or scaffolds/fragments, many databases were identified such as ZINC-15 (Sterling and Irwin, 2015), ChemSpace^9^, eMolecules^10^, Generated DataBase (GDB-DB) (Ruddigkeit et al., 2013), Biovia Screening Compounds Directory (SCD)^11^, Probes and Drug portal etc. (Skuta et al., 2017). Zinc-15 covers over 230 million ready-to-dock compounds in 3D-formats and 750 million purchasable compounds for screening purposes. In comparison to Zinc-15, Chemspace covers 100 million, eMolecules 1.5 million and GDB-DB 26.4 million structures for small organic molecules. Biovia SCD contains 9.7 million unique “drug-like” chemicals for HTS and lists over 21 million individual products with prices and supplier ordering information.
  The Probes and Drugs portal is a partially open resource of bioactive compounds (probes, drugs, kinase inhibitors etc.) for commercial screening purposes. It contains 48 compound sets, 46,401 compounds, 34,887 standardised compounds, 18,612 scaffolds, 6,206 targets, 498,201 bioactivities, 2,455 pathways, 4,174 target/pathway classes, 483 structural alerts, and 2,791 matched structural alerts. Other DBs such as ChemDB (Chen et al., 2007) and OCHEM (Sushko et al., 2011, 2012) could be useful for providing a modeling framework to perform QSPR/QSAR studies online. ChemDB contains nearly 5 million small molecules and includes data on predicted or experimentally determined physicochemical properties (3D structure, melting temperature, and solubility). It also includes a chemical fingerprint-based method to search for similar chemicals based on atom-bond connectivity. OCHEM contains 20,56,039 records for 548 properties (physchem or ADME related) and structural alerts for endpoints such as mutagenicity, skin sensitisation, aquatic toxicity, etc.
- **Toxicological Databases**
  Fifty-seven databases were identified pertaining to resources providing information on the effects of drugs or xenobiotics on cells, organs or the whole body^12^. Data are available of many different toxicity endpoints such as: endocrine disruption, mutagenicity, carcinogenicity, skin sensitisation, teratogenicity, organ-specific toxicity etc. Data are either derived from *in vitro* or *in vivo* studies or from *in silico* prediction across multiple species. Of the databases identified, a noteworthy example is the US EPA's Aggregated Computational Toxicology Online Resource (AcToR) (Judson et al., 2008), covering over 500,000 chemicals. It is the warehouse for many EPA's web-based applications such as the Chemistry Dashboard (over 700,000 chemicals and includes chemical structures, experimental and predicted physicochemical, and toxicity data), Toxicity Forecaster (ToxCast) Dashboard (HTS data on over 9,000 chemicals and information on ~1,000 assay endpoints), Endocrine Disruption Screening Program in the twenty-first century Dashboard, Chemical Product Category (CPCat), and exposure databases for personal care products.
  Other significant toxicological data resources include the Gene-Tox database from the National Institutes of Health (NIH) U.S. National Library of Medicine (NLM) which comprises mutagenicity data for more than 3,000 chemicals. The RepDose and FeDTex databases (Bitsch et al., 2008) are useful sources for No Observed (Adverse) Effect Level (NO(A)EL) or Lowest Observed (Adverse) Effect Level (LO(A)EL values from repeated dose studies for reproductive and developmental toxicity endpoints. RepDose consists of data on >400 chemicals investigated in 1,018 studies resulting in 6,002 specific effects. The HESS database (Sakuratani et al., 2013) contains information on 28 day repeat dose toxicity studies for 289 industrial chemicals in rats and includes additional rat metabolism datasets and information on ADME in rats and humans.
  The list of databases also includes toxicogenomic related data sources such as the Comparative Toxicogenomics Database (CTD) (Davis et al., 2011), Open-TG Gates (Igarashi et al., 2015), The Data Infrastructure for Chemical Safety (diXa) (Hendrickx et al., 2015), Toxygates (Nyström-Persson et al., 2013; Natsume-Kitatani et al., 2017) etc. Open-TG-GATES stores gene expression profiles and traditional toxicological data derived from *in vivo* (rat) and *in vitro* (primary rat hepatocytes, primary human hepatocytes) exposure to 170 compounds at multiple dosages and time points. Toxygates is the new interactive version of data from Open-TG-GATES covering 24,011 samples and 170 compounds. The diXa database provides a one stop resource for toxicogenomics studies with cross-links to chemical and molecular medicine databases. diXa contains data from 34 studies involving 469 compounds and recently, all the data have been migrated to the BioStudies (EMBL-EBI) platform (Sarkans et al., 2018). BioStudies contains biological data or models and links them to external resources. At the time of writing it contains 2,552,605 files, 2,824,923 links, four projects, and 1,214,176 studies. CTD is another valuable resource which includes more than 30.5 million toxicogenomic connections relating chemicals/drugs, genes/proteins, diseases, taxa, Gene Ontology (GO) annotations, pathways, and gene interaction modules.
  ChemTunes & ToxGPS consists of *in vitro* and *in vivo* toxicity endpoint specific alerting chemotypes; mechanism of action (MOA) based QSAR models, weight of evidence (WoE) outcomes, and ToxGPS datasets. Other organ specific toxicity databases inlcude: AMED cardiotoxicity (Sato et al., 2018), LiverTox (Hoofnagle et al., 2013), Liver Toxicity Knowledge Base (LTKB) (Chen et al., 2011), the National Center for Toxicological Research liver cancer database (NCTRIcdb) (Beger et al., 2004) etc. The AMED cardiotoxicity database contains data on small molecules that bind to various ion channels and potentially cause cardiotoxic risk. The data on bioactivities for hERG potassium channel were collected from ChEMBL, the NIH Chemical Genomics Center and hERGCentral. They consist of 9,259 hERG inhibitors (IC50 ≤ 10 μM) and 279,718 inactive compounds (IC50 > 10 μM). LiverTox is comprehensive resource on drug induced liver injury caused by prescription and non-prescription drugs, herbals and dietary supplements (1000's of DILI agents). LTKB has been developed by the US FDA's National Center for Toxicological Research to study drug-induced liver injury (DILI). The data are related to DILI mechanisms, drug metabolism, histopathology, therapeutic use, targets, side effects, biomarkers etc. DILIrank consists of 1,036 FDA-approved drugs that are divided into four classes, Most-DILI-concern drug (192 drugs); Less-DILI-concern drug (278 drugs), No-DILI-concern drug (312 drugs), and Ambiguous-DILI-concern drug (254 drugs). NCTRIcdb contains 999 chemicals classified as 273 liver carcinogen, 293 other carcinogen, and 304 as non-carcinogen based on studies of male and female mice and rats.
  The eTOX database was developed by the European eTOX project, which was a consortium of 13 pharmaceuticals, data curators, modellers and software developers funded by the EU Innovative Medicines Initiative (IMI) Joint Undertaking for 7 years (Steger-Hartmann et al., 2009; Cases et al., 2014; Sanz et al., 2017). The database provides access to data on repeated dose toxicity and organ specific toxicity studies and contains models such as like Human outcomes module, Ontobrowser, eTox Lab, and Limtox. It covers data on 1,947 pharmaceuticals out of which 483 labelled as confidential. COSMOS (Cronin et al., 2012) was another EU funded project which aimed to develop *in silico* models for the prediction of human repeated dose toxicity of cosmetic ingredients to optimise safety without the use of animals by using computational models. The tools and approaches includes application of Thresholds of Toxicological Concern (TTC) of cosmetics related substances. The database includes more than 80,000 chemical records with more than 40,000 unique structures, 12,000 toxicity studies across 27 endpoints for more than 1,600 compounds.
- **ADME Databases**
  Thirty-eight ADME databases were identified which captured information on parameters such as area-under-the-plasma concentration-curve (AUC), maximum concentration (C_max_), Time to reach maximum concentration (T_max_), half-life (T_1/2_), volume of distribution (V_d_), clearance (CL) etc^13^. These data were determined from *in vitro* and *in vivo* ADME studies involving different species (mouse, rats, dogs, monkeys) as well as humans in clinical studies. Pharmapendium (https://pharmapendium.com) is one of the most widely used commercial database (Elsevier group) in the pharmaceutical industry. The database contains information on 4,331 drugs indexed and fully searchable for more than 1.53 million PK data, 295,000 metabolising enzyme and transporters data, 1.57 million safety data, 1.69 million efficacy data and 115,000 activity data extracted from FDA/ EMA drug approval documents.
  A number of databases are particularly useful for the retrieval of information on metabolites e.g., XmetDB (Spjuth et al., 2016), Metrabase (Mak et al., 2015), and Akos. XMetDB is an open resource for drugs, xenobiotics and their experimental metabolite data. It contains 162 observations from 21 scientific papers from 14 journals, covering 117 chemical structures and 95 enzymes. Akos Metabolites (Accelrys) is a restricted source containing experimental data from *in vivo* and *in vitro* studies for about 20,000 parent compounds, 100,000 transformations, and 50,000 molecules. It has indexed relevant metabolic paths for structurally related systems.
  Other influential databases for PK properties include the ADME database^14^ which is a proprietary database from Fujitsu Kyushu Systems (Japan). This provides the latest and most comprehensive data on interactions of substances with drug metabolising enzymes and drug transporters that are specific to humans. It contains enzyme kinetic values (K_m_, V_max_, K_i_, K_inactivation_, IC_50_, EC_50_, T_1/2_) obtained from the literature. A recent count shows 35,776 substrates, 28,996 inhibitors, 546 activators as well as 12, 617 data for inducers of CYP450 enzymes. For other enzymes 8,608 substrates, 6,109 inhibitors, 229 activators, and 2,220 inducers were included and in the case of transporters 14,749 substrates, 6,109 inhibitors, 229 activators, and 2,220 inducers were included. The University of Washington's licensed databases such as Drug-Interaction DB (DIDB), e-PK gene, and Organ induced-DDI (OI-DDI) (Hachad et al., 2010, 2011; Yeung et al., 2015) are also very useful. DIDB has the largest manually curated collection of *in vitro* and *in vivo* data related to drug interactions in humans. Additionally, it covers pharmacokinetic profiles of drugs, QT (i.e., the time between the start of the Q wave and the end of the T wave in heart's electrical cycle) prolongation data, including results of Thorough QT (TQT) from recent New Drug Application's (NDAs) clinical trials data. e-PK gene, is based on pharmacogenomics i.e., providing in-depth analysis of the impact of genetic variants of enzymes and transporters on pharmacokinetic responses to drugs and metabolites.
  OI-DDI contains information on pharmacokinetic drug exposure data for 271 compounds from publicly available renal and hepatic impairment studies presented together with the maximum change in drug exposure from drug interaction inhibition studies. Databases on transporters include TP-Search (Ozawa et al., 2004) and UCSF-Trans Portal (Morrissey et al., 2012). TP-Search enables the user to search the membrane transporters-related information by substrate/inhibitor/inducers structure or name, gene expression, functions, drug-drug interactions involving transporters (K_m_/K_i_) etc. It coveres more than 75-membrane transporters across different tissues in mice, rats and humans. The University of California, San Francisco's UCSF-Trans Portal provides information on transporter expression, substrates, inhibitors and potential drug-drug interactions. The localisation of transporters in different organs e.g., blood-brain barrier, kidney, liver, placenta, and small intestine is available with images, notes, references, and expression data where available. UCSF Pharmacogenetics (Kroetz et al., 2009) is a restricted knowledge base providing the information on genetic variants in membrane transporters in ethnically diverse populations. The complete list of Solute Carrier Superfamily (SLC) and the ATP Binding Cassette (ABC) Superfamily are also provided within UCSF. The user can also access qPCR expression and MicroArray data. IDAAPM (Legehar et al., 2016) is a very useful and freely accessible computational resource for modelling (using KNIME workflows) to provide information on ADME properties and known adverse effects of FDA approved drugs taken from FAERS database. It contains information of about 19,226 FDA approval applications for 31,815 products, 2,505 active ingredients, 1,629 molecular structures, 2.5 million adverse effects, and 36,963 experimental drug-target bioactivity data. ADMETlab is a recent addition in this category which is useful to predict 31 ADMET endpoints prediction, systematically evaluate PK properties and druglikeness as well as performing chemical similarity searching using different fingerprint methods (Dong et al., 2018).
- **Drug Discovery Databases**
  One hundred and fifty-seven databases were identified that relate directly to drug discovery including: small molecule screening, combinatorial chemistry, molecular affinity, binding, docking, enzyme interaction, activity, gene expressions, side-effects, disease, pathways, repurposing of drugs etc^15^. Some of the pertinent resources on drug information are DrugBank, DrugCentral, SuperDrug and the FDA's Orange Book. DrugBank (Wishart et al., 2008) is one of the most widely used databases that includes information on drugs targets, enzymes and transporters. The version available at time of writing contains 11,874 drug entries including 2,474 approved small molecule drugs, 1,177 approved biotech (protein/peptide) drugs, 129 nutraceuticals, and over 5,748 experimental drugs. Additionally, 5,131 drug target/enzyme/transporter/carrier sequences are linked to these drug entries. DrugCentral (Ursu et al., 2017) is another drug compendium covering 4,531 active ingredients, 3,807 small molecules, 279 biologics, 445 other compounds, 77,484 FDA drug labels, 34,192 prescription only drug labels, 43,292 OTC drug labels, and 97,271 pharmaceutical formulations with FDA drug labels. The web server for Drug Central also aids in finding the drug gene signature profile similarity without linking to other resources. SuperDrug (Goede et al., 2005) contains 4,605 small molecules, 3,993 Biologicals and 612 other drugs, 4,253 ATC codes, 736,562 3D conformers, 223,860 drug products, 3,006 confirmed biological targets, 1,450 predicted biological targets, and 109,698 side effects.
  A small number of early drug development resources were also identified; Open Target (Koscielny et al., 2017) is useful for systematic identification and prioritisation of targets. At time of writing, it contains 21,149 targets, 2,920,121 associations, and 10,101 diseases. D3R (Gathiaka et al., 2016; Gaieb et al., 2018) is another resource containing manually curated datasets on validation and improvement of methods in computer-aided drug design. A single dataset comprises 25 or more congeneric compounds, 5–10 co-crystal structures, related affinity data and a number of known inactive compounds. Biovia MDDR database was jointly developed by BIOVIA and Clarivate Analytics. It contains 260,000 biologically relevant compounds and well-defined derivatives. E-LEA3D (Douguet, 2010) is a source for FDA's registered molecular structures −1884 approved between 1939 and 2018 with a molecular weight ≤ 2000. The 1,884 different molecular structures includes structures of enantiomers and of active metabolites. This resource is dedicated to pharmacology (molecular structures, PK, Pharmacodynamics (PD) and registration data.
  Databases useful for exploring binding affinity to targets are also included in Table 3. BindingDB (Gilson et al., 2016) is an open resource of measured binding affinities, focusing on the interactions of protein considered to be drug-targets with small, drug like- molecules and it contains 1,454,892 binding data, for 7,082 protein targets, and 652,068 small molecules. AffinDB (Block et al., 2006) is a database of affinity data for structurally resolved protein-ligand complexes from the Protein Data Bank (PDB) and it contains 748 affinity data out of which 474 were covered from PDB. Brenda is a widely used resource for enzymes information including ~3 million data points from 83,000 enzymes, 137,000 literature references, and a total of 206,000 enzyme ligands providing functional and structural data (Placzek et al., 2017).
  GPCR-LIgand DAtabase (GLIDA) (Okuno et al., 2006) is a G protein-coupled receptor (GPCR)-related chemical genomic database that is primarily focused on the correlation of information between GPCRs and their ligands. It contains 3,738 GPCR-related entries (links to Entrez Gene, GPCRDB, UniProt, IUPHAR, KEGG) for 649 ligand entries. For docking purposes Computed Ligand Binding Energy (CLIBE) (Chen et al., 2002) and CREDO (Schreyer and Blundell, 2013) are useful. CLIBE is a database developed by National University of Singapore and useful for the analysis of Drug Binding Competitiveness. It contains 67,184 entries for ligand binding energy, in which there are 5,978 distinctive ligands and 2,258 distinctive receptors. In contrast CREDO stores the interactions between all molecules inside macromolecular complexes from the Protein Data Bank (PDB). These molecules include proteins, nucleic acids, carbohydrates as well as small molecules. CREDO has implemented 13, different interaction types such as hydrogen bonds, halogen bonds, carbonyl interactions, and others. Other resources noted are Target Central Resource Database (TCRD), Pharos (Nguyen et al., 2017), CARLSBAD (Mathias et al., 2013), GPCR-Ligand Association Database (GLASS), and GPCR-EXP (Dai et al., 2016). TCRD and Pharos were both developed by the Illuminating the Druggable Genome (IDG) program which aimed for collecting and organising information about the most common protein targets from four families -GPCRs, kinases, ion channels, and nuclear receptors. TCRD collates many heterogenous gene/protein datasets and Pharos is a multi-modal web interface that represents the data from TCRD. Overall, the database covers 72 million associations between all mammalian genes and their attributes collected from 66 open online major resources. CARLSBAD is an aggregator of many bioactivity databases such as ChEMBL, IUPHARDb, Psychoactive Drug Screening program (PDSP) K_i_, PubChem, and WOMBAT. It provides a single normalised bioactivity value (K_i_, EC_50_ etc.) for chemical-protein target pair. It includes data for 9,32,852 activities, 8,90,323 unique structure-target pairs, 3,739 targets, and 1,301 diseases. The GLASS database is manually curated repository for experimentally-validated GPCR-ligand interactions. It contains 3,056 GPCR entries and 335,271 ligand entries. Whereas, GPCR-EXP provides information on experimental and predicted structures of GPCR. It covers data for 55 GPCRs, 282 structures across 9 species (from Protein Data Bank), and 1,076 predicted structures in human genome. Possum (Ito et al., 2012) is another standalone database for pocket similarity searching for predicted and experimentally-derived ligand binding sites covering 5,513,691 known and putative binding sites obtained from Protein Data Bank.
  Allostery is a process of regulation of biological macromolecule (protein) function induced by binding of a ligand (small molecule) at an allosteric site (i.e., a distinct site other than the active site) in an efficient way to control the metabolic mechanisms or signal-transduction pathways and subsequently increasing the high receptor selectivity and lowering the target-based toxicity. This concept helped to build the Allosteric (Shen et al., 2016) database which has compiled 1,788 allosteric target entries, 77,825 allosteric modulator entries, 82,431 interactions, 1,930 allosteric sites, 56 allosteric pathways, 261 allosteric networks, and 3,350 allosteric related diseases.
  For pain research, there are various databases including SuperPain (Gohlke et al., 2014) and Pain Genes database (PGDB) (Lacroix-Fralish et al., 2007). SuperPain is a database specifically relating to pain-stimulating and pain-relieving compounds, which bind or potentially bind or block to ion channels, e.g., those belonging to the family of Transient Receptor Potential (TRP) channels (TRPV1, TRPM8, TRPA1), human ether-a-go-go related gene (hERG), TREK1, P2X, Acid-sensing ion channels (ASIC) or voltage-gated sodium channels. It contains data on 8,700 ligands (experimentally identified) and 100,000 putative ligands. PGDB provides analysis of the published pain-related phenotypes of mutant mice (over 200 different mutants) and covers 430 genes associated with the pain mechanism.
  Oncology is another domain where large datasets have been produced and compiled in databases such as Cancer Target Discovery and Development (CTD^2^) (Aksoy et al., 2017), CancerResource (Gohlke et al., 2016), canSAR (Tym et al., 2016), CancerDRD (Kumar et al., 2013), Genomics of Drug Sensitivity in Cancer (GDSC) database (Yang et al., 2013) etc. CTD^2^ is very useful platform for translation of high-throughput and high-content genomic and small molecule data for oncology. It contains 10,828 cancer cell-line sensitivity profiling data, 18 oncogenomic screening observations, 156 chemical-genetic interaction mappings, 66 drug-sensitivity screening results, 24 observations based on reverse phase protein arrays. Whereas, CancerResource focuses on cancer related drug-target interactions, expression, and mutation data as well as drug sensitivity data. It covers data on 48,404 compounds, 3,387 cancer-relevant protein targets, 90,744 compound-target interactions, 2,037 cell lines, 19,834 genes, 872,658 mutations, 23,016 genes (expression). CanSAR is another translational platform, which integrates genomic, protein, pharmacological, drug, and chemical data with structural biology, protein networks, and druggability data. CanSAR contains unique data on 20,316 proteins in human, 556,825 in all species, 143,698 3D structures, 400,892 chains, 12,172 cell lines and 1,962,718 unique structures, 2,148 organisms, 6,367,677 data points from 59,618 studies, and 244,099 clinical trials. Whereas, CancerDRD is a database of 148 anticancer drugs and their effectiveness against around 1,000 cancer cell lines. GDSCdb is a partially open resource containing information on drug sensitivity in cancer cells and molecular markers of drug response. It contains data on 75,000 experiments testing response to 138 anticancer drugs across almost 700 cancer cell lines.
  With the advent of high—throughput *in-vitro* technologies to systematically investigate new indications for existing drugs has led to the development of repurposing databases for personalized medicines such as RepurposeDB (Shameer et al., 2018), repoDB (Brown and Patel, 2017), The Drug Repurposing Hub (DRH) (Corsello et al., 2017), Promiscuous (Von Eichborn et al., 2011) etc. RepurposeDB contains information on 253 drugs (74.30%) and protein drugs (25.29%) and 1,125 diseases. RepoDB another repositioning database containing information on 1,519 approved drugs, 386 terminated, 199 withdrawn, and 77 suspended drugs. DRH contains data on 10,147 compound samples, 2,247 protein targets, 6,125 unique compounds, and 663 drug indications. Promiscuous connects entities such as drugs, proteins and side effects as well as mapping the relationships between them using a network visualisation approach. To date, data are available for 10,208,308 proteins, 25,170 drugs and drug like compounds, and 23,702 drug-target interactions.
- **Drug Information, Clinical Trials, and Pharmacovigilance Databases**
  One hundred and sixteen up-to-date resources on drug information (medication content, packaging inserts dosing), drug safety (side-effects), clinical trials information, and pharmacovigilance are listed that are relevant for patients, researchers, pharmacists, or prescribers^16^. Of these DailyMed^17^ is the official provider of Food and Drug Administration (FDA) label information (package inserts) and has information for 98,961 drug listings as submitted to the FDA. Medicines Complete^18^ is another resource for authoritative information to support clinical and drug research decisions; it includes publications such as British National Formulary (BNF), BNF for children, Martindale, Stockley's Drug Interactions, Martindale's Adverse Drug reactions, Drug Administration via Enteral Feeding Tubes, AHFS Drug Information, Clarke's Analysis of Drugs and Poisons, Dale and Appelbe's Pharmacy and Medicines Law, Dietary Supplements, Drugs in Pregnancy and Lactation, Handbook on Injectable Drugs, Herbal Medicines, Injectable Drugs Guide, Kucer's the use of Antibiotics, Pediatric Injectable Drugs, Pharmaceutical Excipients, Stockley's Herbal Medicines Interactions, The Green Guide (rules and guidance for the pharmaceutical distributors) and the Orange Guide (rules and guidance for the pharmaceutical Manufacturers and distributors). It covers 600,000 plus pages of evidence-based drug information, 200 plus countries access MedicinesComplete and it has 3.7 million users. MedlinePlus^19^ is a general public education related resource, developed by The National Library of Medicine (NLM), a part of the US National Institutes of Health. It contains information on diseases, conditions, and wellness issues over 1,000 topics.
  There are a few relevant databases related to pharmacovigilance listed in this review such as Side Effect Resource (SIDER) 4.1 (Kuhn et al., 2016), FDA Adverse Event Reporting System (FAERS) (Fang et al., 2014), Vigibase (Lindquist, 2008), and EudraVigilance (Postigo et al., 2018). SIDER 4.1 contains information (extracted from public documents and package inserts) on marketed medicines and their recorded adverse drug reactions; presently it covers data on 1,430 drugs, 5,868 side effects, 139,756 drug-side effects pairs; 39.9% of drug-side effect pairs have corresponding frequency of effect information. Another adverse event related database is FAERS (Fang et al., 2014) which contains adverse event reports, medication error reports, and product quality complaints resulting in adverse events that were submitted to FDA (from Jan 2004 and presently updated quarterly). It contains 14,160,191 total reports, 8,072,400 serious reports (excluding death) and 1,420,885 death reports. Vigibase (Lindquist, 2008) is a unique World Health Organisation (WHO) global database of individual case safety reports (ICSRs), it is linked to medical and drug classifications, including terminologies such as WHO Adverse Reaction Terminology (ART), Medical Dictionary for Regulatory Activities (MedDRA) (Morley, 2014), WHO International Statistical Classification of Diseases (ICD), the medicinal products dictionary, and WHODrug. It holds over 16 million anonymised reports of suspected adverse effects of medicines suffered by patients. EudraVigilance (Postigo et al., 2018) is the European database of Suspected Adverse Reaction reports by the European Medical Agency (EMA). MedDRA provides a single standardised international medical terminology, which can be used for regulatory communication and evaluation of data pertaining to medicinal products for human use. It supports ICH electronic communication within the ICH's Electronic Common Technical Document (eCTD) and the E2B Individual Case Safety Report. There are five levels to the MedDRA hierarchy, at the most specific level it is “Lowest Level Terms” (LLTs), >70,000 terms (e.g., feeling queasy), “Preferred Term” (e.g., nausea), “High Level term” (e.g., nausea and vomiting symptoms), “High Level group term” (GIT signs and symptoms), and “System Organ Class” (e.g., GIT disorders). ICD-10^20^ contains guidelines for systematic recording, coding, analysis, interpretation, and comparison of mortality-morbidity data collected in different countries. ClinicalTrials.gov (Zarin et al., 2011) is a database of privately and publicly funded clinical studies conducted throughout the world. It is a resource provided by the US NLM and the user can explore 288,732 research studies in all the 50 states and in 205 countries. Information on clinical studies (summary and protocols) are updated by the sponsor or principal investigator of the clinical study. The European Clinical Trials Database^21^ (EudraCT) is developed and maintained by the European Medicines Agency. The European Union Clinical Trials Register covers 33,526 clinical trials with a EudraCT protocol, of which 5,428 are clinical trials conducted with subjects 18 years old. The register also displays information on 18,700 older (12–18 years) pediatric trials.
  Ontology driven databases have also emerged recently, as these can help map other databases, this listing therefore includes resources such as the Medical Subjects Headings (MeSH) (Nelson et al., 2001), National Drug File- Reference Terminology (NDF-RT) (Pathak and Chute, 2010), Unified Medical Language System (UMLS) (Humphreys et al., 1998) and The Systematized Nomenclature of Medicine-Clinical Terms (SNOMED-CT) (Bhattacharyya, 2016). MeSH database provides controlled vocabulary thesaurus for medical subjects and it includes medical terms (headings, subheadings, supplementary concept records, publication types) for indexing articles for PubMed. MeSH contains ~26,000 terms and is updated annually. NDF-RT is developed by the U.S. Department of Veterans Affairs, Veterans Health Administration (VHA). It organises drug list into formal representation. The categories of NDF hierarchical drug classifications are Cellular or Molecular Interactions (MoA), Chemical Ingredients, Clinical Kinetics (PK), Diseases, Manifestations or Physiologic States, Dose Forms, Pharmaceutical Preparations, Physiological Effects, Therapeutic Categories, and VA Drug Interactions. NDF-RT is updated monthly as a part of RxNorm. UMLS is a repository of biomedical vocabularies developed by US NLM. It has three components- The Metathesaurus® of inter-related medical concepts, Semantic networks (high-level categories) and the SPECIALIST Lexicon which “contains syntactic, morphological, and orthographic information for biomedical and common words in the English language”. The UMLS covers 2 milion names for some 900,000 concepts from >60 families of biomedical vocabularies, as well as 12 million relations among these concepts. SNOMED-CT is a database on structured clinical vocabulary for use in an electronic health record. It provides a standardised way to represent clinical phrases captured by the clinician and enables automatic interpretation of these. It is multinational and multilingual, and it contains 340,659 active concepts.
- **Biological Databases**
  There are 268 biological databases. This category of databases refers to the resources containing information on enzymes (kinase, GPCRs, CYPs etc.) antibodies, receptors, tissue specific gene expressions/ regulations, annotated protein-peptide sequences, genetic and metabolic signaling, RNA, lipids, immune-system components etc^22^. Enzyme related information are provided in databases such as Enzyme Portal (Alcántara et al., 2013), GPCRdb (Pándy-Szekeres et al., 2018), International Union of Basic and Clinical Pharmacology- British Pharmacological Society (IUPHAR-BPS) Guide to Pharmacology (Hay et al., 2018), Integrated relational Enzyme database (IntEnz) (Fleischmann et al., 2004), Kinase.com, Kinweb etc. Enzyme Portal is a comprehensive database by EMBL-EBI and it contains information on enzymes, such as small-molecule chemistry, biochemical pathways, and drug compounds. It provides a summary of information from UniProt Knowledgebase, Protein Databank in Europe (PDBe), Rhea (enzyme-catalysed reactions), Reactome (biochemical pathways), IntEnz (enzyme nomenclature information), ChEBI and ChEMBL (small molecule chemistry and bioactivity), MACiE (reaction mechanism), and the Experimental Factor Ontology (EFO). GPCRdb contains data on GPCR structures and large collections of receptor mutants. The database covers data on 15,090 proteins, 418 human proteins, 3,547 species, 270 experimental structures, 184 refined structures, 144,860 ligands, 34,353 mutants, and 12,300 ligand interactions. IUPHAR/BPS Guide to Pharmacology provides data on molecular interactions between target and ligands from selected papers in pharmacology and drug discovery since 2003. It covers 2,880 total number of targets, 9,405 ligands and 1,383 approved drugs with clinical use summary.
  For DNA, RNA, and gene datasets, there are number of databases available such as GenBank (Benson et al., 2013), Gene Expression Ontology (GEO) (Barrett et al., 2013), The Genotype-Tissue Expression (GTEx) portal (Stranger et al., 2017), Hugo gene Nomenclature Committee (HGNC) (Gray et al., 2016), Human Protein Atlas (HPA) (Uhlen et al., 2010), Encyclopedia of DNA element (ENCODE) (The ENCODE Project Consortium, 2011), Ensembl, TP-53 (Leroy et al., 2013), European Nucleotide Archive (ENA) (Silvester et al., 2018), European Genome-phenome Archive (EGA) (Lappalainen et al., 2015) etc. GenBank is the NIH genetic sequence database, an annotated collection of all publicly available DNA sequences. It contains publicly available nucleotide sequences for almost 260,000 formally described species. GEO is a portal for the application ontology for the domain of gene expression. Its metrics indicates 166,254 classes, 157,102 individuals, 12 properties and 50,996 maximum number of children. GTEx database is a great resource to study tissue-specific gene expression and regulation. The 11,688 samples were collected from 53 non-diseased tissue sites and 714 donors. HGNC is the worldwide authority that assigns standardised nomenclature to human genes. It approves both a short-form abbreviation known as a gene symbol and a longer and more descriptive name. HPA database consists of three parts; the Tissue Atlas provides the distribution of proteins across all major tissues and organs in the human body, the Cell Atlas provides the subcellular localisation of proteins in single cells and the Pathology Atlas shows the impact of protein levels for survival of patients with cancer. In the latest version, the Human Protein Atlas contains more than 26,000 antibodies, targeting proteins from almost 17,000 human genes.
  The ENCODE consortium is supported by the National Human Genome Research Institute (NHGRI) and it has systematically mapped regions of transcription, transcription factor association, chromatin structure, and histone modification. The ENCODE portal contains over 13,000 datasets available through the portal from human, mouse, *Drosophila* and *Caenorhabditis elegans* assayed under a variety of different physiological conditions. The Ensembl database provides a bioinformatics framework to organise biology around the sequences of large genomes. Ensembl annotate genes, computes multiple alignments, predicts regulatory function, and collects disease data. TP-53 (tumor protein or cellular tumor antigen p53) is a database related to the structure of the TP53 gene, TP53 isoforms, mutation nomenclature, and the sequence of more than 5,000 tumor samples from 12 cancer types. The current release contains 80,400 tumors, 6,870 different TP53 variants. The ENA (EMBL-EBI) is a comprehensive resource on world's nucleotide sequencing information, raw sequencing data, sequence assembly information and functional annotation. It contains information on 2042.8 millions of nucleotide sequences and 5021.7 billions bases. The EGA contains data on personally identifiable genetic and phenotypic data resulting from biomedical research projects. In the entire EGA there is a total of 1,706 studies (960 cancer, 134 cardiovascular, 39 infectious, 59 inflammatory, 63 neurological, and 362 others).
- **Protein-Protein Interaction Databases**
  To understand the relationships between proteins, protein-protein (PP) interaction studies are required and this has led to the creation of many valuable databases to catalog and annotate these PP interactions. Fifty-four databases are listed in Table 3^23^. Some of the most valuable resources are Agile Protein Interactomes Data server (APID) (Alonso-Lopez et al., 2016), The Biological General Repository for Interaction Datasets (BioGRID) (Chatr-Aryamontri et al., 2015), CancerNet (Meng et al., 2015), CompPPI (Veres et al., 2015), The Database of Interacting Proteins (DIP) (Xenarios et al., 2000), Database of Macromolecular Interactions (DOMMINO) (Kuang et al., 2012), gpDB (Theodoropoulou et al., 2008), GWIDD (Kundrotas et al., 2012), Human Interactome Project (HIP) (Rual et al., 2005), Innatedb (Breuer et al., 2013), IntAct (Hermjakob et al., 2004), and Manually Annotated Targets and Drugs Online Resource (MATADOR) (Gunther et al., 2008). Possibly most relevant is APID which is a collection of protein interactomes for more than 400 organisms based in the integration of known experimentally validated protein-protein physical interactions (PPIs). It covers 375,389 interactions and 29,891 interacting proteins. BioGRID contains genetic and protein interactions curated from the primary biomedical literature for all major model organism species and humans. It contains 1,607,037 proteins and genetic interactions, 28,093 chemical associations and 726,378 post translational modifications (PMT) from major model organism species. CancerNet is a human cancer-specific miRNA-target interactions, protein-protein interactions (PPIs) and functionally synergistic miRNA pairs database. It contains interactions across 33 types of human cancers and also PPI information across 33 main normal tissues and cell types. CompPPI (Veres et al., 2015) stands for compartmentalised PPI database, which provides qualitative information on the interactions, proteins and their localizations for PPI network analysis. For human species, it covers 94,488 proteins, 266,306 localisations and 1,311,184 interactions.
  The DIP database catalogues experimentally determined interactions between proteins. It combines information from a variety of sources to create a single, consistent set of protein-protein interactions. It contains 28,826 proteins, covering 834 organisms, 81,762 interactions (lists protein pairs that are known to interact with each other), results from 81,913 distinct experiments describing an interaction and 8,233 data sources. DOMMINO (Kuang et al., 2012) is based on macromolecular interactions and at time of writing it covers more than 407,000 binary interactions. The gpDB (Theodoropoulou et al., 2008) is a resource for GPCRs, G-proteins, Effectors (molecules) and their interactions. It contains 391 entries relating to G-proteins, 2,738 GPCRs entries and 1,390 effectors with data for 469 species. GWIDD (Kundrotas et al., 2012) is an integrated resource for structural studies of protein-protein interactions on a genome-wide scale covering 126,897 binary interactions, involving 43,976 proteins from 771 different organisms. HIP (Rual et al., 2005) is an open resource on human protein-protein interactome network and covers 11,999 proteins and 74,820 Interactions. Innatedb (Breuer et al., 2013) is a knowledge resource for innate immunity interactions and pathways and covers 27,172 curated interactions and 9,460 curated genes. IntAct (Hermjakob et al., 2004) is a common curation platform for 11 molecular interaction databases and containing 572,063 interactions and 107,900 interactors. MATADOR (Gunther et al., 2008) is a unique resource for protein-chemical interactions and it covers 775 drugs and their interactions with proteins.
- **Omics**
  This category is related to the resources containing datasets derived from *in vitro* highthroughput screening (HTS) studies covering all the datasets in metabolomics, proteomics, genomics, transcriptomics, and fluxomics. This category contains 60 databases^24^. A key example is ArrayExpress (Parkinson et al., 2007) which is an archive of functional genomics data stores data from high-throughput functional genomics experiments and covers 71,472 experiments and 2,311,652 bio assays. It accepts all functional genomics data generated from micoarray or next-generation sequencing (NGS) platforms. The Biochemical Genetic and Genomic (BiGG) database (King et al., 2016) is a resource based on more than 70 genome-scale metabolic networks. Genes in the BiGG models are mapped to NCBI genome annotations, and metabolites are linked to many external databases (KEGG, PubChem, and many more). BioSample (Barrett et al., 2012) contains descriptive information about almost 2 million records (a cell line, a tissue biopsy etc.) encompassing 18,000 species whereas Biostudies is a repository for descriptions of biological studies from large projects (e.g., Blueprint, Europe PubMed Central, Eurocan Platform, and diXa data warehouse) and individuals. Currently it covers 2,552,610 files, 2,824,924 links, 4 projects, and 1,214,179 studies. Cancer GenomicHub is a repository that enables data sharing across cancer genomic studies in support of precision medicine. This database is derived from 40 projects, 61 primary sites, 32,555 cases, 356,381 files, 22,147 genes, and 3,142,246 mutations datasets. The chronic kidney disease database (CKDdb) (Singh et al., 2012) contains multi-omic studies (microRNA, genomics, peptidomics, proteomics, and metabolomics) of chronic kidney disease (CKD), disease-related and diseases leading to this trait. Presently it has differential expression data from 49,395 molecule entries (redundant), of which 16,885 are unique molecules (non-redundant) from 377 manually curated studies of 230 publications. Disnor (Lo surdo et al., 2018), DrugSig (Wu et al., 2017), DisGeNet (Piñero et al., 2017), Drug Gene Interaction Database (DGIdb) (Griffith et al., 2013), Online Mendelian Inheritance in Man (OMIM) (Hamosh et al., 2005) are important resources for exploring the genes and disease domain. Disnor contains information on more than 3,700 disease-pathways and linking ~2,600 disease genes to diseases. Whereas, DrugSig contains information on drug induced gene signature for drug repositioning for more than 1,300 drugs. DisGeNet contains 561,119 gene-disease associations (GDAs), between 17,074 genes and 20,370 diseases, disorders, traits, and clinical or abnormal human phenotypes. DGIdb contains over 40,000 genes and 10,000 drugs involved in over 15,000 drug-gene interactions or belonging to one of 39 potentially druggable gene categories whereas OMIM is a widely used, an Online Catalog of Human Genes and Genetic Disorders and traits. Omics Discovery Index (Omics DI) (Perez-Riverol et al., 2017) is a great resource containing heterogeneous omics data covering 452,800 datasets, 65,200 species, 308,300 genomics, 1,600 tissues, 124,400 transcriptomics, 19 repositories, and 7,300 multiomics datasets. The Human Metabolome Database (HMDB) (Wishart et al., 2017) is a widely used database on small molecule metabolites found in the human body covering 114,110 metabolites entries (both water and lipid soluble) and linked to 5,702 protein sequences. Another useful resource on metabolites is the Metabolomics workbench (Sud et al., 2016), it contains structures and annotations of biologically relevant metabolites (>61,000 entries). MetaMapTox (Fabian et al., 2016) is a licensed resource for metabolite profiles from rat plasma and comprehensive pharmacological and toxicological data. Overall, it covers 25 specific and predictive toxicological mode of action (MoA) in eleven different target organs. RefSeq (Nasko et al., 2018) is a comprehensive, integrated, non-redundant, well-annotated set of sequences, including genomic DNA, 20,905,608 transcripts, and 100,043,962 proteins, and 73,996 organisms. MetaboLights (Kale et al., 2016) contains cross-species, cross-technique and covers metabolite structures and their reference spectra as well as their biological roles, locations and concentrations, and experimental data from metabolic experiments. PharmacoDB (Smirnov et al., 2018) is a cancer pharmacogenomic database covering 7 datasets, 41 tissues, 1,691 cell lines, 19,933 genes, 759 drugs, and 650,894 experiments. TCGA (Weinstein et al., 2013) is another resource on cancer genome data containing an array of molecular alterations underlying 206 cases of adult soft tissue sarcomas. RGED (Zhang et al., 2014) is a database of gene expression profiles in kidney disease which covers 55 RNA-sequence data, 5,299 DNA microarray, 101 cell lines, and 5,253 tissues. Connectivity Map (Lamb et al., 2006) is a very large genome-scale library of cellular signatures that catalogues transcriptional responses to chemical, genetic, and disease perturbation. It contains more than 1 million profiles resulting from perturbations of multiple cell types.
- **Pathways-Based Databases**
  Pathways based toxicity databases are useful in the development of AOPs. This category contains 38 databases^25^. Important pathway based databases include the AOP-wiki or Wiki Pathways, Effectopedia (Vinken et al., 2017), KEGG (Kanehisa and Goto, 2000), Pathway commons (Cerami et al., 2011), Reactome (Croft et al., 2011), PathCards (Belinky et al., 2015), XTalkDB (Sam et al., 2017) etc. Effectopedia is a part of AOP Knowledge Base, which has four platforms–AOP-Wiki, Effectopedia, AOP Xplorer and Intermediate Effects DB. It contains 244 AOPs and 1,806 key events. KEGG is a vast encyclopedia of genes and genomes and classified into many modules as Pathway, Brite, Module, Orthology, Genome, Genes, Compound, Glycan, Reaction, Enzyme, Disease, Drug etc. Pathway Commons is a collection of publicly available pathway data from multiple organisms covering 4,000 pathways and 1.3M interactions. Reactome is a unique database, which includes transformations of entities such as transport from one compartment to another and interaction to form a complex, as well as the chemical transformations of classical biochemistry. Its latest version includes 2,244 human pathways, 12,047 reactions, 10,778 proteins, and 1,948 small molecules. PathCards is an integrated database of human biological pathways and their annotations. In addition, the human pathways are clustered into SuperPaths based on gene content similarity. The other DBs such as Genecards (Safran et al., 2010), MalaCards (Rappaport et al., 2014), and GeneLoc (Rosen et al., 2003) could be useful along with PathCard database. Overall, it contains 1,289 SuperPath entries, consolidated from 12 sources. XTalkDB is based on scientific literature supporting crosstalk between pairs of signaling pathways and presently contains 650 curated pathway pairs, 345 crosstalking pathway pairs, 1697-curated publications.
- **Patent Databases**
  Nine databases were identified relating to patents^26^; these are of paramount importance in drug discovery, drug formulations, production, and marketing of new molecules (Heifets and Jurisica, 2012; Papadatos et al., 2016). In addition, databases which contained information on chemical structure, synthesis, *in vitro* or *in vivo* mode of actions etc. are included. Derwent Discovery^27^ covers patents from 32 countries and contains 141.3 million backward citations (cited), 148.9 million forward citations (citing), 34.5 million literature citations. Whereas, the European Patent Office (EPO)^28^ provides free access to over 100 million patent documents. SureChEMBL (Papadatos et al., 2016) contains compounds chemistry extracted from the full text, images and attachments of patent documents from major patent authorities (WIPO, USPTO and EPO). It contains 17 million compounds extracted from 14 million patent documents.
- **Environmental Databases**
  There are 30 databases with information on the effects of chemcials to the environment and non-human species^29^. This category of databases are related to potential, hazardous, or toxic chemicals, which are ubiquitous in nature and are present in air, water, food, soil, dust consumer goods, and are detected in the human body. The Agency for Toxic Substances and Disease Registry (ATSDR) DB (Johnson, 1995) is maintained by the U.S. Department of Health and Health Services, useful in protecting the communities from harmful health effects related to exposure to natural and man-made hazardous substances. It contains A-Z chemical lists of 275 substances and their toxicological profiles. The Hazardous Substances Data Bank (HSDB) (Fonger, 1995) is another toxicology databank from TOXNET and it covers 5,800 records on human exposure, industrial hygiene, emergency handling procedures, environmental fate, regulatory requirements etc. CEDI^30^ and ADI DB (Sugita et al., 2006) are related to Cumulative Estimated Daily Intakes (CEDIs) and Acceptable Daily Intakes (ADIs) for a large number of food contact substances. The database contains information on over 3,000 substances.
  The U.S. EPA's ECOTOX database^31^ provides information on adverse effects of single chemical stressors to ecologically relevant aquatic and terrestrial species. It contains data relating to 11,655 chemicals, 12,630 species obtained from 48,064 references and 919,123 individual results. The Integrated Risk Information System (IRIS) is another product from U.S. EPA and it contains basic information about the risk assessment for groups of chemicals or complex mixtures. It provides toxicity values for health effects resulting from chronic exposure to more than 500 chemicals: Reference Concentration (RfC), Reference Dose (RfD), Cancer descriptors, Oral slope factor (the slope factor used with administered doses to estimate the probability of increased cancer incidence over a lifetime) etc.
  The European Centre for Ecotoxicology and Toxicology of Chemicals- Human Exposure Assessment Tools Database (ECETOC-heatDB) is a public directory of exposure data sources as well as available tools for exposure. Haz-Map® is a relational database of hazardous chemicals and occupational diseases. The database currently contains 12,086 chemical and biological agents, 240 diseases, 121 findings (signs and symptoms), hazard specific to 261 jobs, 243 job tasks, 54 industrial processes, 624 industries, and 27 non-occupational activities. LINCS (Liu et al., 2015) is a standalone library of molecular signatures describing how different types of cells respond to a variety of chemicals called “perbutagens.” At time of writing, this comprised 391 datasets covering 41,847 small molecules, 1,127 cell types derived from different organs, 978 genes, 1469 proteins/155 peptide probes, and 8 antibodies. Another noteworthy resource is the OECD QSAR Toolbox, a tool for grouping of chemicals for read-across that can be applied to data gap filling. It contains 69 profilers (e.g., DNA binding, protein binding, acute aquatic toxicity, carcinogenicity alerts, *in vitro* and *in vivo* mutagenicity alerts, keratinocyte gene expression, HESS profiler etc.) and 55 databases [CPDB, DART, Ecotox, RepDose, ToxcastDB, ZEBET, developmental toxicity database (CAESAR), EFSA Food Tox Hazard Database, EFSA Genotoxicity, REACH bioaccumulation data, GARDskin database etc.] with over 70,000 chemicals and 2,116,700 data points.
- **Animal Alternative Databases**
  Thirty-nine Animal alternative databases were identified relating to data resources^32^, which assist researchers in complying with the 3Rs philosophy of reduction, refinement and replacement of animal use by. Examples of these databases include: Bibliography on Alternatives to Animal Testing (ALTBIB) (Liebsch et al., 2011) that provides citations from year 2000 to present year. AnimAlt-ZEBET (Grune et al., 2000) is another unique resource, which includes high quality, scientifically recognised 149 alternative methods to standard animal tests in the field of toxicology and pharmacology as well as fundamental research. Bgee provides information on gene expression patterns in 29 animal species, produced from multiple data types such as RNA-Seq, Affymetrix etc. (Bastian et al., 2019) The EURL ECVAM Database service on Alternative Methods to animal experimentation (DB-ALM)^33^ developed by the EU Joint Research Centre (JRC) provides evaluated information on development and applications of advanced and alternative methods to animal experimentation in biomedical sciences and toxicology, both in research and for regulatory purposes. The Tracking System for Alternative Methods toward Regulatory Acceptance (TSAR) database^34^ is useful in identifying alternative non-animal methods that have been proposed for regulatory safety or efficacy testing of chemicals or biological agents such as vaccines. For *in vitro* related DBs, Cellosaurus^35^ knowledge resource on cell lines is a good example covering 109,135 cell lines (81,617 human, 19,451 mouse, 1,952 rat).
  The Fetal Calf Serum Free database (FCS-Free Db)^36^ provides a list of FCS-free media available for specific cell lines or cell types. The International Cell Line Authentication Committee (ICLAC)^37^ provides lists of 4,000 cell lines that are currently known to be cross-contaminated or otherwise misidentified or arising from the work of laboratories and cell line repositories worldwide. LifeMap Discovery®, Cells and Tissues Database (Edgar et al., 2013) covers data in embryonic development, stem cell differentiation, regenerative medicine, and *in vivo* and *in vitro* gene expression data curated from scientific literature and HTS data sources. Non-neoplastic Lesion ATLAS (Schmidt, 2014) is a very useful guide for standardising terminology in toxicological pathology for rodents. Another useful database is Organ System Heterogeneity DB, which provides information on the phenotypic heterogeneity of diseases, drugs and mutations in mouse genes on 26 different organ systems defined in using MedDRA ontology at the SOC (System Organ Class) level. The Interspecies database which helps to select the most appropriate animal model, which is essential for efficient extrapolation of animal data to humans or other animals. It provides information on physiological, anatomical, and biochemical parameters across species.
- **Nanomaterial Toxicity Databases**
  22 databases exist that contain information on the properties and toxicity of nanomaterials or their products^38^. The databases include NANoREG—eNanoMapper database (Jeliazkova et al., 2015), developed by EU FP7 eNanoMapper project and it contains toxicological data for the nanomaterials collected by 85 partners across the world. NHECD (Maimon and Browarnik, 2010) is another free database with its main objectives to study the impact of nanoparticles on health, safety, and environment. It has curated a large and developing collection of published data on environmental and health effects following exposure to nanomaterials. The EU Nano Safety Cluster is an open platform containing the Horizon 2020 projects (e.g., SmartNanoTox, NanoReg II, PATROLS etc.) addressing the safety of materials and technologies enabled by the use of nanoparticles. The Nano- Database^39^ is developed by DTU Environment, the Danish Ecological Council and Danish Consumer Council. It consists of assessments of the nanomaterials used in various consumer products and NanoRisk Cat categorization. There are nearly 3,036 products in this database. The Nano database^40^ created by Nature and Springer. The database contains data on nanomaterials, methods of production, and nano-instruments. The data have been curated from articles, patents, and other scientific sources. StatNano^41^ is another comprehensive database on 7,000 nanotechnology products. It also contains analytical reports on the trend of nanotechnology influence on different industries, nanostructures and nano materials.

## Discussion

With the advances in HTS, chemical synthesis, and biological screening (activity, potency, safety profiles), the number of commercially or publicly available databases containing this information has expanded rapidly. This review has resulted in the compilation of nearly 1,000 databases which have been systematically grouped and classified based on content and potential applications. The databases listed cover many areas including: chemical information, drug screening, toxicity (including toxicity of nanomaterials), ADME, binding, docking, clinical trials, pharmacovigilance, genes, enzymes, interactions, omics, pathways, patent information, environmental exposure, and databases providing information on alternatives to animals.

Criteria for characterising databases were considered as summarised in Table 2. The essential criteria were accessibility, relevance of endpoints, chemical identifiers, acceptable ontology, appropriate (or readily convertible) units, ease of data download in different formats and interoperability. Desirable criteria includes access to metadata (study proptocols and statistics), an assessment of the data quality, ease of use, relevant data description (e.g., classification codes) and currency of data. Ontology based databases were also listed which are useful to integrate the semantic data. For efficient database integration, flexible, and robust APIs are essential to support large datasets. One of the significant bottlenecks in database integration is identifying unique data types to ascertain the overlap between data in two or more databases.

Several factors need to be considered when using the increasing number of data resources for predictive toxicology and other purposes. A very important aspect is the accuracy and uniformity of the identity of the chemical and its chemical structure. Uniform chemical structures are often not included in databases and, on occasions, may even be incorrect. Further complications may arise as different salt forms, enantiomers, or isotopes may not be differentiated. One means to assist in the confirmation of the the identity of chemicals is to include a machine–readable representation of the chemical structure (e.g., SMILES, InChI, SDF), along with the key identifiers, such as InChIkeys. Linked to the need for correct chemical identifiers is the prerequisite for high quality chemical structures to ensure the accuracy, completeness and consistency of the information stored in databases. The process of checking the accuracy, or otherwise, of chemical structures can be undertaken using the InChIs and SMILES representations (amongst others). This helps to avoid incorrect structures by detecting duplicate chemical structures, mismatches between structures, different stereoisomer/tautomer forms, mesomeric effects, hypervalency (atom centre displays valency outside its normal value), and numerous other issues relating to chemical bonds and inorganic elements (Fourches et al., 2010). To standardise and normalise the databases, methods such as chemical structure standardisation (removal of mixtures, inorganics, organometallics, salts, solvents and fragments; normalisation of specific chemotypes/metabolites; treatment of tautomeric forms; removal of duplicates), assigning the unique IDs for the samples, de-duplication of the experimental datasets, validation of omic technologies (Sauer et al., 2017) and avoiding multiple measurements for the same parameters etc. should be applied. One critical step in the standardisation or normalisation procedure is to compile datasets with uniform unit values for a particular parameter derived from heterogonous resources. Uniform units are required to compare and analyse multiple datasets. For example, whilst many data exist for pharmacokinetics properties, there is no or little consistency even in the key parameters, often with discrepancies or differences in units/measured values. However, taking an essential propery such as intrinsic clearance (Clint) measured *in vitro* for enzymes as an example, the variability in units can be corrected and normalised to mL/min/g of protein.

To check the accuracy, completeness, and consistency of the databases, a number of qualitative and quantitative methods can be used. For accuracy, the quality of the data in the databases, i.e., chemical structures, can be confirmed against CAS numbers by cross-referencing with chemical name. The records for the data generated should be mentioned on the website as well as details and results of the validation along with the statistics (number of compounds covered, version number). Consistency can be checked for the data in different version updates of the database, however, this information is seldom provided. Metadata i.e., a representation of supporting data in different formats, are not systematically implemented in databases. Resolving this issue would assist in mapping datasets to each other and save time in finding a relevant study. It is also clear that many data resources contain many of the same data and sources of information. In addition, many of the data published in journals, books, or dataset compilations have been merged in single platforms (e.g., PubChem) which makes searching easier. As such, this has resulted in a great deal of overlap between existing databases and potentially the propagation of errors (i.e., an error being carried forward across data sources due to a lack of manual checking). However, overlap between the databases is not considered a major problem and it could be minimised by clearly identifying the origin of data and using primary sources where possible. Ontologies help in mapping and data integration by providing the syntax for describing classes (or concepts), properties and relationships between classes in the domain of discourse. Mapping between the schema (organisation of data as a blueprint of how the database is constructed) and domain ontologies are an important component for information integration. Whereas, metadata are data that describe the elements in a dataset (e.g., Name of the table, Type of columns, and relationships between them) and help in importing the dataset files from one resource to another or consolidate multiple databases into a single database. For many databases licensing terms and conditions are provided. Clear license information is crucial so that the end user knows what can be done with the metadata. Open licenses for databases encourage access and download of data in a machine-readable format together with their metadata. Some databases apply the license to the whole database or some third party datasests. The end users are recommended to read and understand the licensing information correctly to know whether the contents are provided for commercial or educational research purposes only. The researchers or data owners are also recommended to publish the metadata under a public domain license to ensure wide distribution and reuse. Interoperable databases, i.e., those with a user-friendly, interface, and the capacity for easy interaction and exchange with the other systems, are favoured. The choice of a particular API depends on the size of the database and the programming skills of the developer. Creating databases with greater interoperability will increase their utility and potential application in diverse areas. In addition, database mapping can be defined as the process of identifying key data sources (web pages, flat files, XML-formatted data, directly accessible DBs) by using methods such as ontologies, programming codes, graphical interfaces, and then linking those relevant databases before merging (integration) into a common platform or a single database. Mapping of different databases depends on the technical content and architecture of the datasets. For better mapping and integration easy access to the metadata are preferred. The content accessibility provided by the different databases vary: the majority of databases are open platform enabling searching of scientific data, some facilitate data downloading in different formats. Access to some databases is restricted e.g., commercially available databases.

Looking to the future and potential use of *in silico* resources, the integration of databases with *in silico* tools for predicting the properties or activities of compounds could be useful for early decision making in drug discovery and chemical risk assessment. The computational tools should not be limited to only chemistry or biology but should be able to link chemistry with activity, toxicity and the underlying mechanistic information. In other words, the tools should integrate information on dosimetry, human exposure (*in silico*) and *in vitro* toxicity screening data to provide a better chemical safety risk assessment.

An enormous variety of databases relating to *in silico* toxicology, prediction, and safety assessment are available; other potential uses of these databases include the identification of chemically and/or biologically similar chemicals for read-across purposes. In future development of databases key attributes to consider include data quality, accessibility, ease of downloading the data, chemical space coverage, and range of bioactiviy.

## Author Contributions

GP, MC, and JM were involved in the writing of manuscript. GP, DE, and JF conducted the literature survey and summarised and compiled the databases.

### Conflict of Interest Statement

The authors declare that the research was conducted in the absence of any commercial or financial relationships that could be construed as a potential conflict of interest.

## Footnotes

^1^https://toxnet.nlm.nih.gov/

^2^http://pathguide.org/

^3^https://fairsharing.org/databases/

^4^http://www.vls3d.com/

^5^https://en.wikipedia.org/wiki/List_of_biological_databases

^6^https://www.oxfordjournals.org/nar/database/a/

^7^http://alttox.org/resource-center/databases/ etc

^8^References are included in Supplementary Data Sheet S1, section I.

^9^https://chem-space.com/

^10^https://www.emolecules.com/

^11^http://accelrys.com/

^12^References are included in Supplementary Data Sheet S1, section II.

^13^References are included in Supplementary Data Sheet S1, section III.

^14^http://www.fujitsu.com/jp/group/kyushu/en/solutions/industry/lifescience/admedatabase/

^15^References are included in Supplementary Data Sheet S1, section IV.

^16^References are included in Supplementary Data Sheet S1 section V.

^17^https://dailymed.nlm.nih.gov/dailymed/

^18^https://about.medicinescomplete.com/

^19^https://medlineplus.gov/

^20^https://icd.who.int/browse10/2016/en#/X

^21^https://eudract.ema.europa.eu/

^22^References are included in Supplementary Data Sheet S1, section VI.

^23^References are included in Supplementary Data Sheet S1, section VII.

^24^References are included in Supplementary Data Sheet S1, section VIII.

^25^References are included in Supplementary Data Sheet S1, section IX.

^26^References are included in Supplementary Data Sheet S1, section X.

^27^https://clarivate.com/products/derwent-world-patents-index/

^28^https://www.epo.org/index.html

^29^References are included in Supplementary Data Sheet S1, section XI.

^30^https://www.fda.gov/Food/IngredientsPackagingLabeling/PackagingFCS/CEDI/default.htm

^31^https://cfpub.epa.gov/ecotox/

^32^References are included in Supplementary Data Sheet S1, section XII.

^33^https://ecvam-dbalm.jrc.ec.europa.eu/

^34^https://tsar.jrc.ec.europa.eu/

^35^https://web.expasy.org/cellosaurus/

^36^https://fcs-free.org/

^37^http://iclac.org/

^38^References are included in Supplementary Data Sheet S1, section XIII.

^39^http://nanodb.dk/en/

^40^https://nano.nature.com/

^41^https://statnano.com/
