# Supplementary material for: In Silico Toxicology Data Resources to Support Read-Across and (Q)SAR
Source: Front Pharmacol. 2019 Jun 11;10:561. doi: 10.3389/fphar.2019.00561 (PMC6580867; doi:10.3389/fphar.2019.00561)
Supplement: Supplementary file 1 [file Data_Sheet_1.docx]

**Supplementary sheet-1**

# In Silico Toxicology Data Resources to Support Read-Across and (Q)SAR

Gopal Pawar, Judith C. Madden, David Ebbrell, James W. Firman, Mark T.D. Cronin*

^1^School of Pharmacy and Biomolecular Sciences, Liverpool John Moores University, Byrom Street, Liverpool L3 3AF, England

**References pertaining to each database under different categories**

**I. Chemistry Databases**

1. CFam (C. Zhang et al., 2015)
2. CCDS (Groom & Allen, 2014)
3. ChemAgora Portal (Zanzi & Wittwehr, 2017)
4. ChemSpider (Pence & Williams, 2010)
5. CompTox Chemistry Dashboard (A. J. Williams et al., 2017)
6. ChEBI (Hastings et al., 2015)
7. The Chemical Space Project (Reymond, 2015)
8. COD (Grazulis et al., 2012)
9. ChemIDplus (Tomasulo, 2002)
10. SciFinder (Gabrielson, 2018)
11. ChemDB (J. H. Chen, Linstead, Swamidass, Wang, & Baldi, 2007)
12. ChEMBL (Gaulton et al., 2017)
13. DOCK Blaster (Irwin et al., 2009)
14. Danish QSAR (Pradeep, Povinelli, White, & Merrill, 2016)
15. eChemPortal (de Marcellus, 2014)
16. e-Drug3D (Pihan, Colliandre, Guichou, & Douguet, 2012)
17. eQuilibrator (Flamholz, Noor, Bar-Even, & Milo, 2012)
18. FDB-17 (Visini, Awale, & Reymond, 2017)
19. FilTer Base (Kolte, Londhe, Solanki, Gacche, & Meshram, 2018)
20. GDB-17 (Ruddigkeit, van Deursen, Blum, & Reymond, 2012)
21. HDACiDB (Murugan et al., 2015)
22. IUCLID (Heidorn, Hansen, & Nørager, 1996)
23. JRC-QSAR Model Db (M. Pavan & Worth, 2008)
24. LipidBank (Arita, Yasugi, Seyama, & Nishijima, 2007)
25. Lipidomics Gateway (M. Sud, Fahy, Cotter, Dennis, & Subramaniam, 2012)
26. NCI- DIS-3D Database (Milne, Nicklaus, Driscoll, Wang, & Zaharevitz, 1994)
27. NMRShift DB (S. Kuhn & Schlörer, 2015)
28. OCHEM (Sushko et al., 2011)
29. OSDDChem (Årdal & Røttingen, 2012)
30. PubChem (Hähnke, Kim, & Bolton, 2018)
31. Protein pKa Db (C. P. Toseland, McSparron, Davies, & Flower, 2006)
32. PCDDB (Whitmore, Miles, Mavridis, Janes, & Wallace, 2017)
33. Probes and Drugs Portal (Skuta et al., 2017)
34. Probe Miner (Antolin et al., 2018)
35. SDBS (Saito & Kinugasa, 2011)
36. ZINC 15 (Sterling & Irwin, 2015)

**II. Toxicological Databases**

1. [ACToR](https://actor.epa.gov/actor/home.xhtml) (Judson et al., 2008)
2. AcuteTox (Clemedson, 2008)
3. CPDB (Gold, Manley, Slone, & Rohrbach, 1999)
4. COSMOS (Cronin MTD, 2012)
5. CTD (Davis et al., 2011)
6. CEBS (Lea, Gong, Paleja, Rashid, & Fostel, 2017)
7. ChemTunes & ToxGPS (Fioravanzo et al., 2015; C. Yang et al., 2018)
8. CCRIS (P. Cameron, M. Stump, & Schofield, 2019)
9. DSSTox (Williams-DeVane, Wolf, & Richard, 2009)
10. [diXa](http://www.dixa-fp7.eu/home) (Hendrickx et al., 2014)
11. DevTox (Solecki et al., 2010)
12. Drug Matrix (Ganter, Snyder, Halbert, & Lee, 2006)
13. [eTox](http://www.etoxproject.eu/) (Cases et al., 2014; Sanz et al., 2017)
14. EADB (J. Shen et al., 2013)
15. EDKB (Ding et al., 2010)
16. HESS Database (Abe et al., 2012)
17. ISSTOX (Benigni, Battistelli, Bossa, Tcheremenskaia, & Crettaz, 2013)
18. [Lhasa Carcinogenecity](https://www.lhasalimited.org/Initiatives/lhasa-carcinogenicity-database.htm) Db (Marchant, 1996; Roberts, Myatt, Johnson, Cross, & Blower, 2000)
19. LiverTox (Yu et al., 2014)
20. LTKB (M. Chen et al., 2013)
21. NCI-60 (DTP) (Close et al., 2018)
22. [NCTRIcdb](https://www.fda.gov/ScienceResearch/BioinformaticsTools/ucm236173.htm) (Beger, Young, & Fang, 2004)
23. [Open TG-GATEs](https://toxico.nibiohn.go.jp/english/) (Igarashi et al., 2015)
24. [pCEC](http://project.nies.go.jp/eCA/cgi-bin/index.cgi?page=1) (Sone et al., 2010)
25. ProTox (Priyanka Banerjee, Eckert, Schrey, & Preissner, 2018)
26. SuperToxic (U. Schmidt et al., 2009)
27. ToxLine (Schultheisz, 1981)
28. ToxDB (Hardt et al., 2016)
29. Toxygates (Natsume-Kitatani, Nyström-Persson, Igarashi, Satoh, & Mizuguchi, 2017)
30. [Toxbank](http://www.toxbank.net/) (Kohonen et al., 2013)
31. Tox21 (R. Huang et al., 2016)
32. Toxcast (Richard et al., 2016)
33. ToxRefDB (M. T. Martin et al., 2009)
34. T3DB (D. Wishart et al., 2015)

**III. ADME Databases**

1. ADME-AP (L. Z. Sun, Ji, Chen, Wang, & Chen, 2002)
2. ADMET SAR (F. Cheng et al., 2012)
3. ADMETlab (J. Dong et al., 2018)
4. ADMETNet (Q. Xu et al., 2017)
5. AMED Cardiotoxicity (Sato, Yuki, Ogura, & Honma, 2018)
6. BBB/ HIA database (Jie Shen, Cheng, Xu, Li, & Tang, 2010)
7. CYP-DI table (DA., 2007)
8. CYP-450 Inhibitors (Feixiong Cheng et al., 2011)
9. DIDB (Hachad, Ragueneau-Majlessi, & Levy, 2010)
10. e-pk Gene (Hachad et al., 2011)
11. EDETOX Db (F. Williams, 2004)
12. FINDbase (P. Papadopoulos et al., 2014)
13. IDAAPM (Legehar, Xhaard, & Ghemtio, 2016)
14. Metrabase (Mak et al., 2015)
15. OI-DDI (Yeung et al., 2015)
16. PDSP-Ki (Roth, Lopez, Patel, & Kroeze, 2000)
17. [Tox-database.net](http://tox-portal.net/index.html) (Polak, Wiśniowska, Glinka, & Polak, 2012)
18. TransportDB (Elbourne, Tetu, Hassan, & Paulsen, 2017)
19. TCDB (Saier et al., 2016)
20. TP-Search (Ozawa et al., 2004)
21. TTD (Y. H. Li et al., 2018)
22. Transformer (Hoffmann et al., 2014)
23. UCSF-P’genetics (Kroetz, Yee, & Giacomini, 2009)
24. UCSF-FDA Transportal (Morrissey et al., 2012)
25. X-MetDB (Spjuth, Rydberg, L. Willighagen, Evelo, & Jeliazkova, 2016)

**IV. Drug Discovery Databases**

1. Allosteric Database (Q. Shen et al., 2016)
2. ASDCD (Xing Chen et al., 2014)
3. AffinDB (Block, A Sotriffer, Dramburg, & Klebe, 2006)
4. APD (G. Wang, Li, & Wang, 2016)
5. AutoBind (Darby Tien-Hao Chang, Ke, Lin, & Chiang, 2012)
6. ARDB (B. Liu & Pop, 2009)
7. aBiofilm (Rajput, Thakur, Sharma, & Kumar, 2018)
8. Autosome Chromosome Rearrangement Db (Jie, Lonnie, Peter, & Stephen, 2004)
9. ADHD gene Db (L. Zhang et al., 2012)
10. Allergome Db (Mari, Mari, & Ronconi, 2005)
11. Autism KB (L.-M. Xu et al., 2012)
12. Binding DB (Gilson et al., 2016)
13. Binding MOAD (M. L. Benson et al., 2008)
14. Brenda (Placzek et al., 2017)
15. BioModels (Chelliah, Laibe, & Le Novere, 2013)
16. BCNTB bioinformatics (Gadaleta, Pirrò, Dayem Ullah, Marzec, & Chelala, 2018)
17. BARD (Howe et al., 2015)
18. Big Data Center ("Database Resources of the BIG Data Center in 2019," 2018)
19. BD-gene (S. H. Chang et al., 2013)
20. [CLiBE](http://bidd.nus.edu.sg/group/CLiBE/CLiBE.asp) (X. Chen, Ji, Zhi, & Chen, 2002)
21. CTD^2^ (Aksoy et al., 2017)
22. Cancer Resource (Gohlke, Nickel, Otto, Dunkel, & Preissner, 2016)
23. [canSAR](https://cansar.icr.ac.uk/) (Coker et al., 2018)
24. CARLSBAD (Mathias et al., 2013)
25. CCGD (Abbott et al., 2015)
26. Chemical Probes (Frye, 2010)
27. CARD (B. Jia et al., 2017)
28. cBioPortal (J. Gao et al., 2013)
29. CREDO (Schreyer & Blundell, 2013)
30. CCDB (Agarwal, Raghav, Singh, & Raghava, 2011)
31. CS-DEGs (L. Guo, Du, Chang, Zhang, & Wang, 2014)
32. CAMPR3 (Waghu, Barai, Gurung, & Idicula-Thomas, 2016)
33. Cyclonet (Kolpakov et al., 2007)
34. Cancer DR (R. Kumar et al., 2013)
35. Cell Image Library (Orloff, Iwasa, Martone, Ellisman, & Kane, 2013)
36. DART (Ji et al., 2003)
37. DT-Web (Alaimo et al., 2015)
38. DTome (Jingchun Sun, Wu, Xu, & Zhao, 2012)
39. DRH (Corsello et al., 2017)
40. DTC (Tanoli et al., 2018)
41. Drug Miner (Jamali et al., 2016)
42. DCDB (Y. Liu et al., 2014)
43. DrugBank (David S. Wishart, Knox, et al., 2008)
44. DSigDB (M. Yoo et al., 2015)
45. Drug Central (Ursu et al., 2016)
46. D3R (Gaieb et al., 2018)
47. DBAASP (Pirtskhalava et al., 2016)
48. Drug2Gene (Roider et al., 2014)
49. DGIdb (Cotto et al., 2018)
50. DNASU (Seiler et al., 2014)
51. Driver DBv2 (Chung et al., 2016)
52. DiseaseMeth (Lv et al., 2012)
53. EUPath DB (Aurrecoechea et al., 2017)
54. Ensembl (Kersey et al., 2018)
55. [ExCAPE-DB](https://solr.ideaconsult.net/search/excape/) (Jiangming Sun et al., 2017)
56. e-Drug3D (Pihan et al., 2012)
57. e-HOMD (Fernández Escapa et al., 2018)
58. FaCD Online (Rainville & Garber, 2008)
59. Flow Repository (Spidlen, Breuer, Rosenberg, Kotecha, & Brinkman, 2012)
60. [GenomeCRISPR](http://genomecrispr.dkfz.de/#!/) (Rauscher, Heigwer, Breinig, Winter, & Boutros, 2017)
61. GOLD (Mukherjee et al., 2018)
62. Gene DB (Hertz-Fowler et al., 2004)
63. GLIDA (Okuno, Yang, Taneishi, Yabuuchi, & Tsujimoto, 2006)
64. GDSC (W. Yang et al., 2013)
65. HMDC (J. Eppig, 2017)
66. Human/ Animal TFDB (Hu et al., 2018)
67. IntSide (Juan-Blanco, Duran-Frigola, & Aloy, 2015)
68. ICGC (J. Zhang et al., 2011)
69. KDBI (P. Kumar et al., 2009)
70. KinMutBase (Ortutay, Valiaho, Stenberg, & Vihinen, 2005)
71. KLIFS (Kooistra et al., 2016)
72. LigDig (Fuller et al., 2015)
73. Liver Atlas (Y. Zhang et al., 2013)
74. MetaADEDB (Feixiong Cheng et al., 2013)
75. MetaBase (Bolser et al., 2012)
76. MOSAIC (Nelson et al., 2018)
77. MEDock (Darby Tien-Hau Chang, Oyang, & Lin, 2005)
78. MICAD (Chopra et al., 2012)
79. MTB Db (Krupke et al., 2017)
80. mSignatureDB (P. J. Huang et al., 2018)
81. mutLBSgeneDB (P. Kim, Zhao, Lu, & Zhao, 2017)
82. MK4MDD (L. Guo et al., 2012)
83. Methy Cancer (X. He et al., 2008)
84. NPASS (X. Zeng et al., 2018)
85. NLDB (Murakami, Omori, & Kinoshita, 2016)
86. NCCN (Wood, 2004)
87. NeuroMorpho.Org (Ascoli, Donohue, & Halavi, 2007)
88. NIF (A. Gupta et al., 2008)
89. NPACT (Mangal, Sagar, Singh, Raghava, & Agarwal, 2013)
90. NCG (Kuppili Venkata et al., 2018)
91. Neuron DB (Marenco, Nadkarni, Skoufos, Shepherd, & Miller, 1999)
92. OBO Foundry (B. Smith et al., 2007)
93. OCGD (Gadewal & Zingde, 2011)
94. Open Target (Koscielny et al., 2017)
95. Open PHACTS (A. J. Williams et al., 2012)
96. Orphanet Db (S. Pavan et al., 2017)
97. PDBbind-CN Database (Z. Liu et al., 2015)
98. PoSSuM (Ito, Tabei, Shimizu, Tsuda, & Tomii, 2011)
99. PICKLES (Lenoir, Lim, & Hart, 2018)
100. PhID (Deng, Tu, Deng, & Hu, 2017)
101. PHAROS (Nguyen et al., 2017)
102. PharmGKB (Thorn, Klein, & Altman, 2013)
103. PROMISCUOUS (von Eichborn et al., 2011)
104. PathogenBox (Jeong et al., 2018)
105. RepurposeDB (Shameer et al., 2018)
106. repoDB (Brown & Patel, 2017)
107. RxNav (K. Zeng, Bodenreider, Kilbourne, & Nelson, 2006)
108. Sc-PDB (Desaphy, Bret, Rognan, & Kellenberger, 2015)
109. Super Drug 2.0 (Goede, Dunkel, Mester, Frommel, & Preissner, 2005)
110. Super Target (Hecker et al., 2012)
111. [SM2miR](http://210.46.85.180:8080/sm2mir/index.jsp) (Xinyi Liu et al., 2013)
112. [SuperPred/target/toxic](http://prediction.charite.de/) (Nickel et al., 2014)
113. Super Natural II (P. Banerjee et al., 2015)
114. Super Pain (Gohlke, Preissner, & Preissner, 2014)
115. Swiss Bioisostere (Wirth, Zoete, Michielin, & Sauer, 2012)
116. SIMAP (Arnold, Goldenberg, Mewes, & Rattei, 2014)
117. Swiss Dock (Grosdidier, Zoete, & Michielin, 2011)
118. Swiss SideChain (Gfeller, Michielin, & Zoete, 2012)
119. SFARI Gene (Banerjee-Basu & Packer, 2010)
120. TDR Targets (Magarinos et al., 2012)
121. [TBDTBD](http://www.bioinformatics.org/tbdtdb/) (Rosenthal et al., 2017)
122. THPdb (Usmani et al., 2017)
123. TCMID (Xue et al., 2013)
124. TPDB (Hanzlik, Koen, Theertham, Dong, & Fang, 2007)
125. TTDB (Y. H. Li et al., 2018)
126. TAG (J. S. Chen, Hung, Chan, Tsai, & Sun, 2013)
127. TCMID (Xue et al., 2013)
128. TADB (Hanzlik et al., 2007)
129. TissGDB (P. Kim et al., 2018)
130. VKCDB (Gallin & Boutet, 2011)
131. ViPR (Pickett et al., 2012)

**V. Drug Information/Clinical Trials/ Pharmacovigilance Databases**

1. ATC-DDD (Natsch et al., 1998)
2. ALFRED (Rajeevan et al., 2005)
3. AFND (Gonzalez-Galarza, Christmas, Middleton, & Jones, 2011)
4. AutDB (S. N. Basu, Kollu, & Banerjee-Basu, 2009)
5. BmDR (M. Martin, 2012)
6. BioPortal (Whetzel et al., 2011)
7. BRCA Exchange (Cline et al., 2018)
8. BioLINCC (Ross et al., 2016)
9. BioProject (Barrett et al., 2011)
10. Colorectal Cancer Atlas (Chisanga et al., 2016)
11. CKB (Patterson, Statz, Yin, & Mockus, 2017)
12. Cancer PPD (Tyagi et al., 2015)
13. CVRG (Winslow et al., 2011)
14. CPRD (Herrett et al., 2015)
15. CPIC (Relling & Klein, 2011)
16. Clinical Trials.gov (Schwartz, Woloshin, Zheng, Tse, & Zarin, 2016)
17. COSMIC (Forbes et al., 2008)
18. CTRP (A. Basu et al., 2013)
19. ChemDB (J. Chen, Swamidass, Dou, Bruand, & Baldi, 2005)
20. DAAB (Sircar et al., 2015)
21. DECIPHER (Firth et al., 2009)
22. Drug Trials Snapshots (Whyte, Woodcock, & Wang, 2017)
23. Drug Consumption Db (Ferrer et al., 2012)
24. Disease Ontology (Bello et al., 2018)
25. ENCePP (Kurz, Perez-Gutthann, & Group, 2018)
26. EudraVigilance (Postigo et al., 2018)
27. [EAHD -CF-DB](http://www.factorix.org/) (Rallapalli, Kemball-Cook, Tuddenham, Gomez, & Perkins, 2013)
28. FAERS (Fang et al., 2014)
29. [GePaRD](https://www.bips-institut.de/en/research/research-infrastructures/gepard.html) (Ohlmeier et al., 2015)
30. GHR (Spatz, 2004)
31. [HC-SC (MedEffect)](https://www.canada.ca/en/health-canada/services/drugs-health-products/medeffect-canada.html) (Lexchin, 2014)
32. HPO (Köhler et al., 2018)
33. HEROD (Xian Zeng et al., 2017)
34. ICD-10 (Weatherspoon & Chattopadhyay, 2013)
35. [iSAEC](https://systemsbiology.columbia.edu/isaec) (Contreras, Floratos, & Holden, 2013)
36. IDA (Crawford, Neu, & Toga, 2016)
37. LOINC (Khan et al., 2006)
38. Lareb (Scholl, van Hunsel, Hak, & van Puijenbroek, 2018)
39. MedDRA (J. Harrison & Mozzicato, 2009)
40. Micromedex (Chatfield, 2015)
41. MedWatch (Craigle, 2007)
42. MeSH (J. Nelson, Douglas Johnston, & L. Humphreys, 2001)
43. NDF-RT (Zhu, Freimuth, Pathak, Durski, & Chute, 2013)
44. NAPDI (Paine, Shen, & McCune, 2018)
45. Ontobee (Ong et al., 2017)
46. Open FDA (Kass-Hout et al., 2016)
47. OSB (Gleeson et al., 2018)
48. PanDrugs (Piñeiro-Yáñez et al., 2018)
49. PharmVar (Gaedigk et al., 2018)
50. PillBox (Yaniv et al., 2016)
51. PPMI (Marek et al., 2018)
52. PDX- Finder (Conte et al., 2018)
53. PedAM (J. Jia et al., 2018)
54. RxNorm (Freimuth, Wix, Zhu, Siska, & Chute, 2014)
55. SIDER 4.1 (M. Kuhn, Letunic, Jensen, & Bork, 2016)
56. SNOMED-CT (D. Lee, Cornet, Lau, & de Keizer, 2013)
57. Swiss-Var (Mottaz, David, Veuthey, & Yip, 2010)
58. STRIDE (STARR) (Lowe, Ferris, Hernandez, & Weber, 2009)
59. STEP (Salunke & Tuleu, 2015)
60. SUSARs (Pietraszkiewicz, Firlag-Burkacka, Horban, & Kowalska, 2014)
61. TCIA (Prior et al., 2017)
62. Trialtrove (Stergiopoulos, Getz, & Blazynski)
63. Uberon (Mungall, Torniai, Gkoutos, Lewis, & Haendel, 2012)
64. UMLS (Bodenreider, 2004)
65. VarCards (J. Li et al., 2018)
66. VAERS (Singleton, Lloyd, Mootrey, Salive, & Chen, 1999)
67. Vigibase (Lindquist, 2008)
68. WHO ICTRP (Lindquist, 2008)
69. WITHDRAWN (Siramshetty et al., 2016)

**VI. Biological Databases**

1. AbMiner (Major et al., 2006)
2. AntiJen (Christopher P. Toseland et al., 2005)
3. AlzhCPI (J. Fang et al., 2017)
4. Allen Brain Map Atlas (Sunkin et al., 2013)
5. ABCdb (Fichant, Basse, & Quentin, 2006)
6. AHTPDB (R. Kumar et al., 2015)
7. [ADPriboDB](http://adpribodb.leunglab.org/) (Vivelo, Wat, Agrawal, Tee, & Leung, 2017)
8. AntigenDB (Ansari, Flower, & Raghava, 2010)
9. AAindex (Kawashima et al., 2008)
10. AVPdb (Qureshi, Thakur, Tandon, & Kumar, 2014)
11. Addgene (Kamens, 2015)
12. AlloMAPS (Tan, Tee, Guarnera, Booth, & Berezovsky, 2018)
13. ArrayMap (Tan et al., 2018)
14. AH-DB (Darby Tien-Hao Chang, Yao, Fan, Chiang, & Bai, 2012)
15. ACLAME (Darby Tien-Hao Chang, Yao, et al., 2012)
16. ADHDgene (L. Zhang et al., 2012)
17. BioDBnet (Mudunuri, Che, Yi, & Stephens, 2009)
18. BUSCO (Simao, Waterhouse, Ioannidis, Kriventseva, & Zdobnov, 2015)
19. BrainTranscriptome (Keil, Qalieh, & Kwan, 2018)
20. Broad Bioimage (Ljosa, Sokolnicki, & Carpenter, 2012)
21. BioMuta (Dingerdissen et al., 2018)
22. BDB (B. He et al., 2016)
23. CPDB (Kamburov, Stelzl, Lehrach, & Herwig, 2013)
24. CASBAH (Lüthi & Martin, 2007)
25. CAMEO (Haas et al., 2018)
26. CanEvolve (Samur et al., 2013)
27. CHOPIN (Ochoa-Montano, Mohan, & Blundell, 2015)
28. Cangem (Scheinin et al., 2008)
29. CATH (Dawson et al., 2017)
30. Candidate Cancer Gene (Abbott et al., 2015)
31. CRISPRInc (W. Chen et al., 2018)
32. CirGRDB (X. Li et al., 2018)
33. CCDS (Farrell et al., 2014)
34. DisProt (D. Piovesan et al., 2017)
35. DDBJ (Mashima et al., 2016)
36. dbPTM (K. Y. Huang et al., 2016)
37. Directory of CYP 450 (Fábién & Degtyarenko, 1997)
38. dbSNP (Smigielski, Sirotkin, Ward, & Sherry, 2000)
39. dbGAP (Tryka et al., 2014)
40. dbVar (Ilkka Lappalainen et al., 2012)
41. dbNP (Evelo, van Bochove, & Saito, 2011)
42. DBTSS (Suzuki et al., 2018)
43. DIDA (Gazzo et al., 2016)
44. DDMGD (Raies, Mansour, Incitti, & Bajic, 2015)
45. DEPOD (Duan, Li, & Kohn, 2015)
46. D^2^P^2^ (Oates et al., 2013)
47. ExoCarta (Keerthikumar et al., 2016)
48. ENCODE ("The ENCODE (ENCyclopedia Of DNA Elements) Project," 2004)
49. ExAC (Lek et al., 2016)
50. EnzymePortal (de Matos et al., 2013)
51. EMDB (Patwardhan, 2017)
52. [Eidogen-Sertanty](http://www.eidogen.com/kinasekb.php)- KKB (Brooijmans, Chang, Mobilio, Denny, & Humblet, 2010)
53. ECO (Chibucos et al., 2014)
54. Ensembl (Zerbino et al., 2018)
55. Enzyme (Bairoch, 2000)
56. EGA (Ilkka Lappalainen et al., 2015)
57. ENA (P. W. Harrison et al., 2018)
58. EMPAIR (Bolasco, Weinhard, Boissonnet, Neujahr, & Gross, 2018)
59. eyeGENE (Blain, Goetz, Ayyagari, & Tumminia, 2013)
60. [EuMMCR](https://www.eummcr.org/) (Schick et al., 2016)
61. Ebola/ HVF (Kuiken, Thurmond, Dimitrijevic, & Yoon, 2012)
62. ERGR (A.-Y. Guo et al., 2009)
63. EPDnew (Dreos, Ambrosini, Cavin Périer, & Bucher, 2013)
64. Fraggle (Ahmed et al., 2011)
65. Fusion GDB (P. Kim & Zhou, 2018)
66. GenBank (D. A. Benson et al., 2013)
67. GlycoEpitope (Okuda, Nakao, & Kawasaki, 2021)
68. GLASS (W. K. B. Chan et al., 2015)
69. GEO (Clough & Barrett, 2016)
70. GPCRdb (Pándy-Szekeres et al., 2018)
71. GTEX portal (Consortium, 2013)
72. Genome 3D (Spielmann, Lupiáñez, & Mundlos, 2018)
73. GENT (G. Shin et al., 2011)
74. GO (Gene Ontology, 2015)
75. GENCODE (Frankish et al., 2018)
76. GlyTouCan (Tiemeyer et al., 2017)
77. GtRNAdb (P. P. Chan & Lowe, 2016)
78. GenomeProperties (Richardson et al., 2018)
79. GeneAtlas (Frezal, 1998)
80. GermOnline (Wiederkehr et al., 2004)
81. GPMdb (Fenyo & Beavis, 2015)
82. GenomeRNAi (E. E. Schmidt et al., 2013)
83. HAMAP (Pedruzzi et al., 2015)
84. HGNC (Braschi et al., 2019)
85. Human Protein Atlas (Uhlen et al., 2010)
86. HPRD (S. Peri et al., 2003)
87. Human Genome Project (Gardiner, 2002)
88. HERvd (Paces, Pavlicek, & Paces, 2002)
89. HORDE (Olender, Nativ, & Lancet, 2013)
90. HAGR (de Magalhaes, Costa, & Toussaint, 2005)
91. HEMD (Z. Huang et al., 2012)
92. HPM (M. S. Kim et al., 2014)
93. HIV molecular immunology Db (Los Alamos National, Theoretical, & Biophysics Group, 1995)
94. hPSCreg (Seltmann et al., 2016)
95. HGVD (Higasa et al., 2016)
96. HIstome (Khare et al., 2012)
97. HEDD (Shao et al., 2018)
98. HGVS (Horaitis & Cotton, 2004)
99. H-InvDB (Takeda et al., 2013)
100. iPTMnet (H. Huang et al., 2018)
101. IEDB (Y. Kim et al., 2012)
102. IUPHAR (Sharman et al., 2013)
103. IID (Kotlyar, Pastrello, Sheahan, & Jurisica, 2016)
104. IMGT (Lefranc et al., 2005)
105. InterPro (Mitchell et al., 2018)
106. iProClass (Wu, Huang, Nikolskaya, Hu, & Barker, 2004)
107. ImmPort (Bhattacharya et al., 2018)
108. [InSiGHT variant Db](https://www.insight-group.org/variants/databases/) (Plazzer et al., 2013)
109. IntEnz (Fleischmann et al., 2004)
110. IPD (Maccari et al., 2017)
111. IMGT/GENE-Db (Giudicelli, Chaume, & Lefranc, 2005)
112. [IMGT/mAb-DB](http://www.imgt.org/mAb-DB/) (Poiron et al., 2010)
113. IMOTA (Palmieri et al., 2018)
114. Interferome (Rusinova et al., 2013)
115. IDR (E. Williams et al., 2017)
116. JGI Genome Portal
117. JGA (Kodama et al., 2015)
118. KinWeb (Milanesi et al., 2006)
119. KIDFamMap (Chiu et al., 2013)
120. Labome (Xie, 2017)
121. LGICdb (Le Novere & Changeux, 2001)
122. LIPID MAPS (Fahy et al., 2009)
123. LncRNADisease v2.0 (Z. Bao et al., 2018)
124. LOCATE (Sprenger et al., 2008)
125. MGC (Temple et al., 2009)
126. MITOMAP (Lott et al., 2013)
127. miRWalk (Dweep & Gretz, 2015)
128. MGnify (L Mitchell et al., 2017)
129. MHCBN (Lata, Bhasin, & Raghava, 2009)
130. MitoProteome (Cotter, Guda, Fahy, & Subramaniam, 2004)
131. MEROPS (Rawlings et al., 2018)
132. MultitaskProtDB (Franco-Serrano et al., 2018)
133. MatrixDB (Clerc et al., 2018)
134. MPSTRUC (Stansfeld et al., 2015)
135. miRBase (Kozomara & Griffiths-Jones, 2014)
136. MRMAssayDB (Bhowmick, Mohammed, & Borchers, 2018)
137. MetaGene (Noguchi, Park, & Takagi, 2006)
138. MitoMiner (A. C. Smith & Robinson, 2018)
139. Morphinome (Prokai, Zharikova, & Stevens, 2005)
140. MeDReaders (Guohua Wang et al., 2018)
141. MSDD (Yue et al., 2018)
142. MethHC (W.-Y. Huang et al., 2015)
143. miRandola (Russo et al., 2018)
144. MetalPDB (Putignano, Rosato, Banci, & Andreini, 2018)
145. Microbiome DB (Oliveira et al., 2018)
146. Mitocheck (Cai et al., 2018)
147. Membranome (Lomize, Hage, & Pogozheva, 2018)
148. MetaBase (Pedersen & Bongo, 2017)
149. MINAS (Schnabl, Suter, & Sigel, 2012)
150. MIPModDB (A. B. Gupta et al., 2012)
151. Modomics (Boccaletto et al., 2018)
152. NRR (Martinez et al., 1997)
153. NPD (Dellaire, Farrall, & Bickmore, 2003)
154. NextProt (Gaudet et al., 2017)
155. NURSA (Margolis, Evans, & W O'Malley, 2005)
156. NC-IUBMB (Lilley et al., 1995)
157. NPIDB (Kirsanov et al., 2013)
158. NIH 3D Print Exchange (McCarthy et al., 2014)
159. NONCODE (C. Liu et al., 2005)
160. NATsDB (Y. Zhang et al., 2007)
161. O-GLYCBASE (R. Gupta, Birch, Rapacki, Brunak, & Hansen, 1999)
162. OrthoDB (Kriventseva et al., 2018)
163. OverGeneDB (Rosikiewicz, Suzuki, & Makałowska, 2018)
164. ORDB (Crasto, Marenco, Miller, & Shepherd, 2002)
165. OncoDB.HCC (W.-H. Su et al., 2007)
166. Organelle DB (Wiwatwattana & Kumar, 2005)
167. OpenSNP (Greshake, Bayer, Rausch, & Reda, 2014)
168. OGEE (W.-H. Chen, Lu, Chen, Zhao, & Bork, 2017)
169. PANTHER (Mi, Muruganujan, Ebert, Huang, & Thomas, 2018)
170. PRINTS (Attwood et al., 1997)
171. PSP (Hornbeck et al., 2015)
172. PRO (Natale et al., 2011)
173. Phospho- ELM (Dinkel et al., 2011)
174. Phospho3D (Dinkel et al., 2011)
175. Plasma Proteome (Schwenk et al., 2017)
176. PDBTM (Kozma, Simon, & Tusnády, 2013)
177. Protein Kinase Resource (Petretti & Prigent, 2005)
178. ProtChemSI (Kalinina, Wichmann, Apic, & Russell, 2011)
179. PHI (Takemoto & Aie, 2017)
180. PDB (Berman et al., 2000)
181. PRIDE (Vizcaino et al., 2016)
182. PROXiMATE (Jemimah, Yugandhar, & Michael Gromiha, 2017)
183. PHOSIDA (Gnad, Gunawardena, & Mann, 2011)
184. PSCDB (Amemiya, Koike, Kidera, & Ota, 2012)
185. Proteome Isoelectric Point (Kozlowski, 2017)
186. PED (Marzec et al., 2018)
187. PlasmID (Zuo et al., 2007)
188. PPT- DB (David S. Wishart, Arndt, et al., 2008)
189. Platinum (Pires, Blundell, & Ascher, 2015)
190. Peroxisome DB (Schluter, Real-Chicharro, Gabaldon, Sanchez-Jimenez, & Pujol, 2010)
191. PDBSum (Laskowski, Jabłońska, Pravda, Vařeková, & Thornton, 2018)
192. Pfam (Finn et al., 2014)
193. Polbase (Langhorst, Jack, Reha-Krantz, & Nichols, 2012)
194. PolymiRTS (L. Bao et al., 2007)
195. ProtoNet (Rappoport, Linial, & Linial, 2013)
196. PrimerBank (X. Wang, Spandidos, Wang, & Seed, 2012)
197. ProtCID (X. Wang et al., 2012)
198. Rhea (Morgat et al., 2017)
199. [RoadMapepigenomics](http://www.roadmapepigenomics.org/) (Roadmap Epigenomics et al., 2015)
200. RBPDB (Cook, Kazan, Zuberi, Morris, & Hughes, 2011)
201. RNA Central ("RNAcentral: a hub of information for non-coding RNA sequences," 2018)
202. RegPhos (K.-Y. Huang et al., 2014)
203. REPAIRtoire (Milanowska et al., 2011)
204. RMDB (Cordero, Lucks, & Das, 2012)
205. RAID (Yi et al., 2017)
206. SAGD (Shi et al., 2018)
207. Stanford Tissue Microarray Db (Marinelli et al., 2008)
208. SCDE (Ho Sui et al., 2012)
209. Super Hapten (Günther, Hempel, Dunkel, Rother, & Preissner, 2007)
210. The SysteMHC Atlas (Shao et al., 2018)
211. SNPeffect (De Baets et al., 2012)
212. SiRecords (Ren et al., 2009)
213. SuperFamily (Pandurangan, Stahlhacke, Oates, Smithers, & Gough, 2019)
214. SwissLipids (Aimo et al., 2015)
215. SSDB (Tohsato, Ho, Kyoda, & Onami, 2016)
216. SBCDDB (Newberg, Mann, Mann, Jenkins, & Copeland, 2018)
217. StarBase (J.-H. Li, Liu, Zhou, Qu, & Yang, 2014)
218. SelenoDB (Romagné et al., 2014)
219. SBKB (Gabanyi et al., 2011)
220. SWISS-MODEL (Biasini et al., 2014)
221. SynSysNet (von Eichborn et al., 2013)
222. SynLethDB (J. Guo, Liu, & Zheng, 2016)
223. Sc-PDB (Kellenberger et al., 2006)
224. STRENDA (Swainston et al., 2018)
225. SDAP (Ivanciuc, Schein, & Braun, 2003)
226. SYFPEITHI (Ivanciuc et al., 2003)
227. STRING (Szklarczyk et al., 2017)
228. SM2miR (X. Liu et al., 2013)
229. tRNAdb (Jühling et al., 2009)
230. TSgene (Zhao, Sun, & Zhao, 2013)
231. topPTM (M. G. Su et al., 2014)
232. TP53 (Leroy et al., 2017)
233. TCDB (Saier et al., 2016)
234. TubercuList (Lew, Kapopoulou, Jones, & Cole, 2011)
235. tRFdb (P. Kumar, Mudunuri, Anaya, & Dutta, 2015)
236. THPdb (Usmani et al., 2017)
237. Telomerase (Podlevsky, Bley, Omana, Qi, & Chen, 2008)
238. UniProt (UniProt, 2014)
239. UbiProt (Chernorudskiy et al., 2007)
240. UniProbe Db (Newburger & Bulyk, 2009)
241. ValidatorDB (Sehnal et al., 2015)
242. ViralZone (Masson et al., 2013)
243. VariO (Vihinen, 2014)
244. VDJdb (Shugay et al., 2018)
245. Wnt Db (Nusse & Varmus, 2012)
246. 1000,000 Genomes Project (Turnbull et al., 2018)
247. 3did (Mosca, Céol, Stein, Olivella, & Aloy, 2014)
248. 5S rRNA (Dinman, 2005)

**VII. Protein-Protein Interactions**

1. APID (Alonso-Lopez et al., 2016)
2. BioGRID (Oughtred et al., 2019)
3. BCL2DB (Rech de Laval, Deleage, Aouacheria, & Combet, 2014)
4. CancerNet (Meng et al., 2015)
5. ComPPI (Veres et al., 2015)
6. CAZy (Cantarel et al., 2009)
7. Complex Portal (Meldal et al., 2019)
8. CORUM (Ruepp et al., 2010)
9. Differential Net (Basha, Shpringer, Argov, & Yeger-Lotem, 2018)
10. DynaSIN (Bhardwaj, Abyzov, Clarke, Shou, & Gerstein, 2011)
11. DIP (Salwinski et al., 2004)
12. DOMMINO (Kuang, Dhroso, Han, Shyu, & Korkin, 2016)
13. gpDB (Theodoropoulou, Bagos, Spyropoulos, & Hamodrakas, 2008)
14. GWIDD (Kundrotas, Zhu, & Vakser, 2012)
15. HINT (Das & Yu, 2012)
16. HIP (Rual et al., 2005)
17. HPRD (Suraj Peri et al., 2004)
18. H-Inv DB (Takeda et al., 2013)
19. HCSGD (Q. Dong et al., 2017)
20. Hit Predict (López, Nakai, & Patil, 2015)
21. InnateDB (Breuer et al., 2013)
22. IMEx (Orchard et al., 2012)
23. INstruct (Meyer, Das, Wang, & Yu, 2013)
24. IRView (Fujimori et al., 2012)
25. IntAct (Kerrien et al., 2012)
26. I2D (Kotlyar et al., 2016)
27. IIIDB (Tseng et al., 2015)
28. [iMOTdb](http://caps.ncbs.res.in/imotdb/) (Pugalenthi, Bhaduri, & Sowdhamini, 2006)
29. iRefWeb (Turner et al., 2010)
30. KBDOCK (Ghoorah, Devignes, Smaïl-Tabbone, & Ritchie, 2014)
31. miRTarBase (Chou et al., 2018)
32. MIPS (Pagel et al., 2005)
33. Mentha (Calderone, Castagnoli, & Cesareni, 2013)
34. MATADOR (Günther et al., 2008)
35. MINT (Licata et al., 2012)
36. ORTI (Vafaee et al., 2016)
37. PSMDB (Wallach & Lilien, 2009)
38. PrePPI (Q. C. Zhang, Petrey, Garzón, Deng, & Honig, 2013)
39. PiSITE (Higurashi, Ishida, & Kinoshita, 2009)
40. ProtInDB (Jordan, El-Manzalawy, Dobbs, & Honavar, 2012)
41. ProtChemSI (Kalinina, Wichmann, Apic, & Russell, 2012)
42. PINT (M. D. Kumar & Gromiha, 2006)
43. PIMADb (Mathew & Sowdhamini, 2016)
44. PIPs (McDowall, Scott, & Barton, 2009)
45. Peptide Atlas (Deutsch, 2010)
46. SCOPPI (Winter, Henschel, Kim, & Schroeder, 2006)
47. SKEMPI (Jankauskaite, Jimenez-Garcia, Dapkunas, Fernandez-Recio, & Moal, 2018)
48. SNAPPI-View (Jefferson, Walsh, Roberts, & Barton, 2007)
49. TRIP Db (Shin, Shin, So, Kwon, & Jeon, 2011)
50. UniHI (Kalathur et al., 2014)
51. Wiki-PI (Orii & Ganapathiraju, 2012)
52. 2P2Idb (Basse, Betzi, Morelli, & Roche, 2016)

**VIII.** **OMICS databases**

1. Angiogenes (Müller et al., 2016)
2. Array Express Db (Rustici et al., 2013)
3. ArrayTrack (H. Fang et al., 2017)
4. BiGG Models (King et al., 2016)
5. BioSample (Gostev et al., 2012)
6. BioStudies (Sarkans et al., 2018)
7. BMRDB (Smelter, Astra, & Moseley, 2017)
8. BML-NMR (Ludwig et al., 2012)
9. BioPlex 2.0 (Huttlin et al., 2017)
10. BioSystems (Geer et al., 2010)
11. BioXpress (Wan et al., 2015)
12. C-MAP (Subramanian et al., 2017)
13. ccmGDB (P. Kim, Cheng, Zhao, & Zhao, 2016)
14. CEGA (Dousse, Junier, & Zdobnov, 2016)
15. CGHub (Wilks et al., 2014)
16. CKDdb (Fernandes & Husi, 2017)
17. CTRP (A. Basu et al., 2013)
18. DepMap (Tsherniak et al., 2017)
19. DISNOR (Lo Surdo et al., 2018)
20. DSigDB (Minjae Yoo et al., 2015)
21. DGVa (I. Lappalainen et al., 2013)
22. DRUGSURV (Amelio et al., 2014)
23. DisGeNet (Piñero et al., 2017)
24. DGIdb (Cotto et al., 2018)
25. Expression Atlas (Papatheodorou et al., 2018)
26. Fiehn Lib Db (Fiehn, 2016)
27. GeneCards (Safran et al., 2010)
28. HMA (Pornputtapong, Nookaew, & Nielsen, 2015)
29. HMDB 4.0 (D. S. Wishart et al., 2018)
30. Incardiome KB (Sharma, Deshpande, Ghatge, & Vangala, 2017)
31. IPD (Robinson, Halliwell, McWilliam, Lopez, & Marsh, 2013)
32. KUPKB (Jupp, Klein, Schanstra, & Stevens, 2011)
33. LOMA (Buchkremer et al., 2010)
34. Metabolomics Workbench (Manish Sud et al., 2016)
35. MutAIT (Avancini et al., 2016)
36. MitoProteome (Cotter et al., 2004)
37. [MSigDB](http://software.broadinstitute.org/gsea/msigdb/index.jsp) (Liberzon et al., 2015)
38. MobiDB (Damiano Piovesan et al., 2018)
39. MBROLE (López-Ibáñez, Pazos, & Chagoyen, 2016)
40. NCI-60 (DTP) (Monga & Sausville, 2002)
41. OMIM (Hamosh, Scott, Amberger, Bocchini, & McKusick, 2005)
42. Omics DI (Perez-Riverol et al., 2017)
43. PACdb (Gamazon et al., 2010)
44. PRIDE (Jones & Cote, 2008)
45. PharmacoDB (Smirnov et al., 2018)
46. READDB (Hashemikhabir, Neelamraju, & Janga, 2015)
47. RefSeq (Pruitt, Tatusova, & Maglott, 2007)
48. Rfam (Griffiths-Jones, Bateman, Marshall, Khanna, & Eddy, 2003)
49. RGED (Q. Zhang et al., 2014)
50. Signalink 2 (Fazekas et al., 2013)
51. SIGNOR (Perfetto et al., 2016)
52. TBDRM-db (Sandgren et al., 2009)
53. TCGA (Rau, Flister, Rui, & Auer, 2019)
54. UniCarb-DB (Campbell et al., 2014)
55. UPdb (T. Papadopoulos et al., 2016)

**IX. Pathways Based Databases**

1. AOP-KB (Ives, Campia, Wang, Wittwehr, & Edwards, 2017)
2. Aging Chart (Moskalev et al., 2016)
3. Biocyc (Paley & Karp, 2017)
4. DIMEdb (O'Shea et al., 2018)
5. Effectopedia (Sachana, 2018)
6. Endonet (Potapov et al., 2006)
7. Human Cyc (Trupp et al., 2010)
8. iPAVS (Sreenivasaiah, Rani, Cayetano, Arul, & Kim, 2012)
9. KEGG (Kanehisa, Sato, Furumichi, Morishima, & Tanabe, 2018)
10. MetaCyc (Caspi et al., 2016)
11. MetaboLights (Kale et al., 2016)
12. MetaMapTox (Kamp et al., 2012)
13. MMMP (Mocellin & Rossi, 2008)
14. Nrf2Ome (Türei et al., 2013)
15. PID (Schaefer et al., 2009)
16. PDID (C. Wang et al., 2016)
17. Pathway Commons (Cerami et al., 2011)
18. PathCards (Belinky et al., 2015)
19. PathDIP (Rahmati, Abovsky, Pastrello, & Jurisica, 2017)
20. Pathways Web (Melott, Weinstein, & Broom, 2016)
21. Pathbase (Schofield et al., 2004)
22. Reactome (Croft et al., 2011)
23. MalaCard (Rappaport et al., 2017)
24. yAPOPTOSIS (Wanichthanarak, Cvijovic, Molt, & Petranovic, 2013)
25. SMPDB (Frolkis et al., 2010)
26. STITCH (M. Kuhn, von Mering, Campillos, Jensen, & Bork, 2008)
27. TIGER (X. Liu, Yu, Zack, Zhu, & Qian, 2008)
28. TRANSPATH (Choi et al., 2004)
29. Tri ForC (Miettinen et al., 2018)
30. TCSBN (S. Lee et al., 2018)
31. WikiPathways (Slenter et al., 2018)
32. XTalkDB (Sam, Teel, Tegge, Bharadwaj, & Murali, 2017)

**X. Patent related database**

1. [SCRIPDB](http://dcv.uhnres.utoronto.ca/SCRIPDB/search/) (Heifets & Jurisica, 2012)
2. SureChEMBL (Papadatos et al., 2016)

**XI. Environmental Exposure related database**

1. ATSDR (Kowalski, Anderson, Moore, & Wilder, 2013)
2. ASTDR MRLs (Selene, Chou, Holler, & T De Rosa, 1998)
3. [CEDI/ADI DB](https://www.accessdata.fda.gov/scripts/sda/sdNavigation.cfm?sd=edisrev) (Neltner et al., 2011)
4. EWAG-BBD (Junfeng Gao, Ellis, & Wackett, 2010)
5. Exposome (Neveu et al., 2016)
6. ECOdrug (Verbruggen et al., 2018)
7. EnviPath (Wicker et al., 2016)
8. HeatDB (O'Mahony et al., 2016)
9. HSDB (Fonger, 1995)
10. [Household Products DB](https://hpd.nlm.nih.gov/) (Cooney & Figg, 2018)
11. IRIS (Dourson, 2018)
12. IARC (Pearce et al., 2015)
13. LINCS (Koleti et al., 2018)
14. OECD-QSAR (Fitzpatrick, 2007)
15. PHAROS (Friar & Vittori, 2017)
16. RiskIE (Wullenweber et al., 2008)
17. RITA (Morawietz, Rittinghausen, & Mohr, 1992)

**XII. Animal Alternatives/ Methods**

1. [Atlases-Pathology Images](https://atlases.muni.cz/en/index.html) (Feit, Kempf, Jedlickova, & Burg, 2005)
2. AnimalTFDB (Hu et al., 2018)
3. Bgee (Bastian et al., 2008)
4. Cellosaurus (Bairoch, 2018)
5. CCLE (Barretina et al., 2012)
6. Cell Line Navigator (Krupp et al., 2013)
7. DB-ALM (Adolphe, 1995)
8. FCS-Free- Db (Gstraunthaler, Lindl, & van der Valk, 2013)
9. Humane Endpoint ("How to determine humane endpoints for research animals," 2015)
10. IMPC (Koscielny et al., 2014)
11. ICLAC (Fusenig, Capes-Davis, Bianchini, Sundell, & Lichter, 2017)
12. Interspecies (Bokkers & Slob, 2007)
13. IMSR (J. T. Eppig, Motenko, Richardson, Richards-Smith, & Smith, 2015)
14. IGRhCellID (Shiau, Gu, Chen, Lin, & Jou, 2011)
15. KERIS (P. Li, Tompkins, Xiao, & the Inflammation and Host Response to Injury Large, 2017)
16. LifeMap Discovery®, Cells & Tiss (Edgar et al., 2013)
17. Mouse Atlas of Gene Expression (Siddiqui et al., 2005)
18. NTP nonneoplastic lesion atlas (C. W. Schmidt, 2014)
19. [Organ system heterogeneity DB](http://mips.helmholtz-muenchen.de/Organ_System_Heterogeneity/) (Mannil, Vogt, Prinz, & Campillos, 2015)
20. Rat Genome Db (Shimoyama et al., 2015)
21. ZFIN (Sprague et al., 2006)

**XIII. Nano Materials Toxicity**

1. [caNanoLab](https://cananolab.nci.nih.gov/caNanoLab/#/) (Morris, Gaheen, Lijowski, Heiskanen, & Klemm, 2015)
2. DaNa (Krug et al., 2018)
3. Good Nano Guide (Kulinowski & Jaffe, 2009)
4. eNanoMapper (Jeliazkova et al., 2015)
5. NHECD (Maimon & Browarnik, 2010)
6. NanoHub (Madhavan et al., 2013)
7. NECID (Oberbek, 2018)

Abbott, K. L., Nyre, E. T., Abrahante, J., Ho, Y. Y., Isaksson Vogel, R., & Starr, T. K. (2015). The Candidate Cancer Gene Database: a database of cancer driver genes from forward genetic screens in mice. *Nucleic Acids Res, 43*(Database issue), D844-848. doi:10.1093/nar/gku770

Abe, T., Kobayashi, K., Nishikawa, S., Yamada, T., Hasegawa, R., Sakuratani, Y., . . . Hayashi, M. (2012). *Development of Hazard Evaluation Support System Database (HESS DB)* (Vol. 12).

Adolphe, M. (1995). [Alternative methods to animal experimentation. Scientific and ethical problems]. *Bull Acad Natl Med, 179*(6), 1009-1016; discussion 1116-1009.

Agarwal, S. M., Raghav, D., Singh, H., & Raghava, G. P. S. (2011). CCDB: a curated database of genes involved in cervix cancer. *Nucleic Acids Research, 39*(suppl_1), D975-D979. doi:10.1093/nar/gkq1024

Ahmed, J., Worth, C. L., Thaben, P., Matzig, C., Blasse, C., Dunkel, M., & Preissner, R. (2011). FragmentStore--a comprehensive database of fragments linking metabolites, toxic molecules and drugs. *Nucleic Acids Research, 39*(Database issue), D1049-D1054. doi:10.1093/nar/gkq969

Aimo, L., Liechti, R., Hyka-Nouspikel, N., Niknejad, A., Gleizes, A., Götz, L., . . . Bridge, A. (2015). The SwissLipids knowledgebase for lipid biology. *Bioinformatics, 31*(17), 2860-2866. doi:10.1093/bioinformatics/btv285

Aksoy, B. A., Dančík, V., Smith, K., Mazerik, J. N., Ji, Z., Gross, B., . . . Clemons, P. A. (2017). CTD2 Dashboard: a searchable web interface to connect validated results from the Cancer Target Discovery and Development Network. *Database, 2017*, bax054-bax054. doi:10.1093/database/bax054

Alaimo, S., Bonnici, V., Cancemi, D., Ferro, A., Giugno, R., & Pulvirenti, A. (2015). DT-Web: a web-based application for drug-target interaction and drug combination prediction through domain-tuned network-based inference. *BMC Syst Biol, 9*(3), S4. doi:10.1186/1752-0509-9-s3-s4

Alonso-Lopez, D., Gutierrez, M. A., Lopes, K. P., Prieto, C., Santamaria, R., & De Las Rivas, J. (2016). APID interactomes: providing proteome-based interactomes with controlled quality for multiple species and derived networks. *Nucleic Acids Res, 44*(W1), W529-535. doi:10.1093/nar/gkw363

Amelio, I., Gostev, M., Knight, R. A., Willis, A. E., Melino, G., & Antonov, A. V. (2014). DRUGSURV: a resource for repositioning of approved and experimental drugs in oncology based on patient survival information. *Cell death & disease, 5*(2), e1051-e1051. doi:10.1038/cddis.2014.9

Amemiya, T., Koike, R., Kidera, A., & Ota, M. (2012). PSCDB: a database for protein structural change upon ligand binding. *Nucleic Acids Res, 40*(Database issue), D554-558. doi:10.1093/nar/gkr966

Ansari, H. R., Flower, D. R., & Raghava, G. P. S. (2010). AntigenDB: an immunoinformatics database of pathogen antigens. *Nucleic Acids Research, 38*(Database issue), D847-D853. doi:10.1093/nar/gkp830

Antolin, A. A., Tym, J. E., Komianou, A., Collins, I., Workman, P., & Al-Lazikani, B. (2018). Objective, Quantitative, Data-Driven Assessment of Chemical Probes. *Cell Chem Biol, 25*(2), 194-205.e195. doi:10.1016/j.chembiol.2017.11.004

Årdal, C., & Røttingen, J.-A. (2012). Open source drug discovery in practice: a case study. *PLoS neglected tropical diseases, 6*(9), e1827-e1827. doi:10.1371/journal.pntd.0001827

Arita, M., Yasugi, E., Seyama, Y., & Nishijima, M. (2007). LipidBank: Its recent changes. *Chemistry and Physics of Lipids, 149*, S74-S75. doi:<https://doi.org/10.1016/j.chemphyslip.2007.06.170>

Arnold, R., Goldenberg, F., Mewes, H.-W., & Rattei, T. (2014). SIMAP--the database of all-against-all protein sequence similarities and annotations with new interfaces and increased coverage. *Nucleic Acids Research, 42*(Database issue), D279-D284. doi:10.1093/nar/gkt970

Ascoli, G. A., Donohue, D. E., & Halavi, M. (2007). NeuroMorpho.Org: A Central Resource for Neuronal Morphologies. *The Journal of Neuroscience, 27*(35), 9247-9251. doi:10.1523/jneurosci.2055-07.2007

Attwood, T. K., Avison, H., Beck, M. E., Bewley, M., Bleasby, A. J., Brewster, F., . . . Worledge, C. (1997). The PRINTS database of protein fingerprints: a novel information resource for computational molecular biology. *J Chem Inf Comput Sci, 37*(3), 417-424.

Aurrecoechea, C., Barreto, A., Basenko, E. Y., Brestelli, J., Brunk, B. P., Cade, S., . . . Zheng, J. (2017). EuPathDB: the eukaryotic pathogen genomics database resource. *Nucleic Acids Research, 45*(D1), D581-D591. doi:10.1093/nar/gkw1105

Avancini, D., Menzies, G. E., Morgan, C., Wills, J., Johnson, G. E., White, P. A., & Lewis, P. D. (2016). MutAIT: an online genetic toxicology data portal and analysis tools. *Mutagenesis, 31*(3), 323-328. doi:10.1093/mutage/gev050

Bairoch, A. (2000). The ENZYME database in 2000. *Nucleic Acids Research, 28*(1), 304-305. doi:10.1093/nar/28.1.304

Bairoch, A. (2018). The Cellosaurus, a Cell-Line Knowledge Resource. *Journal of biomolecular techniques : JBT, 29*(2), 25-38. doi:10.7171/jbt.18-2902-002

Banerjee-Basu, S., & Packer, A. (2010). SFARI Gene: an evolving database for the autism research community. *Disease Models &amp; Mechanisms, 3*(3-4), 133-135. doi:10.1242/dmm.005439

Banerjee, P., Eckert, A. O., Schrey, A. K., & Preissner, R. (2018). ProTox-II: a webserver for the prediction of toxicity of chemicals. *Nucleic Acids Research, 46*(W1), W257-W263. doi:10.1093/nar/gky318

Banerjee, P., Erehman, J., Gohlke, B. O., Wilhelm, T., Preissner, R., & Dunkel, M. (2015). Super Natural II--a database of natural products. *Nucleic Acids Res, 43*(Database issue), D935-939. doi:10.1093/nar/gku886

Bao, L., Zhou, M., Wu, L., Lu, L., Goldowitz, D., Williams, R. W., & Cui, Y. (2007). PolymiRTS Database: linking polymorphisms in microRNA target sites with complex traits. *Nucleic Acids Research, 35*(suppl_1), D51-D54. doi:10.1093/nar/gkl797

Bao, Z., Yang, Z., Huang, Z., Zhou, Y., Cui, Q., & Dong, D. (2018). LncRNADisease 2.0: an updated database of long non-coding RNA-associated diseases. *Nucleic Acids Res*. doi:10.1093/nar/gky905

Barretina, J., Caponigro, G., Stransky, N., Venkatesan, K., Margolin, A. A., Kim, S., . . . Garraway, L. A. (2012). The Cancer Cell Line Encyclopedia enables predictive modelling of anticancer drug sensitivity. *Nature, 483*, 603. doi:10.1038/nature11003

Barrett, T., Clark, K., Gevorgyan, R., Gorelenkov, V., Gribov, E., Karsch-Mizrachi, I., . . . Ostell, J. (2011). *BioProject and BioSample databases at NCBI: Facilitating capture and organization of metadata* (Vol. 40).

Basha, O., Shpringer, R., Argov, C. M., & Yeger-Lotem, E. (2018). The DifferentialNet database of differential protein-protein interactions in human tissues. *Nucleic Acids Res, 46*(D1), D522-d526. doi:10.1093/nar/gkx981

Basse, M.-J., Betzi, S., Morelli, X., & Roche, P. (2016). 2P2Idb v2: update of a structural database dedicated to orthosteric modulation of protein–protein interactions. *Database, 2016*, baw007-baw007. doi:10.1093/database/baw007

Bastian, F., Parmentier, G., Roux, J., Moretti, S., Laudet, V., & Robinson-Rechavi, M. (2008). *Bgee: Integrating and Comparing Heterogeneous Transcriptome Data Among Species* (Vol. 5109).

Basu, A., Bodycombe, N. E., Cheah, J. H., Price, E. V., Liu, K., Schaefer, G. I., . . . Schreiber, S. L. (2013). An interactive resource to identify cancer genetic and lineage dependencies targeted by small molecules. *Cell, 154*(5), 1151-1161. doi:10.1016/j.cell.2013.08.003

Basu, S. N., Kollu, R., & Banerjee-Basu, S. (2009). AutDB: a gene reference resource for autism research. *Nucleic Acids Res, 37*(Database issue), D832-836. doi:10.1093/nar/gkn835

Beger, R. D., Young, J. F., & Fang, H. (2004). Discriminant function analyses of liver-specific carcinogens. *J Chem Inf Comput Sci, 44*(3), 1107-1110. doi:10.1021/ci0342829

Belinky, F., Nativ, N., Stelzer, G., Zimmerman, S., Iny Stein, T., Safran, M., & Lancet, D. (2015). PathCards: multi-source consolidation of human biological pathways. *Database (Oxford), 2015*, bav006. doi:10.1093/database/bav006

Bello, S. M., Shimoyama, M., Mitraka, E., Laulederkind, S. J. F., Smith, C. L., Eppig, J. T., & Schriml, L. M. (2018). Disease Ontology: improving and unifying disease annotations across species. *11*(3), dmm032839. doi:10.1242/dmm.032839 %J Disease Models &amp; Mechanisms

Benigni, R., Battistelli, C. L., Bossa, C., Tcheremenskaia, O., & Crettaz, P. (2013). New perspectives in toxicological information management, and the role of ISSTOX databases in assessing chemical mutagenicity and carcinogenicity. *Mutagenesis, 28*(4), 401-409. doi:10.1093/mutage/get016

Benson, D. A., Cavanaugh, M., Clark, K., Karsch-Mizrachi, I., Lipman, D. J., Ostell, J., & Sayers, E. W. (2013). GenBank. *Nucleic Acids Res, 41*(Database issue), D36-42. doi:10.1093/nar/gks1195

Benson, M. L., Smith, R. D., Khazanov, N. A., Dimcheff, B., Beaver, J., Dresslar, P., . . . Carlson, H. A. (2008). Binding MOAD, a high-quality protein-ligand database. *Nucleic Acids Research, 36*(Database issue), D674-D678. doi:10.1093/nar/gkm911

Berman, H. M., Westbrook, J., Feng, Z., Gilliland, G., Bhat, T. N., Weissig, H., . . . Bourne, P. E. (2000). The Protein Data Bank. *Nucleic Acids Research, 28*(1), 235-242. doi:10.1093/nar/28.1.235

Bhardwaj, N., Abyzov, A., Clarke, D., Shou, C., & Gerstein, M. B. (2011). Integration of protein motions with molecular networks reveals different mechanisms for permanent and transient interactions. *Protein Sci, 20*(10), 1745-1754. doi:10.1002/pro.710

Bhattacharya, S., Dunn, P., Thomas, C. G., Smith, B., Schaefer, H., Chen, J., . . . Butte, A. J. (2018). ImmPort, toward repurposing of open access immunological assay data for translational and clinical research. *Scientific Data, 5*, 180015. doi:10.1038/sdata.2018.15

Bhowmick, P., Mohammed, Y., & Borchers, C. H. (2018). MRMAssayDB: an integrated resource for validated targeted proteomics assays. *Bioinformatics, 34*(20), 3566-3571. doi:10.1093/bioinformatics/bty385

Biasini, M., Bienert, S., Waterhouse, A., Arnold, K., Studer, G., Schmidt, T., . . . Schwede, T. (2014). SWISS-MODEL: modelling protein tertiary and quaternary structure using evolutionary information. *Nucleic Acids Research, 42*(Web Server issue), W252-W258. doi:10.1093/nar/gku340

Blain, D., Goetz, K. E., Ayyagari, R., & Tumminia, S. J. (2013). eyeGENE®: a vision community resource facilitating patient care and paving the path for research through molecular diagnostic testing. *Clinical genetics, 84*(2), 190-197. doi:10.1111/cge.12193

Block, P., A Sotriffer, C., Dramburg, I., & Klebe, G. (2006). *AffinDB: a freely accessible database of affinities for protein-ligand complexes from the PDB* (Vol. 34).

Boccaletto, P., Machnicka, M. A., Purta, E., Piatkowski, P., Baginski, B., Wirecki, T. K., . . . Bujnicki, J. M. (2018). MODOMICS: a database of RNA modification pathways. 2017 update. *Nucleic Acids Res, 46*(D1), D303-d307. doi:10.1093/nar/gkx1030

Bodenreider, O. (2004). The Unified Medical Language System (UMLS): integrating biomedical terminology. *Nucleic Acids Research, 32*(suppl_1), D267-D270. doi:10.1093/nar/gkh061

Bokkers, B. G., & Slob, W. (2007). Deriving a data-based interspecies assessment factor using the NOAEL and the benchmark dose approach. *Crit Rev Toxicol, 37*(5), 355-373. doi:10.1080/10408440701249224

Bolasco, G., Weinhard, L., Boissonnet, T., Neujahr, R., & Gross, C. T. (2018). Three-Dimensional Nanostructure of an Intact Microglia Cell. *Frontiers in neuroanatomy, 12*, 105. doi:10.3389/fnana.2018.00105

Bolser, D. M., Chibon, P.-Y., Palopoli, N., Gong, S., Jacob, D., Del Angel, V. D., . . . Bhak, J. (2012). MetaBase--the wiki-database of biological databases. *Nucleic Acids Research, 40*(Database issue), D1250-D1254. doi:10.1093/nar/gkr1099

Braschi, B., Denny, P., Gray, K., Jones, T., Seal, R., Tweedie, S., . . . Bruford, E. (2019). Genenames.org: the HGNC and VGNC resources in 2019. *Nucleic Acids Research, 47*(D1), D786-D792. doi:10.1093/nar/gky930

Breuer, K., Foroushani, A. K., Laird, M. R., Chen, C., Sribnaia, A., Lo, R., . . . Lynn, D. J. (2013). InnateDB: systems biology of innate immunity and beyond--recent updates and continuing curation. *Nucleic Acids Research, 41*(Database issue), D1228-D1233. doi:10.1093/nar/gks1147

Brooijmans, N., Chang, Y.-W., Mobilio, D., Denny, R. A., & Humblet, C. (2010). An enriched structural kinase database to enable kinome-wide structure-based analyses and drug discovery. *Protein science : a publication of the Protein Society, 19*(4), 763-774. doi:10.1002/pro.355

Brown, A. S., & Patel, C. J. (2017). A standard database for drug repositioning. *Scientific Data, 4*, 170029. doi:10.1038/sdata.2017.29

Buchkremer, S., Hendel, J., Krupp, M., Weinmann, A., Schlamp, K., Maass, T., . . . Teufel, A. J. B. G. (2010). Library of molecular associations: curating the complex molecular basis of liver diseases. *11*(1), 189. doi:10.1186/1471-2164-11-189

Cai, Y., Hossain, M. J., Hériché, J.-K., Politi, A. Z., Walther, N., Koch, B., . . . Ellenberg, J. (2018). Experimental and computational framework for a dynamic protein atlas of human cell division. *Nature, 561*(7723), 411-415. doi:10.1038/s41586-018-0518-z

Calderone, A., Castagnoli, L., & Cesareni, G. (2013). mentha: a resource for browsing integrated protein-interaction networks. *Nat Methods, 10*(8), 690-691. doi:10.1038/nmeth.2561

Campbell, M. P., Nguyen-Khuong, T., Hayes, C. A., Flowers, S. A., Alagesan, K., Kolarich, D., . . . Karlsson, N. G. (2014). Validation of the curation pipeline of UniCarb-DB: Building a global glycan reference MS/MS repository. *Biochimica et Biophysica Acta (BBA) - Proteins and Proteomics, 1844*(1, Part A), 108-116. doi:<https://doi.org/10.1016/j.bbapap.2013.04.018>

Cantarel, B. L., Coutinho, P. M., Rancurel, C., Bernard, T., Lombard, V., & Henrissat, B. (2009). The Carbohydrate-Active EnZymes database (CAZy): an expert resource for Glycogenomics. *Nucleic Acids Research, 37*(Database issue), D233-D238. doi:10.1093/nar/gkn663

Cases, M., Briggs, K., Steger-Hartmann, T., Pognan, F., Marc, P., Kleinoder, T., . . . Sanz, F. (2014). The eTOX data-sharing project to advance in silico drug-induced toxicity prediction. *Int J Mol Sci, 15*(11), 21136-21154. doi:10.3390/ijms151121136

Caspi, R., Billington, R., Ferrer, L., Foerster, H., Fulcher, C. A., Keseler, I. M., . . . Karp, P. D. (2016). The MetaCyc database of metabolic pathways and enzymes and the BioCyc collection of pathway/genome databases. *Nucleic Acids Research, 44*(D1), D471-D480. doi:10.1093/nar/gkv1164

Cerami, E. G., Gross, B. E., Demir, E., Rodchenkov, I., Babur, O., Anwar, N., . . . Sander, C. (2011). Pathway Commons, a web resource for biological pathway data. *Nucleic Acids Research, 39*(Database issue), D685-D690. doi:10.1093/nar/gkq1039

Chan, P. P., & Lowe, T. M. (2016). GtRNAdb 2.0: an expanded database of transfer RNA genes identified in complete and draft genomes. *Nucleic Acids Research, 44*(D1), D184-D189. doi:10.1093/nar/gkv1309

Chan, W. K. B., Zhang, H., Yang, J., Brender, J. R., Hur, J., Özgür, A., & Zhang, Y. (2015). GLASS: a comprehensive database for experimentally validated GPCR-ligand associations. *Bioinformatics, 31*(18), 3035-3042. doi:10.1093/bioinformatics/btv302

Chang, D. T.-H., Ke, C.-H., Lin, J.-H., & Chiang, J.-H. (2012). AutoBind: automatic extraction of protein–ligand-binding affinity data from biological literature. *Bioinformatics, 28*(16), 2162-2168. doi:10.1093/bioinformatics/bts367

Chang, D. T.-H., Oyang, Y.-J., & Lin, J.-H. (2005). MEDock: a web server for efficient prediction of ligand binding sites based on a novel optimization algorithm. *Nucleic Acids Research, 33*(suppl_2), W233-W238. doi:10.1093/nar/gki586

Chang, D. T.-H., Yao, T.-J., Fan, C.-Y., Chiang, C.-Y., & Bai, Y.-H. (2012). AH-DB: collecting protein structure pairs before and after binding. *Nucleic Acids Research, 40*(D1), D472-D478. doi:10.1093/nar/gkr940

Chang, S. H., Gao, L., Li, Z., Zhang, W. N., Du, Y., & Wang, J. (2013). BDgene: a genetic database for bipolar disorder and its overlap with schizophrenia and major depressive disorder. *Biol Psychiatry, 74*(10), 727-733. doi:10.1016/j.biopsych.2013.04.016

Chatfield, A. J. (2015). Lexicomp Online and Micromedex 2.0. *Journal of the Medical Library Association : JMLA, 103*(2), 112-113. doi:10.3163/1536-5050.103.2.016

Chelliah, V., Laibe, C., & Le Novere, N. (2013). BioModels Database: a repository of mathematical models of biological processes. *Methods Mol Biol, 1021*, 189-199. doi:10.1007/978-1-62703-450-0_10

Chen, J., Swamidass, S. J., Dou, Y., Bruand, J., & Baldi, P. (2005). ChemDB: a public database of small molecules and related chemoinformatics resources. *Bioinformatics, 21*(22), 4133-4139. doi:10.1093/bioinformatics/bti683

Chen, J. H., Linstead, E., Swamidass, S. J., Wang, D., & Baldi, P. (2007). ChemDB update—full-text search and virtual chemical space. *Bioinformatics, 23*(17), 2348-2351. doi:10.1093/bioinformatics/btm341

Chen, J. S., Hung, W. S., Chan, H. H., Tsai, S. J., & Sun, H. S. (2013). In silico identification of oncogenic potential of fyn-related kinase in hepatocellular carcinoma. *Bioinformatics, 29*(4), 420-427. doi:10.1093/bioinformatics/bts715

Chen, M., Zhang, J., Wang, Y., Liu, Z., Kelly, R., Zhou, G., . . . Tong, W. (2013). *The Liver Toxicity Knowledge Base: A Systems Approach to a Complex End Point* (Vol. 93).

Chen, W.-H., Lu, G., Chen, X., Zhao, X.-M., & Bork, P. (2017). OGEE v2: an update of the online gene essentiality database with special focus on differentially essential genes in human cancer cell lines. *Nucleic Acids Research, 45*(D1), D940-D944. doi:10.1093/nar/gkw1013

Chen, W., Zhang, G., Li, J., Zhang, X., Huang, S., Xiang, S., . . . Liu, C. (2018). CRISPRlnc: a manually curated database of validated sgRNAs for lncRNAs. *Nucleic Acids Research*, gky904-gky904. doi:10.1093/nar/gky904

Chen, X., Ji, Z. L., Zhi, D. G., & Chen, Y. Z. (2002). CLiBE: a database of computed ligand binding energy for ligand–receptor complexes. *Computers & Chemistry, 26*(6), 661-666. doi:<https://doi.org/10.1016/S0097-8485(02)00050-5>

Chen, X., Ren, B., Chen, M., Liu, M.-X., Ren, W., Wang, Q.-X., . . . Yan, G.-Y. (2014). ASDCD: Antifungal Synergistic Drug Combination Database. *PloS one, 9*(1), e86499. doi:10.1371/journal.pone.0086499

Cheng, F., Li, W., Wang, X., Zhou, Y., Wu, Z., Shen, J., & Tang, Y. (2013). Adverse Drug Events: Database Construction and in Silico Prediction. *Journal of Chemical Information and Modeling, 53*(4), 744-752. doi:10.1021/ci4000079

Cheng, F., Li, W., Zhou, Y., Shen, J., Wu, Z., Liu, G., . . . Tang, Y. (2012). admetSAR: a comprehensive source and free tool for assessment of chemical ADMET properties. *J Chem Inf Model, 52*(11), 3099-3105. doi:10.1021/ci300367a

Cheng, F., Yu, Y., Shen, J., Yang, L., Li, W., Liu, G., . . . Tang, Y. (2011). Classification of Cytochrome P450 Inhibitors and Noninhibitors Using Combined Classifiers. *Journal of Chemical Information and Modeling, 51*(5), 996-1011. doi:10.1021/ci200028n

Chernorudskiy, A. L., Garcia, A., Eremin, E. V., Shorina, A. S., Kondratieva, E. V., & Gainullin, M. R. (2007). UbiProt: a database of ubiquitylated proteins. *BMC Bioinformatics, 8*, 126-126. doi:10.1186/1471-2105-8-126

Chibucos, M. C., Mungall, C. J., Balakrishnan, R., Christie, K. R., Huntley, R. P., White, O., . . . Giglio, M. (2014). Standardized description of scientific evidence using the Evidence Ontology (ECO). *Database (Oxford), 2014*, bau075. doi:10.1093/database/bau075

Chisanga, D., Keerthikumar, S., Pathan, M., Ariyaratne, D., Kalra, H., Boukouris, S., . . . Mathivanan, S. (2016). Colorectal cancer atlas: An integrative resource for genomic and proteomic annotations from colorectal cancer cell lines and tissues. *Nucleic Acids Research, 44*(D1), D969-D974. doi:10.1093/nar/gkv1097

Chiu, Y. Y., Lin, C. T., Huang, J. W., Hsu, K. C., Tseng, J. H., You, S. R., & Yang, J. M. (2013). KIDFamMap: a database of kinase-inhibitor-disease family maps for kinase inhibitor selectivity and binding mechanisms. *Nucleic Acids Res, 41*(Database issue), D430-440. doi:10.1093/nar/gks1218

Choi, C., Krull, M., Kel, A., Kel-Margoulis, O., Pistor, S., Potapov, A., . . . Wingender, E. (2004). TRANSPATH--a high quality database focused on signal transduction. *Comparative and functional genomics, 5*(2), 163-168. doi:10.1002/cfg.386

Chopra, A., Shan, L., Eckelman, W. C., Leung, K., Latterner, M., Bryant, S. H., & Menkens, A. (2012). Molecular Imaging and Contrast Agent Database (MICAD): evolution and progress. *Mol Imaging Biol, 14*(1), 4-13. doi:10.1007/s11307-011-0521-3

Chou, C. H., Shrestha, S., Yang, C. D., Chang, N. W., Lin, Y. L., Liao, K. W., . . . Huang, H. D. (2018). miRTarBase update 2018: a resource for experimentally validated microRNA-target interactions. *Nucleic Acids Res, 46*(D1), D296-d302. doi:10.1093/nar/gkx1067

Chung, I. F., Chen, C.-Y., Su, S.-C., Li, C.-Y., Wu, K.-J., Wang, H.-W., & Cheng, W.-C. (2016). DriverDBv2: a database for human cancer driver gene research. *Nucleic Acids Research, 44*(D1), D975-D979. doi:10.1093/nar/gkv1314

Clemedson, C. (2008). The European ACuteTox project: a modern integrative in vitro approach to better prediction of acute toxicity. *Clin Pharmacol Ther, 84*(2), 200-202. doi:10.1038/clpt.2008.135

Clerc, O., Deniaud, M., Vallet, S. D., Naba, A., Rivet, A., Perez, S., . . . Ricard-Blum, S. (2018). MatrixDB: integration of new data with a focus on glycosaminoglycan interactions. *Nucleic Acids Res*. doi:10.1093/nar/gky1035

Cline, M. S., Liao, R. G., Parsons, M. T., Paten, B., Alquaddoomi, F., Antoniou, A., . . . Spurdle, A. B. (2018). BRCA Challenge: BRCA Exchange as a global resource for variants in BRCA1 and BRCA2. *PLoS Genet, 14*(12), e1007752. doi:10.1371/journal.pgen.1007752

Close, D. A., Wang, A. X., Kochanek, S. J., Shun, T., Eiseman, J. L., & Johnston, P. A. (2018). Implementation of the NCI-60 Human Tumor Cell Line Panel to Screen 2260 Cancer Drug Combinations to Generate >3 Million Data Points Used to Populate a Large Matrix of Anti-Neoplastic Agent Combinations (ALMANAC) Database. *SLAS Discov*, 2472555218812429. doi:10.1177/2472555218812429

Clough, E., & Barrett, T. (2016). The Gene Expression Omnibus Database. In E. Mathé & S. Davis (Eds.), *Statistical Genomics: Methods and Protocols* (pp. 93-110). New York, NY: Springer New York.

Coker, E. A., Mitsopoulos, C., Tym, J. E., Komianou, A., Kannas, C., Di Micco, P., . . . Al-Lazikani, B. (2018). canSAR: update to the cancer translational research and drug discovery knowledgebase. *Nucleic Acids Res*. doi:10.1093/nar/gky1129

Consortium, G. T. (2013). The Genotype-Tissue Expression (GTEx) project. *Nature Genetics, 45*(6), 580-585. doi:10.1038/ng.2653

Conte, N., Mason, J. C., Halmagyi, C., Neuhauser, S., Mosaku, A., Yordanova, G., . . . Bult, C. C. (2018). PDX Finder: A portal for patient-derived tumor xenograft model discovery. *Nucleic Acids Research*, gky984-gky984. doi:10.1093/nar/gky984

Contreras, J. L., Floratos, A., & Holden, A. L. (2013). The International Serious Adverse Events Consortium's data sharing model. *Nat Biotechnol, 31*(1), 17-19. doi:10.1038/nbt.2470

Cook, K. B., Kazan, H., Zuberi, K., Morris, Q., & Hughes, T. R. (2011). RBPDB: a database of RNA-binding specificities. *Nucleic Acids Res, 39*(Database issue), D301-308. doi:10.1093/nar/gkq1069

Cooney, C., & Figg, B. (2018). A Review of the Household Products Database <hpd.nlm.nih.gov>. *Journal of Consumer Health on the Internet, 22*(2), 158-168. doi:10.1080/15398285.2018.1451145

Cordero, P., Lucks, J. B., & Das, R. (2012). An RNA Mapping DataBase for curating RNA structure mapping experiments. *Bioinformatics, 28*(22), 3006-3008. doi:10.1093/bioinformatics/bts554

Corsello, S. M., Bittker, J. A., Liu, Z., Gould, J., McCarren, P., Hirschman, J. E., . . . Golub, T. R. (2017). The Drug Repurposing Hub: a next-generation drug library and information resource. *Nature Medicine, 23*, 405. doi:10.1038/nm.4306

Cotter, D., Guda, P., Fahy, E., & Subramaniam, S. (2004). MitoProteome: mitochondrial protein sequence database and annotation system. *Nucleic Acids Res, 32*(Database issue), D463-467. doi:10.1093/nar/gkh048

Cotto, K. C., Wagner, A. H., Feng, Y.-Y., Kiwala, S., Coffman, A. C., Spies, G., . . . Griffith, M. (2018). DGIdb 3.0: a redesign and expansion of the drug–gene interaction database. *Nucleic Acids Research, 46*(D1), D1068-D1073. doi:10.1093/nar/gkx1143

Craigle, V. (2007). MedWatch: The FDA Safety Information and Adverse Event Reporting Program. *Journal of the Medical Library Association, 95*(2), 224-225. doi:10.3163/1536-5050.95.2.224

Crasto, C., Marenco, L., Miller, P., & Shepherd, G. (2002). Olfactory Receptor Database: a metadata-driven automated population from sources of gene and protein sequences. *Nucleic Acids Res, 30*(1), 354-360.

Crawford, K. L., Neu, S. C., & Toga, A. W. (2016). The Image and Data Archive at the Laboratory of Neuro Imaging. *NeuroImage, 124*(Pt B), 1080-1083. doi:10.1016/j.neuroimage.2015.04.067

Croft, D., O’Kelly, G., Wu, G., Haw, R., Gillespie, M., Matthews, L., . . . Stein, L. (2011). Reactome: a database of reactions, pathways and biological processes. *Nucleic Acids Research, 39*(Database issue), D691-D697. doi:10.1093/nar/gkq1018

Cronin MTD, M. J., Richarz A-N. (2012). THE COSMOS PROJECT: A FOUNDATION FOR THE FUTURE OF COMPUTATIONAL MODELLING OF REPEAT DOSE TOXICITY. *AltTox.Org*.

DA., F. (2007). Drug Interactions: Cytochrome P450 Drug Interaction Table. Retrieved from <https://drug-interactions.medicine.iu.edu>

Das, J., & Yu, H. (2012). HINT: High-quality protein interactomes and their applications in understanding human disease. *BMC Syst Biol, 6*, 92-92. doi:10.1186/1752-0509-6-92

Database Resources of the BIG Data Center in 2019. (2018). *Nucleic Acids Res*. doi:10.1093/nar/gky993

Davis, A. P., King, B. L., Mockus, S., Murphy, C. G., Saraceni-Richards, C., Rosenstein, M., . . . Mattingly, C. J. (2011). The Comparative Toxicogenomics Database: update 2011. *Nucleic Acids Res, 39*(Database issue), D1067-1072. doi:10.1093/nar/gkq813

Dawson, N. L., Lewis, T. E., Das, S., Lees, J. G., Lee, D., Ashford, P., . . . Sillitoe, I. (2017). CATH: an expanded resource to predict protein function through structure and sequence. *Nucleic Acids Research, 45*(D1), D289-D295. doi:10.1093/nar/gkw1098

De Baets, G., Van Durme, J., Reumers, J., Maurer-Stroh, S., Vanhee, P., Dopazo, J., . . . Rousseau, F. (2012). SNPeffect 4.0: on-line prediction of molecular and structural effects of protein-coding variants. *Nucleic Acids Research, 40*(Database issue), D935-D939. doi:10.1093/nar/gkr996

de Magalhaes, J. P., Costa, J., & Toussaint, O. (2005). HAGR: the Human Ageing Genomic Resources. *Nucleic Acids Res, 33*(Database issue), D537-543. doi:10.1093/nar/gki017

de Marcellus, S. (2014). *eChemPortal – The Global Portal to Information on Chemical Substances*.

de Matos, P., Cham, J. A., Cao, H., Alcántara, R., Rowland, F., Lopez, R., & Steinbeck, C. (2013). The Enzyme Portal: a case study in applying user-centred design methods in bioinformatics. *BMC Bioinformatics, 14*, 103. doi:10.1186/1471-2105-14-103

Dellaire, G., Farrall, R., & Bickmore, W. A. (2003). The Nuclear Protein Database (NPD): sub-nuclear localisation and functional annotation of the nuclear proteome. *Nucleic Acids Research, 31*(1), 328-330. doi:10.1093/nar/gkg018

Deng, Z., Tu, W., Deng, Z., & Hu, Q. N. (2017). PhID: An Open-Access Integrated Pharmacology Interactions Database for Drugs, Targets, Diseases, Genes, Side-Effects, and Pathways. *J Chem Inf Model, 57*(10), 2395-2400. doi:10.1021/acs.jcim.7b00175

Desaphy, J., Bret, G., Rognan, D., & Kellenberger, E. (2015). sc-PDB: a 3D-database of ligandable binding sites—10 years on. *Nucleic Acids Research, 43*(D1), D399-D404. doi:10.1093/nar/gku928

Deutsch, E. W. (2010). The PeptideAtlas Project. *Methods Mol Biol, 604*, 285-296. doi:10.1007/978-1-60761-444-9_19

Ding, D., Xu, L., Fang, H., Hong, H., Perkins, R., Harris, S., . . . Tong, W. (2010). The EDKB: an established knowledge base for endocrine disrupting chemicals. *BMC Bioinformatics, 11*(6), S5. doi:10.1186/1471-2105-11-s6-s5

Dingerdissen, H. M., Torcivia-Rodriguez, J., Hu, Y., Chang, T.-C., Mazumder, R., & Kahsay, R. (2018). BioMuta and BioXpress: mutation and expression knowledgebases for cancer biomarker discovery. *Nucleic Acids Research, 46*(D1), D1128-D1136. doi:10.1093/nar/gkx907

Dinkel, H., Chica, C., Via, A., Gould, C. M., Jensen, L. J., Gibson, T. J., & Diella, F. (2011). Phospho.ELM: a database of phosphorylation sites--update 2011. *Nucleic Acids Research, 39*(Database issue), D261-D267. doi:10.1093/nar/gkq1104

Dinman, J. D. (2005). 5S rRNA: Structure and Function from Head to Toe. *International journal of biomedical science : IJBS, 1*(1), 2-7.

Dong, J., Wang, N., Yao, Z., Zhang, L., Cheng, Y., Ouyang, D., . . . Cao, D.-S. (2018). *ADMETlab: a platform for systematic ADMET evaluation based on a comprehensively collected ADMET database* (Vol. 10).

Dong, Q., Han, H., Liu, X., Wei, L., Zhang, W., Zhao, Z., . . . Wang, X. (2017). HCSGD: An integrated database of human cellular senescence genes. *J Genet Genomics, 44*(5), 227-234. doi:10.1016/j.jgg.2017.04.001

Dourson, M. L. (2018). Let the IRIS Bloom:Regrowing the integrated risk information system (IRIS) of the U.S. Environmental Protection Agency. *Regulatory Toxicology and Pharmacology, 97*, A4-A5. doi:<https://doi.org/10.1016/j.yrtph.2018.05.003>

Dousse, A., Junier, T., & Zdobnov, E. M. (2016). CEGA--a catalog of conserved elements from genomic alignments. *Nucleic Acids Res, 44*(D1), D96-100. doi:10.1093/nar/gkv1163

Dreos, R., Ambrosini, G., Cavin Périer, R., & Bucher, P. (2013). EPD and EPDnew, high-quality promoter resources in the next-generation sequencing era. *Nucleic Acids Research, 41*(D1), D157-D164. doi:10.1093/nar/gks1233

Duan, G., Li, X., & Kohn, M. (2015). The human DEPhOsphorylation database DEPOD: a 2015 update. *Nucleic Acids Res, 43*(Database issue), D531-535. doi:10.1093/nar/gku1009

Dweep, H., & Gretz, N. (2015). miRWalk2.0: a comprehensive atlas of microRNA-target interactions. *Nat Methods, 12*(8), 697. doi:10.1038/nmeth.3485

Edgar, R., Mazor, Y., Rinon, A., Blumenthal, J., Golan, Y., Buzhor, E., . . . Shtrichman, R. (2013). LifeMap Discovery™: the embryonic development, stem cells, and regenerative medicine research portal. *PloS one, 8*(7), e66629-e66629. doi:10.1371/journal.pone.0066629

Elbourne, L. D. H., Tetu, S. G., Hassan, K. A., & Paulsen, I. T. (2017). TransportDB 2.0: a database for exploring membrane transporters in sequenced genomes from all domains of life. *Nucleic Acids Research, 45*(D1), D320-D324. doi:10.1093/nar/gkw1068

The ENCODE (ENCyclopedia Of DNA Elements) Project. (2004). *Science, 306*(5696), 636-640. doi:10.1126/science.1105136

Eppig, J. (2017). *Mouse Genome Informatics (MGI) Resource: Genetic, Genomic, and Biological Knowledgebase for the Laboratory Mouse* (Vol. 58).

Eppig, J. T., Motenko, H., Richardson, J. E., Richards-Smith, B., & Smith, C. L. (2015). The International Mouse Strain Resource (IMSR): cataloging worldwide mouse and ES cell line resources. *Mammalian genome : official journal of the International Mammalian Genome Society, 26*(9-10), 448-455. doi:10.1007/s00335-015-9600-0

Evelo, C. T., van Bochove, K., & Saito, J.-T. (2011). Answering biological questions: querying a systems biology database for nutrigenomics. *Genes & Nutrition, 6*(1), 81-87. doi:10.1007/s12263-010-0190-x

Fábién, P., & Degtyarenko, K. N. (1997). The Directory of P450-containing Systems in 1996. *Nucleic Acids Research, 25*(1), 274-277. doi:10.1093/nar/25.1.274

Fahy, E., Subramaniam, S., Murphy, R. C., Nishijima, M., Raetz, C. R. H., Shimizu, T., . . . Dennis, E. A. (2009). Update of the LIPID MAPS comprehensive classification system for lipids. *Journal of lipid research, 50 Suppl*(Suppl), S9-S14. doi:10.1194/jlr.R800095-JLR200

Fang, H., Harris, S. C., Su, Z., Chen, M., Qian, F., Shi, L., . . . Tong, W. (2017). ArrayTrack: An FDA and Public Genomic Tool. *Methods Mol Biol, 1613*, 333-353. doi:10.1007/978-1-4939-7027-8_13

Fang, H., Su, Z., Wang, Y., Miller, A., Liu, Z., Howard, P. C., . . . Lin, S. M. (2014). Exploring the FDA adverse event reporting system to generate hypotheses for monitoring of disease characteristics. *Clinical pharmacology and therapeutics, 95*(5), 496-498. doi:10.1038/clpt.2014.17

Fang, J., Wang, L., Li, Y., Lian, W., Pang, X., Wang, H., . . . Du, G.-H. (2017). AlzhCPI: A knowledge base for predicting chemical-protein interactions towards Alzheimer’s disease. *PloS one, 12*(5), e0178347. doi:10.1371/journal.pone.0178347

Farrell, C. M., O'Leary, N. A., Harte, R. A., Loveland, J. E., Wilming, L. G., Wallin, C., . . . Pruitt, K. D. (2014). Current status and new features of the Consensus Coding Sequence database. *Nucleic Acids Res, 42*(Database issue), D865-872. doi:10.1093/nar/gkt1059

Fazekas, D., Koltai, M., Turei, D., Modos, D., Palfy, M., Dul, Z., . . . Korcsmaros, T. (2013). SignaLink 2 - a signaling pathway resource with multi-layered regulatory networks. *BMC Syst Biol, 7*, 7. doi:10.1186/1752-0509-7-7

Feit, J., Kempf, W., Jedlickova, H., & Burg, G. (2005). Hypertext atlas of dermatopathology with expert system for epithelial tumors of the skin. *J Cutan Pathol, 32*(6), 433-437. doi:10.1111/j.0303-6987.2005.00291.x

Fenyo, D., & Beavis, R. C. (2015). The GPMDB REST interface. *Bioinformatics, 31*(12), 2056-2058. doi:10.1093/bioinformatics/btv107

Fernandes, M., & Husi, H. (2017). Establishment of a integrative multi-omics expression database CKDdb in the context of chronic kidney disease (CKD). *Scientific reports, 7*, 40367. doi:10.1038/srep40367

Fernández Escapa, I., Chen, T., Huang, Y., Gajare, P., Dewhirst, F. E., & Lemon, K. P. (2018). New insights into human nostril microbiome from the <em>expanded</em> Human Oral Microbiome Database (<em>e</em>HOMD): a resource for species-level identification of microbiome data from the aerodigestive tract. *bioRxiv*, 347013. doi:10.1101/347013

Ferrer, P., Ballarín, E., Sabaté, M., M, R., Hasford, J., Tatt, I., . . . behalf of the Protect project Wp, o. (2012). *Drug consumption databases in Europe*.

Fichant, G., Basse, M. J., & Quentin, Y. (2006). ABCdb: an online resource for ABC transporter repertories from sequenced archaeal and bacterial genomes. *FEMS Microbiol Lett, 256*(2), 333-339. doi:10.1111/j.1574-6968.2006.00139.x

Fiehn, O. (2016). Metabolomics by Gas Chromatography-Mass Spectrometry: Combined Targeted and Untargeted Profiling. *Current protocols in molecular biology, 114*, 30.34.31-30.34.32. doi:10.1002/0471142727.mb3004s114

Finn, R. D., Bateman, A., Clements, J., Coggill, P., Eberhardt, R. Y., Eddy, S. R., . . . Punta, M. (2014). Pfam: the protein families database. *Nucleic Acids Research, 42*(Database issue), D222-D230. doi:10.1093/nar/gkt1223

Fioravanzo, E., Yang, C., Rathman, J. F., Schwab, C., Kovarich, S., Ombrato, R., & Silvestri, L. (2015). *In silico assessment of genotoxic impurities: Case-studies using ChemTunes Studio* (Vol. 238).

Firth, H. V., Richards, S. M., Bevan, A. P., Clayton, S., Corpas, M., Rajan, D., . . . Carter, N. P. (2009). DECIPHER: Database of Chromosomal Imbalance and Phenotype in Humans Using Ensembl Resources. *Am J Hum Genet, 84*(4), 524-533. doi:10.1016/j.ajhg.2009.03.010

Fitzpatrick, R. B. (2007). LactMed. *Journal of Electronic Resources in Medical Libraries, 4*(1-2), 155-166. doi:10.1300/J383v04n01_14

Flamholz, A., Noor, E., Bar-Even, A., & Milo, R. (2012). eQuilibrator--the biochemical thermodynamics calculator. *Nucleic Acids Research, 40*(Database issue), D770-D775. doi:10.1093/nar/gkr874

Fleischmann, A., Darsow, M., Degtyarenko, K., Fleischmann, W., Boyce, S., Axelsen, K. B., . . . Apweiler, R. (2004). IntEnz, the integrated relational enzyme database. *Nucleic Acids Research, 32*(Database issue), D434-D437. doi:10.1093/nar/gkh119

Fonger, G. C. (1995). Hazardous substances data bank (HSDB) as a source of environmental fate information on chemicals. *Toxicology, 103*(2), 137-145.

Forbes, S. A., Bhamra, G., Bamford, S., Dawson, E., Kok, C., Clements, J., . . . Stratton, M. R. (2008). The Catalogue of Somatic Mutations in Cancer (COSMIC). *Current protocols in human genetics, Chapter 10*, Unit-10.11. doi:10.1002/0471142905.hg1011s57

Franco-Serrano, L., Hernandez, S., Calvo, A., Severi, M. A., Ferragut, G., Perez-Pons, J., . . . Cedano, J. (2018). MultitaskProtDB-II: an update of a database of multitasking/moonlighting proteins. *Nucleic Acids Res, 46*(D1), D645-d648. doi:10.1093/nar/gkx1066

Frankish, A., Diekhans, M., Ferreira, A.-M., Johnson, R., Jungreis, I., Loveland, J., . . . Flicek, P. (2018). GENCODE reference annotation for the human and mouse genomes. *Nucleic Acids Research*, gky955-gky955. doi:10.1093/nar/gky955

Freimuth, R. R., Wix, K., Zhu, Q., Siska, M., & Chute, C. G. (2014). Evaluation of RxNorm for Medication Clinical Decision Support. *AMIA ... Annual Symposium proceedings. AMIA Symposium, 2014*, 554-563.

Frezal, J. (1998). Genatlas database, genes and development defects. *C R Acad Sci III, 321*(10), 805-817.

Friar, J. H., & Vittori, W. D. (2017). The Pharos Project: Solving the Building Materials Toxicity Challenge. *Entrepreneurship Theory and Practice, 41*(1), 131-141. doi:doi:10.1111/etap.12170

Frolkis, A., Knox, C., Lim, E., Jewison, T., Law, V., Hau, D. D., . . . Wishart, D. S. (2010). SMPDB: The Small Molecule Pathway Database. *Nucleic Acids Research, 38*(Database issue), D480-D487. doi:10.1093/nar/gkp1002

Frye, S. V. (2010). The art of the chemical probe. *Nature chemical biology, 6*, 159. doi:10.1038/nchembio.296

Fujimori, S., Hirai, N., Masuoka, K., Oshikubo, T., Yamashita, T., Washio, T., . . . Miyamoto-Sato, E. (2012). IRView: a database and viewer for protein interacting regions. *Bioinformatics, 28*(14), 1949-1950. doi:10.1093/bioinformatics/bts289

Fuller, J. C., Martinez, M., Henrich, S., Stank, A., Richter, S., & Wade, R. C. (2015). LigDig: a web server for querying ligand-protein interactions. *Bioinformatics, 31*(7), 1147-1149. doi:10.1093/bioinformatics/btu784

Fusenig, N. E., Capes-Davis, A., Bianchini, F., Sundell, S., & Lichter, P. (2017). The need for a worldwide consensus for cell line authentication: Experience implementing a mandatory requirement at the International Journal of Cancer. *PLOS Biology, 15*(4), e2001438-e2001438. doi:10.1371/journal.pbio.2001438

Gabanyi, M. J., Adams, P. D., Arnold, K., Bordoli, L., Carter, L. G., Flippen-Andersen, J., . . . Berman, H. M. (2011). The Structural Biology Knowledgebase: a portal to protein structures, sequences, functions, and methods. *Journal of Structural and Functional Genomics, 12*(2), 45-54. doi:10.1007/s10969-011-9106-2

Gabrielson, S. W. (2018). SciFinder. *Journal of the Medical Library Association : JMLA, 106*(4), 588-590. doi:10.5195/jmla.2018.515

Gadaleta, E., Pirrò, S., Dayem Ullah, A. Z., Marzec, J., & Chelala, C. (2018). BCNTB bioinformatics: the next evolutionary step in the bioinformatics of breast cancer tissue banking. *Nucleic Acids Research, 46*(D1), D1055-D1061. doi:10.1093/nar/gkx913

Gadewal, N., & Zingde, S. (2011). *Database and interaction network of genes involved in oral cancer: Version II* (Vol. 6).

Gaedigk, A., Ingelman-Sundberg, M., Miller, N. A., Leeder, J. S., Whirl-Carrillo, M., & Klein, T. E. (2018). The Pharmacogene Variation (PharmVar) Consortium: Incorporation of the Human Cytochrome P450 (CYP) Allele Nomenclature Database. *Clin Pharmacol Ther, 103*(3), 399-401. doi:10.1002/cpt.910

Gaieb, Z., Liu, S., Gathiaka, S., Chiu, M., Yang, H., Shao, C., . . . Amaro, R. E. (2018). D3R Grand Challenge 2: blind prediction of protein–ligand poses, affinity rankings, and relative binding free energies. *Journal of Computer-Aided Molecular Design, 32*(1), 1-20. doi:10.1007/s10822-017-0088-4

Gallin, W. J., & Boutet, P. A. (2011). VKCDB: voltage-gated K + channel database updated and upgraded. *Nucleic Acids Research, 39*(suppl_1), D362-D366. doi:10.1093/nar/gkq1000

Gamazon, E. R., Duan, S., Zhang, W., Huang, R. S., Kistner, E. O., Dolan, M. E., & Cox, N. J. (2010). PACdb: a database for cell-based pharmacogenomics. *Pharmacogenet Genomics, 20*(4), 269-273. doi:10.1097/FPC.0b013e328337b8d6

Ganter, B., Snyder, R. D., Halbert, D. N., & Lee, M. D. (2006). Toxicogenomics in drug discovery and development: mechanistic analysis of compound/class-dependent effects using the DrugMatrix database. *Pharmacogenomics, 7*(7), 1025-1044. doi:10.2217/14622416.7.7.1025

Gao, J., Aksoy, B. A., Dogrusoz, U., Dresdner, G., Gross, B., Sumer, S. O., . . . Schultz, N. (2013). Integrative analysis of complex cancer genomics and clinical profiles using the cBioPortal. *Sci Signal, 6*(269), pl1. doi:10.1126/scisignal.2004088

Gao, J., Ellis, L. B. M., & Wackett, L. P. (2010). The University of Minnesota Biocatalysis/Biodegradation Database: improving public access. *Nucleic Acids Research, 38*(suppl_1), D488-D491. doi:10.1093/nar/gkp771

Gardiner, R. M. (2002). The Human Genome Project: the next decade. *86*(6), 389-391. doi:10.1136/adc.86.6.389 %J Archives of Disease in Childhood

Gaudet, P., Michel, P.-A., Zahn-Zabal, M., Britan, A., Cusin, I., Domagalski, M., . . . Bairoch, A. (2017). The neXtProt knowledgebase on human proteins: 2017 update. *Nucleic Acids Research, 45*(D1), D177-D182. doi:10.1093/nar/gkw1062

Gaulton, A., Hersey, A., Nowotka, M., Bento, A. P., Chambers, J., Mendez, D., . . . Leach, A. R. (2017). The ChEMBL database in 2017. *Nucleic Acids Research, 45*(D1), D945-D954. doi:10.1093/nar/gkw1074

Gazzo, A. M., Daneels, D., Cilia, E., Bonduelle, M., Abramowicz, M., Van Dooren, S., . . . Lenaerts, T. (2016). DIDA: A curated and annotated digenic diseases database. *Nucleic Acids Research, 44*(D1), D900-D907. doi:10.1093/nar/gkv1068

Geer, L. Y., Marchler-Bauer, A., Geer, R. C., Han, L., He, J., He, S., . . . Bryant, S. H. (2010). The NCBI BioSystems database. *Nucleic Acids Research, 38*(Database issue), D492-D496. doi:10.1093/nar/gkp858

Gene Ontology, C. (2015). Gene Ontology Consortium: going forward. *Nucleic Acids Research, 43*(Database issue), D1049-D1056. doi:10.1093/nar/gku1179

Gfeller, D., Michielin, O., & Zoete, V. (2012). *SwissSidechain: A molecular and structural database of non-natural sidechains* (Vol. 41).

Ghoorah, A. W., Devignes, M.-D., Smaïl-Tabbone, M., & Ritchie, D. W. (2014). KBDOCK 2013: a spatial classification of 3D protein domain family interactions. *Nucleic Acids Research, 42*(Database issue), D389-D395. doi:10.1093/nar/gkt1199

Gilson, M. K., Liu, T., Baitaluk, M., Nicola, G., Hwang, L., & Chong, J. (2016). BindingDB in 2015: A public database for medicinal chemistry, computational chemistry and systems pharmacology. *Nucleic Acids Res, 44*(D1), D1045-1053. doi:10.1093/nar/gkv1072

Giudicelli, V., Chaume, D., & Lefranc, M.-P. (2005). IMGT/GENE-DB: a comprehensive database for human and mouse immunoglobulin and T cell receptor genes. *Nucleic Acids Research, 33*(Database issue), D256-D261. doi:10.1093/nar/gki010

Gleeson, P., Cantarelli, M., Marin, B., Quintana, A., Earnshaw, M., Piasini, E., . . . Silver, R. A. (2018). Open Source Brain: a collaborative resource for visualizing, analyzing, simulating and developing standardized models of neurons and circuits. 229484. doi:10.1101/229484 %J bioRxiv

Gnad, F., Gunawardena, J., & Mann, M. (2011). PHOSIDA 2011: the posttranslational modification database. *Nucleic Acids Research, 39*(suppl_1), D253-D260. doi:10.1093/nar/gkq1159

Goede, A., Dunkel, M., Mester, N., Frommel, C., & Preissner, R. (2005). SuperDrug: a conformational drug database. *Bioinformatics, 21*(9), 1751-1753. doi:10.1093/bioinformatics/bti295

Gohlke, B. O., Nickel, J., Otto, R., Dunkel, M., & Preissner, R. (2016). CancerResource--updated database of cancer-relevant proteins, mutations and interacting drugs. *Nucleic Acids Res, 44*(D1), D932-937. doi:10.1093/nar/gkv1283

Gohlke, B. O., Preissner, R., & Preissner, S. (2014). SuperPain--a resource on pain-relieving compounds targeting ion channels. *Nucleic Acids Res, 42*(Database issue), D1107-1112. doi:10.1093/nar/gkt1176

Gold, L. S., Manley, N. B., Slone, T. H., & Rohrbach, L. (1999). Supplement to the Carcinogenic Potency Database (CPDB): results of animal bioassays published in the general literature in 1993 to 1994 and by the National Toxicology Program in 1995 to 1996. *Environ Health Perspect, 107 Suppl 4*, 527-600. doi:10.1289/ehp.99107s4527

Gonzalez-Galarza, F. F., Christmas, S., Middleton, D., & Jones, A. R. (2011). Allele frequency net: a database and online repository for immune gene frequencies in worldwide populations. *Nucleic Acids Research, 39*(suppl_1), D913-D919. doi:10.1093/nar/gkq1128

Gostev, M., Faulconbridge, A., Brandizi, M., Fernandez-Banet, J., Sarkans, U., Brazma, A., & Parkinson, H. (2012). The BioSample Database (BioSD) at the European Bioinformatics Institute. *Nucleic Acids Research, 40*(Database issue), D64-D70. doi:10.1093/nar/gkr937

Grazulis, S., Daskevic, A., Merkys, A., Chateigner, D., Lutterotti, L., Quiros, M., . . . Le Bail, A. (2012). Crystallography Open Database (COD): an open-access collection of crystal structures and platform for world-wide collaboration. *Nucleic Acids Res, 40*(Database issue), D420-427. doi:10.1093/nar/gkr900

Greshake, B., Bayer, P. E., Rausch, H., & Reda, J. (2014). openSNP--a crowdsourced web resource for personal genomics. *PloS one, 9*(3), e89204-e89204. doi:10.1371/journal.pone.0089204

Griffiths-Jones, S., Bateman, A., Marshall, M., Khanna, A., & Eddy, S. R. (2003). Rfam: an RNA family database. *Nucleic Acids Research, 31*(1), 439-441.

Groom, C. R., & Allen, F. H. (2014). The Cambridge Structural Database in retrospect and prospect. *Angew Chem Int Ed Engl, 53*(3), 662-671. doi:10.1002/anie.201306438

Grosdidier, A., Zoete, V., & Michielin, O. (2011). SwissDock, a protein-small molecule docking web service based on EADock DSS. *Nucleic Acids Research, 39*(suppl_2), W270-W277. doi:10.1093/nar/gkr366

Gstraunthaler, G., Lindl, T., & van der Valk, J. (2013). A plea to reduce or replace fetal bovine serum in cell culture media. *Cytotechnology, 65*(5), 791-793. doi:10.1007/s10616-013-9633-8

Günther, S., Hempel, D., Dunkel, M., Rother, K., & Preissner, R. (2007). SuperHapten: a comprehensive database for small immunogenic compounds. *Nucleic Acids Research, 35*(suppl_1), D906-D910. doi:10.1093/nar/gkl849

Günther, S., Kuhn, M., Dunkel, M., Campillos, M., Senger, C., Petsalaki, E., . . . Preissner, R. (2008). SuperTarget and Matador: resources for exploring drug-target relationships. *Nucleic Acids Research, 36*(Database issue), D919-D922. doi:10.1093/nar/gkm862

Guo, A.-Y., Webb, B. T., Miles, M. F., Zimmerman, M. P., Kendler, K. S., & Zhao, Z. (2009). ERGR: An ethanol-related gene resource. *Nucleic Acids Research, 37*(suppl_1), D840-D845. doi:10.1093/nar/gkn816

Guo, J., Liu, H., & Zheng, J. (2016). SynLethDB: synthetic lethality database toward discovery of selective and sensitive anticancer drug targets. *Nucleic Acids Research, 44*(D1), D1011-D1017. doi:10.1093/nar/gkv1108

Guo, L., Du, Y., Chang, S., Zhang, W., & Wang, J. (2014). Applying differentially expressed genes from rodent models of chronic stress to research of stress-related disease: an online database. *Psychosom Med, 76*(8), 644-649. doi:10.1097/psy.0000000000000102

Guo, L., Zhang, W., Chang, S., Zhang, L., Ott, J., & Wang, J. (2012). MK4MDD: a multi-level knowledge base and analysis platform for major depressive disorder. *PloS one, 7*(10), e46335. doi:10.1371/journal.pone.0046335

Gupta, A., Bug, W., Marenco, L., Qian, X., Condit, C., Rangarajan, A., . . . Martone, M. E. (2008). Federated Access to Heterogeneous Information Resources in the Neuroscience Information Framework (NIF). *Neuroinformatics, 6*(3), 205-217. doi:10.1007/s12021-008-9033-y

Gupta, A. B., Verma, R. K., Agarwal, V., Vajpai, M., Bansal, V., & Sankararamakrishnan, R. (2012). MIPModDB: a central resource for the superfamily of major intrinsic proteins. *Nucleic Acids Res, 40*(Database issue), D362-369. doi:10.1093/nar/gkr914

Gupta, R., Birch, H., Rapacki, K., Brunak, S., & Hansen, J. E. (1999). O-GLYCBASE version 4.0: a revised database of O-glycosylated proteins. *Nucleic Acids Res, 27*(1), 370-372.

Haas, J., Barbato, A., Behringer, D., Studer, G., Roth, S., Bertoni, M., . . . Schwede, T. (2018). Continuous Automated Model EvaluatiOn (CAMEO) complementing the critical assessment of structure prediction in CASP12. *86*(S1), 387-398. doi:doi:10.1002/prot.25431

Hachad, H., Overby, C. L., Argon, S., Yeung, C. K., Ragueneau-Majlessi, I., & Levy, R. H. (2011). e-PKGene: a knowledge-based research tool for analysing the impact of genetics on drug exposure. *Hum Genomics, 5*(5), 506-515.

Hachad, H., Ragueneau-Majlessi, I., & Levy, R. H. (2010). A useful tool for drug interaction evaluation: the University of Washington Metabolism and Transport Drug Interaction Database. *Hum Genomics, 5*(1), 61-72.

Hähnke, V. D., Kim, S., & Bolton, E. E. (2018). PubChem chemical structure standardization. *Journal of Cheminformatics, 10*(1), 36. doi:10.1186/s13321-018-0293-8

Hamosh, A., Scott, A. F., Amberger, J. S., Bocchini, C. A., & McKusick, V. A. (2005). Online Mendelian Inheritance in Man (OMIM), a knowledgebase of human genes and genetic disorders. *Nucleic Acids Research, 33*(suppl_1), D514-D517. doi:10.1093/nar/gki033

Hanzlik, R. P., Koen, Y. M., Theertham, B., Dong, Y., & Fang, J. (2007). The reactive metabolite target protein database (TPDB) – a web-accessible resource. *BMC Bioinformatics, 8*(1), 95. doi:10.1186/1471-2105-8-95

Hardt, C., Beber, M. E., Rasche, A., Kamburov, A., Hebels, D. G., Kleinjans, J. C., & Herwig, R. (2016). ToxDB: pathway-level interpretation of drug-treatment data. *Database, 2016*, baw052-baw052. doi:10.1093/database/baw052

Harrison, J., & Mozzicato, P. (2009). MedDRA®: The tale of a terminology: Side Effects of Drugs Essay. In J. K. Aronson (Ed.), *Side Effects of Drugs Annual* (Vol. 31, pp. xxxiii-xli): Elsevier.

Harrison, P. W., Alako, B., Amid, C., Cerdeño-Tárraga, A., Cleland, I., Holt, S., . . . Cochrane, G. (2018). The European Nucleotide Archive in 2018. *Nucleic Acids Research*, gky1078-gky1078. doi:10.1093/nar/gky1078

Hashemikhabir, S., Neelamraju, Y., & Janga, S. C. (2015). Database of RNA binding protein expression and disease dynamics (READ DB). *Database (Oxford), 2015*, bav072. doi:10.1093/database/bav072

Hastings, J., Owen, G., Dekker, A., Ennis, M., Kale, N., Muthukrishnan, V., . . . Steinbeck, C. (2015). *ChEBI in 2016: Improved services and an expanding collection of metabolites* (Vol. 44).

He, B., Chai, G., Duan, Y., Yan, Z., Qiu, L., Zhang, H., . . . Huang, J. (2016). BDB: biopanning data bank. *Nucleic Acids Res, 44*(D1), D1127-1132. doi:10.1093/nar/gkv1100

He, X., Chang, S., Zhang, J., Zhao, Q., Xiang, H., Kusonmano, K., . . . Wang, J. (2008). MethyCancer: the database of human DNA methylation and cancer. *Nucleic Acids Research, 36*(suppl_1), D836-D841. doi:10.1093/nar/gkm730

Hecker, N., Ahmed, J., von Eichborn, J., Dunkel, M., Macha, K., Eckert, A., . . . Preissner, R. (2012). SuperTarget goes quantitative: update on drug-target interactions. *Nucleic Acids Research, 40*(Database issue), D1113-D1117. doi:10.1093/nar/gkr912

Heidorn, C. J. A., Hansen, B. G., & Nørager, O. (1996). IUCLID:  A Database on Chemical Substances Information as a Tool for the EU-Risk-Assessment Program. *J Chem Inf Comput Sci, 36*(5), 949-954. doi:10.1021/ci9600014

Heifets, A., & Jurisica, I. (2012). SCRIPDB: a portal for easy access to syntheses, chemicals and reactions in patents. *Nucleic Acids Research, 40*(Database issue), D428-D433. doi:10.1093/nar/gkr919

Hendrickx, D., Aerts, H., Caiment, F., Clark, D., Ebbels, T., Evelo, C., . . . Kleinjans, J. (2014). *diXa: a Data Infrastructure for Chemical Safety Assessment* (Vol. 31).

Herrett, E., Gallagher, A. M., Bhaskaran, K., Forbes, H., Mathur, R., van Staa, T., & Smeeth, L. (2015). Data Resource Profile: Clinical Practice Research Datalink (CPRD). *Int J Epidemiol, 44*(3), 827-836. doi:10.1093/ije/dyv098

Hertz-Fowler, C., Peacock, C. S., Wood, V., Aslett, M., Kerhornou, A., Mooney, P., . . . Barrell, B. (2004). GeneDB: a resource for prokaryotic and eukaryotic organisms. *Nucleic Acids Research, 32*(Database issue), D339-D343. doi:10.1093/nar/gkh007

Higasa, K., Miyake, N., Yoshimura, J., Okamura, K., Niihori, T., Saitsu, H., . . . Matsuda, F. (2016). Human genetic variation database, a reference database of genetic variations in the Japanese population. *J Hum Genet, 61*(6), 547-553. doi:10.1038/jhg.2016.12

Higurashi, M., Ishida, T., & Kinoshita, K. (2009). PiSite: a database of protein interaction sites using multiple binding states in the PDB. *Nucleic Acids Res, 37*(Database issue), D360-364. doi:10.1093/nar/gkn659

Ho Sui, S. J., Begley, K., Reilly, D., Chapman, B., McGovern, R., Rocca-Sera, P., . . . Hide, W. (2012). The Stem Cell Discovery Engine: an integrated repository and analysis system for cancer stem cell comparisons. *Nucleic Acids Research, 40*(Database issue), D984-D991. doi:10.1093/nar/gkr1051

Hoffmann, M. F., Preissner, S. C., Nickel, J., Dunkel, M., Preissner, R., & Preissner, S. (2014). The Transformer database: biotransformation of xenobiotics. *Nucleic Acids Res, 42*(Database issue), D1113-1117. doi:10.1093/nar/gkt1246

Horaitis, O., & Cotton, R. G. (2004). The challenge of documenting mutation across the genome: the human genome variation society approach. *Hum Mutat, 23*(5), 447-452. doi:10.1002/humu.20038

Hornbeck, P. V., Zhang, B., Murray, B., Kornhauser, J. M., Latham, V., & Skrzypek, E. (2015). PhosphoSitePlus, 2014: mutations, PTMs and recalibrations. *Nucleic Acids Res, 43*(Database issue), D512-520. doi:10.1093/nar/gku1267

How to determine humane endpoints for research animals. (2015). *Lab Animal, 45*, 19. doi:10.1038/laban.908

Howe, E. A., de Souza, A., Lahr, D. L., Chatwin, S., Montgomery, P., Alexander, B. R., . . . Clemons, P. A. (2015). BioAssay Research Database (BARD): chemical biology and probe-development enabled by structured metadata and result types. *Nucleic Acids Res, 43*(Database issue), D1163-1170. doi:10.1093/nar/gku1244

Hu, H., Miao, Y.-R., Jia, L.-H., Yu, Q.-Y., Zhang, Q., & Guo, A.-Y. (2018). AnimalTFDB 3.0: a comprehensive resource for annotation and prediction of animal transcription factors. *Nucleic Acids Research*, gky822-gky822. doi:10.1093/nar/gky822

Huang, H., Arighi, C. N., Ross, K. E., Ren, J., Li, G., Chen, S.-C., . . . Wu, C. H. (2018). iPTMnet: an integrated resource for protein post-translational modification network discovery. *Nucleic Acids Research, 46*(D1), D542-D550. doi:10.1093/nar/gkx1104

Huang, K.-Y., Wu, H.-Y., Chen, Y.-J., Lu, C.-T., Su, M.-G., Hsieh, Y.-C., . . . Chen, Y.-J. (2014). RegPhos 2.0: an updated resource to explore protein kinase–substrate phosphorylation networks in mammals. *Database, 2014*, bau034-bau034. doi:10.1093/database/bau034

Huang, K. Y., Su, M. G., Kao, H. J., Hsieh, Y. C., Jhong, J. H., Cheng, K. H., . . . Lee, T. Y. (2016). dbPTM 2016: 10-year anniversary of a resource for post-translational modification of proteins. *Nucleic Acids Res, 44*(D1), D435-446. doi:10.1093/nar/gkv1240

Huang, P. J., Chiu, L. Y., Lee, C. C., Yeh, Y. M., Huang, K. Y., Chiu, C. H., & Tang, P. (2018). mSignatureDB: a database for deciphering mutational signatures in human cancers. *Nucleic Acids Res, 46*(D1), D964-d970. doi:10.1093/nar/gkx1133

Huang, R., Xia, M., Sakamuru, S., Zhao, J., Shahane, S. A., Attene-Ramos, M., . . . Simeonov, A. (2016). Modelling the Tox21 10 K chemical profiles for in vivo toxicity prediction and mechanism characterization. *Nature Communications, 7*, 10425. doi:10.1038/ncomms10425

Huang, W.-Y., Hsu, S.-D., Huang, H.-Y., Sun, Y.-M., Chou, C.-H., Weng, S.-L., & Huang, H.-D. (2015). MethHC: a database of DNA methylation and gene expression in human cancer. *Nucleic Acids Research, 43*(D1), D856-D861. doi:10.1093/nar/gku1151

Huang, Z., Jiang, H., Liu, X., Chen, Y., Wong, J., Wang, Q., . . . Zhang, J. (2012). HEMD: an integrated tool of human epigenetic enzymes and chemical modulators for therapeutics. *PloS one, 7*(6), e39917. doi:10.1371/journal.pone.0039917

Huttlin, E. L., Bruckner, R. J., Paulo, J. A., Cannon, J. R., Ting, L., Baltier, K., . . . Harper, J. W. (2017). Architecture of the human interactome defines protein communities and disease networks. *Nature, 545*(7655), 505-509. doi:10.1038/nature22366

Igarashi, Y., Nakatsu, N., Yamashita, T., Ono, A., Ohno, Y., Urushidani, T., & Yamada, H. (2015). Open TG-GATEs: a large-scale toxicogenomics database. *Nucleic Acids Res, 43*(Database issue), D921-927. doi:10.1093/nar/gku955

Irwin, J. J., Shoichet, B. K., Mysinger, M. M., Huang, N., Colizzi, F., Wassam, P., & Cao, Y. (2009). Automated docking screens: a feasibility study. *Journal of medicinal chemistry, 52*(18), 5712-5720. doi:10.1021/jm9006966

Ito, J.-i., Tabei, Y., Shimizu, K., Tsuda, K., & Tomii, K. (2011). *PoSSuM: A database of similar protein-ligand binding and putative pockets* (Vol. 40).

Ivanciuc, O., Schein, C. H., & Braun, W. (2003). SDAP: database and computational tools for allergenic proteins. *Nucleic Acids Res, 31*(1), 359-362.

Ives, C., Campia, I., Wang, R. L., Wittwehr, C., & Edwards, S. (2017). Creating a Structured AOP Knowledgebase via Ontology-Based Annotations. *Appl In Vitro Toxicol, 3*(4), 298-311. doi:10.1089/aivt.2017.0017

J. Nelson, S., Douglas Johnston, W., & L. Humphreys, B. (2001). *Relationships in Medical Subject Headings (MeSH)*.

Jamali, A. A., Ferdousi, R., Razzaghi, S., Li, J., Safdari, R., & Ebrahimie, E. (2016). DrugMiner: comparative analysis of machine learning algorithms for prediction of potential druggable proteins. *Drug Discovery Today, 21*(5), 718-724. doi:<https://doi.org/10.1016/j.drudis.2016.01.007>

Jankauskaite, J., Jimenez-Garcia, B., Dapkunas, J., Fernandez-Recio, J., & Moal, I. H. (2018). SKEMPI 2.0: An updated benchmark of changes in protein-protein binding energy, kinetics and thermodynamics upon mutation. *Bioinformatics*. doi:10.1093/bioinformatics/bty635

Jefferson, E. R., Walsh, T. P., Roberts, T. J., & Barton, G. J. (2007). SNAPPI-DB: a database and API of Structures, iNterfaces and Alignments for Protein-Protein Interactions. *Nucleic Acids Res, 35*(Database issue), D580-589. doi:10.1093/nar/gkl836

Jeliazkova, N., Chomenidis, C., Doganis, P., Fadeel, B., Grafstrom, R., Hardy, B., . . . Willighagen, E. (2015). The eNanoMapper database for nanomaterial safety information. *Beilstein journal of nanotechnology, 6*, 1609-1634. doi:10.3762/bjnano.6.165

Jemimah, S., Yugandhar, K., & Michael Gromiha, M. (2017). PROXiMATE: a database of mutant protein–protein complex thermodynamics and kinetics. *Bioinformatics, 33*(17), 2787-2788. doi:10.1093/bioinformatics/btx312

Jeong, J., Kim, G., Moon, C., Kim, H. J., Kim, T. H., & Jang, J. (2018). Pathogen Box screening for hit identification against Mycobacterium abscessus. *PloS one, 13*(4), e0195595. doi:10.1371/journal.pone.0195595

Ji, Z. L., Han, L. Y., Yap, C. W., Sun, L. Z., Chen, X., & Chen, Y. Z. (2003). Drug Adverse Reaction Target Database (DART). *Drug Safety, 26*(10), 685-690. doi:10.2165/00002018-200326100-00002

Jia, B., Raphenya, A. R., Alcock, B., Waglechner, N., Guo, P., Tsang, K. K., . . . McArthur, A. G. (2017). CARD 2017: expansion and model-centric curation of the comprehensive antibiotic resistance database. *Nucleic Acids Res, 45*(D1), D566-d573. doi:10.1093/nar/gkw1004

Jia, J., An, Z., Ming, Y., Guo, Y., Li, W., Li, X., . . . Shi, T. (2018). PedAM: a database for Pediatric Disease Annotation and Medicine. *Nucleic Acids Research, 46*(D1), D977-D983. doi:10.1093/nar/gkx1049

Jie, X., Lonnie, Z., Peter, S., & Stephen, W. S. (2004). Molecular Cytogenetics of Autism. *Current Genomics, 5*(4), 347-364. doi:<http://dx.doi.org/10.2174/1389202043349246>

Jones, P., & Cote, R. (2008). The PRIDE proteomics identifications database: data submission, query, and dataset comparison. *Methods Mol Biol, 484*, 287-303. doi:10.1007/978-1-59745-398-1_19

Jordan, R. A., El-Manzalawy, Y., Dobbs, D., & Honavar, V. (2012). Predicting protein-protein interface residues using local surface structural similarity. *BMC Bioinformatics, 13*, 41-41. doi:10.1186/1471-2105-13-41

Juan-Blanco, T., Duran-Frigola, M., & Aloy, P. (2015). IntSide: a web server for the chemical and biological examination of drug side effects. *Bioinformatics, 31*(4), 612-613. doi:10.1093/bioinformatics/btu688

Judson, R., Richard, A., Dix, D., Houck, K., Elloumi, F., Martin, M., . . . Wolf, M. (2008). *ACToR—Aggregated Computational Toxicology Resource* (Vol. 233).

Jühling, F., Mörl, M., Hartmann, R. K., Sprinzl, M., Stadler, P. F., & Pütz, J. (2009). tRNAdb 2009: compilation of tRNA sequences and tRNA genes. *Nucleic Acids Research, 37*(suppl_1), D159-D162. doi:10.1093/nar/gkn772

Jupp, S., Klein, J., Schanstra, J., & Stevens, R. (2011). Developing a kidney and urinary pathway knowledge base. *J Biomed Semantics, 2*(2), S7. doi:10.1186/2041-1480-2-s2-s7

Kalathur, R. K. R., Pinto, J. P., Hernández-Prieto, M. A., Machado, R. S. R., Almeida, D., Chaurasia, G., & Futschik, M. E. (2014). UniHI 7: an enhanced database for retrieval and interactive analysis of human molecular interaction networks. *Nucleic Acids Research, 42*(Database issue), D408-D414. doi:10.1093/nar/gkt1100

Kale, N. S., Haug, K., Conesa, P., Jayseelan, K., Moreno, P., Rocca-Serra, P., . . . Steinbeck, C. (2016). MetaboLights: An Open-Access Database Repository for Metabolomics Data. *Curr Protoc Bioinformatics, 53*, 14.13.11-18. doi:10.1002/0471250953.bi1413s53

Kalinina, O. V., Wichmann, O., Apic, G., & Russell, R. B. (2011). Combinations of protein-chemical complex structures reveal new targets for established drugs. *PLoS Comput Biol, 7*(5), e1002043. doi:10.1371/journal.pcbi.1002043

Kalinina, O. V., Wichmann, O., Apic, G., & Russell, R. B. (2012). ProtChemSI: a network of protein-chemical structural interactions. *Nucleic Acids Res, 40*(Database issue), D549-553. doi:10.1093/nar/gkr1049

Kamburov, A., Stelzl, U., Lehrach, H., & Herwig, R. (2013). The ConsensusPathDB interaction database: 2013 update. *Nucleic Acids Research, 41*(D1), D793-D800. doi:10.1093/nar/gks1055

Kamens, J. (2015). The Addgene repository: an international nonprofit plasmid and data resource. *Nucleic Acids Research, 43*(Database issue), D1152-D1157. doi:10.1093/nar/gku893

Kamp, H., Fabian, E., Groeters, S., Herold, M., Krennrich, G., Looser, R., . . . van Ravenzwaay, B. (2012). Application of in vivo metabolomics to preclinical/toxicological studies: case study on phenytoin-induced systemic toxicity. *Bioanalysis, 4*(18), 2291-2301. doi:10.4155/bio.12.214

Kanehisa, M., Sato, Y., Furumichi, M., Morishima, K., & Tanabe, M. (2018). New approach for understanding genome variations in KEGG. *Nucleic Acids Research*, gky962-gky962. doi:10.1093/nar/gky962

Kass-Hout, T. A., Xu, Z., Mohebbi, M., Nelsen, H., Baker, A., Levine, J., . . . Bright, R. A. (2016). OpenFDA: an innovative platform providing access to a wealth of FDA's publicly available data. *Journal of the American Medical Informatics Association : JAMIA, 23*(3), 596-600. doi:10.1093/jamia/ocv153

Kawashima, S., Pokarowski, P., Pokarowska, M., Kolinski, A., Katayama, T., & Kanehisa, M. (2008). AAindex: amino acid index database, progress report 2008. *Nucleic Acids Res, 36*(Database issue), D202-205. doi:10.1093/nar/gkm998

Keerthikumar, S., Chisanga, D., Ariyaratne, D., Al Saffar, H., Anand, S., Zhao, K., . . . Mathivanan, S. (2016). ExoCarta: A Web-Based Compendium of Exosomal Cargo. *Journal of Molecular Biology, 428*(4), 688-692. doi:<https://doi.org/10.1016/j.jmb.2015.09.019>

Keil, J. M., Qalieh, A., & Kwan, K. Y. (2018). Brain Transcriptome Databases: A User's Guide. *38*(10), 2399-2412. doi:10.1523/JNEUROSCI.1930-17.2018 %J The Journal of Neuroscience

Kellenberger, E., Muller, P., Schalon, C., Bret, G., Foata, N., & Rognan, D. (2006). sc-PDB: an annotated database of druggable binding sites from the Protein Data Bank. *J Chem Inf Model, 46*(2), 717-727. doi:10.1021/ci050372x

Kerrien, S., Aranda, B., Breuza, L., Bridge, A., Broackes-Carter, F., Chen, C., . . . Hermjakob, H. (2012). The IntAct molecular interaction database in 2012. *Nucleic Acids Research, 40*(Database issue), D841-D846. doi:10.1093/nar/gkr1088

Kersey, P. J., Allen, J. E., Allot, A., Barba, M., Boddu, S., Bolt, B. J., . . . Yates, A. (2018). Ensembl Genomes 2018: an integrated omics infrastructure for non-vertebrate species. *Nucleic Acids Research, 46*(D1), D802-D808. doi:10.1093/nar/gkx1011

Khan, A. N., Griffith, S. P., Moore, C., Russell, D., Rosario, A. C., Jr., & Bertolli, J. (2006). Standardizing laboratory data by mapping to LOINC. *Journal of the American Medical Informatics Association : JAMIA, 13*(3), 353-355. doi:10.1197/jamia.M1935

Khare, S. P., Habib, F., Sharma, R., Gadewal, N., Gupta, S., & Galande, S. (2012). HIstome--a relational knowledgebase of human histone proteins and histone modifying enzymes. *Nucleic Acids Res, 40*(Database issue), D337-342. doi:10.1093/nar/gkr1125

Kim, M. S., Pinto, S. M., Getnet, D., Nirujogi, R. S., Manda, S. S., Chaerkady, R., . . . Pandey, A. (2014). A draft map of the human proteome. *Nature, 509*(7502), 575-581. doi:10.1038/nature13302

Kim, P., Cheng, F., Zhao, J., & Zhao, Z. (2016). ccmGDB: a database for cancer cell metabolism genes. *Nucleic Acids Research, 44*(D1), D959-D968. doi:10.1093/nar/gkv1128

Kim, P., Park, A., Han, G., Sun, H., Jia, P., & Zhao, Z. (2018). TissGDB: tissue-specific gene database in cancer. *Nucleic Acids Research, 46*(D1), D1031-D1038. doi:10.1093/nar/gkx850

Kim, P., Zhao, J., Lu, P., & Zhao, Z. (2017). mutLBSgeneDB: mutated ligand binding site gene DataBase. *Nucleic Acids Research, 45*(D1), D256-D263. doi:10.1093/nar/gkw905

Kim, P., & Zhou, X. (2018). FusionGDB: fusion gene annotation DataBase. *Nucleic Acids Research*, gky1067-gky1067. doi:10.1093/nar/gky1067

Kim, Y., Ponomarenko, J., Zhu, Z., Tamang, D., Wang, P., Greenbaum, J., . . . Peters, B. (2012). Immune epitope database analysis resource. *Nucleic Acids Res, 40*(Web Server issue), W525-530. doi:10.1093/nar/gks438

King, Z. A., Lu, J., Dräger, A., Miller, P., Federowicz, S., Lerman, J. A., . . . Lewis, N. E. (2016). BiGG Models: A platform for integrating, standardizing and sharing genome-scale models. *Nucleic Acids Research, 44*(D1), D515-D522. doi:10.1093/nar/gkv1049

Kirsanov, D. D., Zanegina, O. N., Aksianov, E. A., Spirin, S. A., Karyagina, A. S., & Alexeevski, A. V. (2013). NPIDB: nucleic acid—protein interaction database. *Nucleic Acids Research, 41*(D1), D517-D523. doi:10.1093/nar/gks1199

Kodama, Y., Mashima, J., Kosuge, T., Katayama, T., Fujisawa, T., Kaminuma, E., . . . Nakamura, Y. (2015). The DDBJ Japanese Genotype-phenotype Archive for genetic and phenotypic human data. *Nucleic Acids Research, 43*(Database issue), D18-D22. doi:10.1093/nar/gku1120

Köhler, S., Carmody, L., Vasilevsky, N., Jacobsen, J. O B., Danis, D., Gourdine, J.-P., . . . Robinson, P. N. (2018). Expansion of the Human Phenotype Ontology (HPO) knowledge base and resources. *Nucleic Acids Research*, gky1105-gky1105. doi:10.1093/nar/gky1105

Kohonen, P., Benfenati, E., Bower, D., Ceder, R., Crump, M., Cross, K., . . . Hardy, B. (2013). The ToxBank Data Warehouse: Supporting the Replacement of In Vivo Repeated Dose Systemic Toxicity Testing. *32*(1), 47-63. doi:doi:10.1002/minf.201200114

Koleti, A., Terryn, R., Stathias, V., Chung, C., Cooper, D. J., Turner, J. P., . . . Schürer, S. C. (2018). Data Portal for the Library of Integrated Network-based Cellular Signatures (LINCS) program: integrated access to diverse large-scale cellular perturbation response data. *Nucleic Acids Research, 46*(D1), D558-D566. doi:10.1093/nar/gkx1063

Kolpakov, F., Poroikov, V., Sharipov, R., Kondrakhin, Y., Zakharov, A., Lagunin, A., . . . Kel, A. (2007). CYCLONET--an integrated database on cell cycle regulation and carcinogenesis. *Nucleic Acids Res, 35*(Database issue), D550-556. doi:10.1093/nar/gkl912

Kolte, B. S., Londhe, S. R., Solanki, B. R., Gacche, R. N., & Meshram, R. J. (2018). FilTer BaSe: A web accessible chemical database for small compound libraries. *Journal of Molecular Graphics and Modelling, 80*, 95-103. doi:<https://doi.org/10.1016/j.jmgm.2017.12.020>

Kooistra, A. J., Kanev, G. K., van Linden, O. P. J., Leurs, R., de Esch, I. J. P., & de Graaf, C. (2016). KLIFS: a structural kinase-ligand interaction database. *Nucleic Acids Research, 44*(D1), D365-D371. doi:10.1093/nar/gkv1082

Koscielny, G., An, P., Carvalho-Silva, D., Cham, J. A., Fumis, L., Gasparyan, R., . . . Dunham, I. (2017). Open Targets: a platform for therapeutic target identification and validation. *Nucleic Acids Research, 45*(D1), D985-D994. doi:10.1093/nar/gkw1055

Koscielny, G., Yaikhom, G., Iyer, V., Meehan, T. F., Morgan, H., Atienza-Herrero, J., . . . Parkinson, H. (2014). The International Mouse Phenotyping Consortium Web Portal, a unified point of access for knockout mice and related phenotyping data. *Nucleic Acids Research, 42*(Database issue), D802-D809. doi:10.1093/nar/gkt977

Kotlyar, M., Pastrello, C., Sheahan, N., & Jurisica, I. (2016). Integrated interactions database: tissue-specific view of the human and model organism interactomes. *Nucleic Acids Res, 44*(D1), D536-541. doi:10.1093/nar/gkv1115

Kowalski, P. J., Anderson, B. A., Moore, S., & Wilder, L. (2013). Spotlight on ATSDR: exposure investigations. *Journal of environmental health, 76*(5), 40-42.

Kozlowski, L. P. (2017). Proteome-pI: proteome isoelectric point database. *Nucleic Acids Res, 45*(D1), D1112-d1116. doi:10.1093/nar/gkw978

Kozma, D., Simon, I., & Tusnády, G. E. (2013). PDBTM: Protein Data Bank of transmembrane proteins after 8 years. *Nucleic Acids Research, 41*(Database issue), D524-D529. doi:10.1093/nar/gks1169

Kozomara, A., & Griffiths-Jones, S. (2014). miRBase: annotating high confidence microRNAs using deep sequencing data. *Nucleic Acids Research, 42*(D1), D68-D73. doi:10.1093/nar/gkt1181

Kriventseva, E. V., Kuznetsov, D., Tegenfeldt, F., Manni, M., Dias, R., Simao, F. A., & Zdobnov, E. M. (2018). OrthoDB v10: sampling the diversity of animal, plant, fungal, protist, bacterial and viral genomes for evolutionary and functional annotations of orthologs. *Nucleic Acids Res*. doi:10.1093/nar/gky1053

Kroetz, D. L., Yee, S. W., & Giacomini, K. M. (2009). The Pharmacogenomics of Membrane Transporters Project: Research at the Interface of Genomics and Transporter Pharmacology. *Clinical Pharmacology & Therapeutics, 87*(1), 109-116. doi:10.1038/clpt.2009.226

Krug, H. F., Bohmer, N., Kühnel, D., Marquardt, C., Nau, K., & Steinbach, C. (2018). The DaNa(2.0) Knowledge Base Nanomaterials-An Important Measure Accompanying Nanomaterials Development. *Nanomaterials (Basel, Switzerland), 8*(4), 204. doi:10.3390/nano8040204

Krupke, D. M., Begley, D. A., Sundberg, J. P., Richardson, J. E., Neuhauser, S. B., & Bult, C. J. (2017). The Mouse Tumor Biology Database: A Comprehensive Resource for Mouse Models of Human Cancer. *Cancer Research, 77*(21), e67-e70. doi:10.1158/0008-5472.Can-17-0584

Krupp, M., Itzel, T., Maass, T., Hildebrandt, A., Galle, P. R., & Teufel, A. (2013). CellLineNavigator: a workbench for cancer cell line analysis. *Nucleic Acids Research, 41*(D1), D942-D948. doi:10.1093/nar/gks1012

Kuang, X., Dhroso, A., Han, J. G., Shyu, C. R., & Korkin, D. (2016). DOMMINO 2.0: integrating structurally resolved protein-, RNA-, and DNA-mediated macromolecular interactions. *Database (Oxford), 2016*. doi:10.1093/database/bav114

Kuhn, M., Letunic, I., Jensen, L. J., & Bork, P. (2016). The SIDER database of drugs and side effects. *Nucleic Acids Research, 44*(D1), D1075-D1079. doi:10.1093/nar/gkv1075

Kuhn, M., von Mering, C., Campillos, M., Jensen, L. J., & Bork, P. (2008). STITCH: interaction networks of chemicals and proteins. *Nucleic Acids Research, 36*(Database issue), D684-D688. doi:10.1093/nar/gkm795

Kuhn, S., & Schlörer, N. E. (2015). Facilitating quality control for spectra assignments of small organic molecules: nmrshiftdb2 – a free in-house NMR database with integrated LIMS for academic service laboratories. *Magnetic Resonance in Chemistry, 53*(8), 582-589. doi:doi:10.1002/mrc.4263

Kuiken, C., Thurmond, J., Dimitrijevic, M., & Yoon, H. (2012). The LANL hemorrhagic fever virus database, a new platform for analyzing biothreat viruses. *Nucleic Acids Research, 40*(Database issue), D587-D592. doi:10.1093/nar/gkr898

Kulinowski, K. M., & Jaffe, M. P. (2009). *The GoodNanoGuide: A novel approach for developing good practices for handling engineered nanomaterials in an occupational setting* (Vol. 6).

Kumar, M. D., & Gromiha, M. M. (2006). PINT: Protein-protein Interactions Thermodynamic Database. *Nucleic Acids Res, 34*(Database issue), D195-198. doi:10.1093/nar/gkj017

Kumar, P., Han, B. C., Shi, Z., Jia, J., Wang, Y. P., Zhang, Y. T., . . . Chen, Y. Z. (2009). Update of KDBI: Kinetic Data of Bio-molecular Interaction database. *Nucleic Acids Res, 37*(Database issue), D636-641. doi:10.1093/nar/gkn839

Kumar, P., Mudunuri, S. B., Anaya, J., & Dutta, A. (2015). tRFdb: a database for transfer RNA fragments. *Nucleic Acids Res, 43*(Database issue), D141-145. doi:10.1093/nar/gku1138

Kumar, R., Chaudhary, K., Gupta, S., Singh, H., Kumar, S., Gautam, A., . . . Raghava, G. P. S. (2013). CancerDR: Cancer Drug Resistance Database. *Scientific reports, 3*, 1445. doi:10.1038/srep01445

Kumar, R., Chaudhary, K., Sharma, M., Nagpal, G., Chauhan, J. S., Singh, S., . . . Raghava, G. P. (2015). AHTPDB: a comprehensive platform for analysis and presentation of antihypertensive peptides. *Nucleic Acids Res, 43*(Database issue), D956-962. doi:10.1093/nar/gku1141

Kundrotas, P. J., Zhu, Z., & Vakser, I. A. (2012). GWIDD: a comprehensive resource for genome-wide structural modeling of protein-protein interactions. *Hum Genomics, 6*, 7. doi:10.1186/1479-7364-6-7

Kuppili Venkata, S., Repana, D., Nulsen, J., Dressler, L., Bortolomeazzi, M., Tourna, A., . . . Ciccarelli, F. D. (2018). The Network of Cancer Genes (NCG): a comprehensive catalogue of known and candidate cancer genes from cancer sequencing screens. *bioRxiv*, 389858. doi:10.1101/389858

Kurz, X., Perez-Gutthann, S., & Group, E. N. S. (2018). Strengthening standards, transparency, and collaboration to support medicine evaluation: Ten years of the European Network of Centres for Pharmacoepidemiology and Pharmacovigilance (ENCePP). *Pharmacoepidemiology and Drug Safety, 27*(3), 245-252. doi:10.1002/pds.4381

L Mitchell, A., Scheremetjew, M., Denise, H., Potter, S., Tarkowska, A., Qureshi, M., . . . Finn, R. (2017). *EBI Metagenomics in 2017: Enriching the analysis of microbial communities, from sequence reads to assemblies* (Vol. 46).

Langhorst, B. W., Jack, W. E., Reha-Krantz, L., & Nichols, N. M. (2012). Polbase: a repository of biochemical, genetic and structural information about DNA polymerases. *Nucleic Acids Research, 40*(Database issue), D381-D387. doi:10.1093/nar/gkr847

Lappalainen, I., Almeida-King, J., Kumanduri, V., Senf, A., Spalding, J. D., Ur-Rehman, S., . . . Flicek, P. (2015). The European Genome-phenome Archive of human data consented for biomedical research. *Nature Genetics, 47*(7), 692-695. doi:10.1038/ng.3312

Lappalainen, I., Lopez, J., Skipper, L., Hefferon, T., Spalding, D., Garner, J., . . . M Church, D. (2012). *DbVar and DGVa: Public archives for genomic structural variation* (Vol. 41).

Lappalainen, I., Lopez, J., Skipper, L., Hefferon, T., Spalding, J. D., Garner, J., . . . Church, D. M. (2013). DbVar and DGVa: public archives for genomic structural variation. *Nucleic Acids Res, 41*(Database issue), D936-941. doi:10.1093/nar/gks1213

Laskowski, R. A., Jabłońska, J., Pravda, L., Vařeková, R. S., & Thornton, J. M. (2018). PDBsum: Structural summaries of PDB entries. *Protein science : a publication of the Protein Society, 27*(1), 129-134. doi:10.1002/pro.3289

Lata, S., Bhasin, M., & Raghava, G. P. (2009). MHCBN 4.0: A database of MHC/TAP binding peptides and T-cell epitopes. *BMC Res Notes, 2*, 61. doi:10.1186/1756-0500-2-61

Le Novere, N., & Changeux, J. P. (2001). LGICdb: the ligand-gated ion channel database. *Nucleic Acids Res, 29*(1), 294-295.

Lea, I. A., Gong, H., Paleja, A., Rashid, A., & Fostel, J. (2017). CEBS: a comprehensive annotated database of toxicological data. *Nucleic Acids Research, 45*(D1), D964-D971. doi:10.1093/nar/gkw1077

Lee, D., Cornet, R., Lau, F., & de Keizer, N. (2013). A survey of SNOMED CT implementations. *Journal of Biomedical Informatics, 46*(1), 87-96. doi:<https://doi.org/10.1016/j.jbi.2012.09.006>

Lee, S., Zhang, C., Arif, M., Liu, Z., Benfeitas, R., Bidkhori, G., . . . Mardinoglu, A. (2018). TCSBN: a database of tissue and cancer specific biological networks. *Nucleic Acids Research, 46*(D1), D595-D600. doi:10.1093/nar/gkx994

Lefranc, M. P., Giudicelli, V., Kaas, Q., Duprat, E., Jabado-Michaloud, J., Scaviner, D., . . . Lefranc, G. (2005). IMGT, the international ImMunoGeneTics information system. *Nucleic Acids Res, 33*(Database issue), D593-597. doi:10.1093/nar/gki065

Legehar, A., Xhaard, H., & Ghemtio, L. (2016). *IDAAPM: Integrated database of ADMET and adverse effects of predictive modeling based on FDA approved drug data* (Vol. 8).

Lek, M., Karczewski, K. J., Minikel, E. V., Samocha, K. E., Banks, E., Fennell, T., . . . Exome Aggregation, C. (2016). Analysis of protein-coding genetic variation in 60,706 humans. *Nature, 536*, 285. doi:10.1038/nature19057

Lenoir, W. F., Lim, T. L., & Hart, T. (2018). PICKLES: the database of pooled in-vitro CRISPR knockout library essentiality screens. *Nucleic Acids Res, 46*(D1), D776-d780. doi:10.1093/nar/gkx993

Leroy, B., Ballinger, M. L., Baran-Marszak, F., Bond, G. L., Braithwaite, A., Concin, N., . . . Soussi, T. (2017). Recommended Guidelines for Validation, Quality Control, and Reporting of <em>TP53</em> Variants in Clinical Practice. *Cancer Research, 77*(6), 1250-1260. doi:10.1158/0008-5472.Can-16-2179

Lew, J. M., Kapopoulou, A., Jones, L. M., & Cole, S. T. (2011). TubercuList--10 years after. *Tuberculosis (Edinb), 91*(1), 1-7. doi:10.1016/j.tube.2010.09.008

Lexchin, J. (2014). Postmarket safety in Canada: are significant therapeutic advances and biologics less safe than other drugs? A cohort study. *BMJ Open, 4*(2), e004289-e004289. doi:10.1136/bmjopen-2013-004289

Li, J.-H., Liu, S., Zhou, H., Qu, L.-H., & Yang, J.-H. (2014). starBase v2.0: decoding miRNA-ceRNA, miRNA-ncRNA and protein–RNA interaction networks from large-scale CLIP-Seq data. *Nucleic Acids Research, 42*(D1), D92-D97. doi:10.1093/nar/gkt1248

Li, J., Shi, L., Zhang, K., Zhang, Y., Hu, S., Zhao, T., . . . Sun, Z. (2018). VarCards: an integrated genetic and clinical database for coding variants in the human genome. *Nucleic Acids Research, 46*(D1), D1039-D1048. doi:10.1093/nar/gkx1039

Li, P., Tompkins, R. G., Xiao, W., & the Inflammation and Host Response to Injury Large, Scale Collaborative Research P. (2017). KERIS: kaleidoscope of gene responses to inflammation between species. *Nucleic Acids Research, 45*(D1), D908-D914. doi:10.1093/nar/gkw974

Li, X., Shi, L., Zhang, K., Wei, W., Liu, Q., Mao, F., . . . Sun, Z. (2018). CirGRDB: a database for the genome-wide deciphering circadian genes and regulators. *Nucleic Acids Research, 46*(D1), D64-D70. doi:10.1093/nar/gkx944

Li, Y. H., Yu, C. Y., Li, X. X., Zhang, P., Tang, J., Yang, Q., . . . Zhu, F. (2018). Therapeutic target database update 2018: enriched resource for facilitating bench-to-clinic research of targeted therapeutics. *Nucleic Acids Res, 46*(D1), D1121-d1127. doi:10.1093/nar/gkx1076

Liberzon, A., Birger, C., Thorvaldsdóttir, H., Ghandi, M., Mesirov, Jill P., & Tamayo, P. (2015). The Molecular Signatures Database Hallmark Gene Set Collection. *Cell Systems, 1*(6), 417-425. doi:<https://doi.org/10.1016/j.cels.2015.12.004>

Licata, L., Briganti, L., Peluso, D., Perfetto, L., Iannuccelli, M., Galeota, E., . . . Cesareni, G. (2012). MINT, the molecular interaction database: 2012 update. *Nucleic Acids Res, 40*(Database issue), D857-861. doi:10.1093/nar/gkr930

Lilley, D. M., Clegg, R. M., Diekmann, S., Seeman, N. C., von Kitzing, E., & Hagerman, P. (1995). Nomenclature Committee of the International Union of Biochemistry and Molecular Biology (NC-IUBMB). A nomenclature of junctions and branchpoints in nucleic acids. Recommendations 1994. *Eur J Biochem, 230*(1), 1-2.

Lindquist, M. (2008). VigiBase, the WHO Global ICSR Database System: Basic Facts. *Drug Information Journal, 42*(5), 409-419. doi:10.1177/009286150804200501

Liu, B., & Pop, M. (2009). ARDB--Antibiotic Resistance Genes Database. *Nucleic Acids Research, 37*(Database issue), D443-D447. doi:10.1093/nar/gkn656

Liu, C., Bai, B., Skogerbø, G., Cai, L., Deng, W., Zhang, Y., . . . Chen, R. (2005). NONCODE: an integrated knowledge database of non-coding RNAs. *Nucleic Acids Research, 33*(Database issue), D112-D115. doi:10.1093/nar/gki041

Liu, X., Wang, S., Meng, F., Wang, J., Zhang, Y., Dai, E., . . . Jiang, W. (2013). SM2miR: a database of the experimentally validated small molecules' effects on microRNA expression. *Bioinformatics, 29*(3), 409-411. doi:10.1093/bioinformatics/bts698

Liu, X., Wang, S., Meng, F., Wang, J., Zhang, Y., Dai, E., . . . Jiang, W. (2013). SM2miR: a database of the experimentally validated small molecules’ effects on microRNA expression. *Bioinformatics, 29*(3), 409-411. doi:10.1093/bioinformatics/bts698

Liu, X., Yu, X., Zack, D. J., Zhu, H., & Qian, J. (2008). TiGER: A database for tissue-specific gene expression and regulation. *BMC Bioinformatics, 9*(1), 271. doi:10.1186/1471-2105-9-271

Liu, Y., Wei, Q., Yu, G., Gai, W., Li, Y., & Chen, X. (2014). DCDB 2.0: a major update of the drug combination database. *Database (Oxford), 2014*, bau124. doi:10.1093/database/bau124

Liu, Z., Li, Y., Han, L., Li, J., Liu, J., Zhao, Z., . . . Wang, R. (2015). PDB-wide collection of binding data: current status of the PDBbind database. *Bioinformatics, 31*(3), 405-412. doi:10.1093/bioinformatics/btu626

Ljosa, V., Sokolnicki, K. L., & Carpenter, A. E. (2012). Annotated high-throughput microscopy image sets for validation. *Nat Methods, 9*, 637. doi:10.1038/nmeth.2083

Lo Surdo, P., Calderone, A., Iannuccelli, M., Licata, L., Peluso, D., Castagnoli, L., . . . Perfetto, L. (2018). DISNOR: a disease network open resource. *Nucleic Acids Res, 46*(D1), D527-d534. doi:10.1093/nar/gkx876

Lomize, A. L., Hage, J. M., & Pogozheva, I. D. (2018). Membranome 2.0: database for proteome-wide profiling of bitopic proteins and their dimers. *Bioinformatics, 34*(6), 1061-1062. doi:10.1093/bioinformatics/btx720

López-Ibáñez, J., Pazos, F., & Chagoyen, M. (2016). MBROLE 2.0—functional enrichment of chemical compounds. *Nucleic Acids Research, 44*(W1), W201-W204. doi:10.1093/nar/gkw253

López, Y., Nakai, K., & Patil, A. (2015). HitPredict version 4: comprehensive reliability scoring of physical protein-protein interactions from more than 100 species. *Database (Oxford), 2015*, bav117. doi:10.1093/database/bav117

Los Alamos National, L., Theoretical, B., & Biophysics Group, T. (1995). HIV molecular immunology database. *HIV molecular immunology database.*

Lott, M. T., Leipzig, J. N., Derbeneva, O., Xie, H. M., Chalkia, D., Sarmady, M., . . . Wallace, D. C. (2013). mtDNA Variation and Analysis Using Mitomap and Mitomaster. *Curr Protoc Bioinformatics, 44*, 1.23.21-26. doi:10.1002/0471250953.bi0123s44

Lowe, H. J., Ferris, T. A., Hernandez, P. M., & Weber, S. C. (2009). STRIDE--An integrated standards-based translational research informatics platform. *AMIA ... Annual Symposium proceedings. AMIA Symposium, 2009*, 391-395.

Ludwig, C., Easton, J. M., Lodi, A., Tiziani, S., Manzoor, S. E., Southam, A. D., . . . Viant, M. R. (2012). Birmingham Metabolite Library: a publicly accessible database of 1-D 1H and 2-D 1H J-resolved NMR spectra of authentic metabolite standards (BML-NMR). *Metabolomics, 8*(1), 8-18. doi:10.1007/s11306-011-0347-7

Lüthi, A. U., & Martin, S. J. (2007). The CASBAH: a searchable database of caspase substrates. *Cell Death And Differentiation, 14*, 641. doi:10.1038/sj.cdd.4402103

Lv, J., Liu, H., Su, J., Wu, X., Liu, H., Li, B., . . . Zhang, Y. (2012). DiseaseMeth: a human disease methylation database. *Nucleic Acids Res, 40*(Database issue), D1030-1035. doi:10.1093/nar/gkr1169

Maccari, G., Robinson, J., Ballingall, K., Guethlein, L. A., Grimholt, U., Kaufman, J., . . . Marsh, S. G. (2017). IPD-MHC 2.0: an improved inter-species database for the study of the major histocompatibility complex. *Nucleic Acids Res, 45*(D1), D860-d864. doi:10.1093/nar/gkw1050

Madhavan, K., Zentner, L., Farnsworth, V., Shivarajapura, S., Zentner, M., Denny, N., & Klimeck, G. (2013). nanoHUB.org: cloud-based services for nanoscale modeling, simulation, and education. In *Nanotechnology Reviews* (Vol. 2, pp. 107).

Magarinos, M. P., Carmona, S. J., Crowther, G. J., Ralph, S. A., Roos, D. S., Shanmugam, D., . . . Aguero, F. (2012). TDR Targets: a chemogenomics resource for neglected diseases. *Nucleic Acids Res, 40*(Database issue), D1118-1127. doi:10.1093/nar/gkr1053

Maimon, O., & Browarnik, A. (2010). NHECD - Nano Health and Environmental Commented Database. In O. Maimon & L. Rokach (Eds.), *Data Mining and Knowledge Discovery Handbook* (pp. 1221-1241). Boston, MA: Springer US.

Major, S. M., Nishizuka, S., Morita, D., Rowland, R., Sunshine, M., Shankavaram, U., . . . Weinstein, J. N. (2006). AbMiner: A bioinformatic resource on available monoclonal antibodies and corresponding gene identifiers for genomic, proteomic, and immunologic studies. *BMC Bioinformatics, 7*(1), 192. doi:10.1186/1471-2105-7-192

Mak, L., Marcus, D., Howlett, A., Yarova, G., Duchateau, G., Klaffke, W., . . . Glen, R. C. (2015). Metrabase: a cheminformatics and bioinformatics database for small molecule transporter data analysis and (Q)SAR modeling. *Journal of Cheminformatics, 7*, 31. doi:10.1186/s13321-015-0083-5

Mangal, M., Sagar, P., Singh, H., Raghava, G. P. S., & Agarwal, S. M. (2013). NPACT: Naturally Occurring Plant-based Anti-cancer Compound-Activity-Target database. *Nucleic Acids Research, 41*(D1), D1124-D1129. doi:10.1093/nar/gks1047

Mannil, D., Vogt, I., Prinz, J., & Campillos, M. (2015). Organ system heterogeneity DB: a database for the visualization of phenotypes at the organ system level. *Nucleic Acids Res, 43*(Database issue), D900-906. doi:10.1093/nar/gku948

Marchant, C. A. (1996). Prediction of rodent carcinogenicity using the DEREK system for 30 chemicals currently being tested by the National Toxicology Program. The DEREK Collaborative Group. *Environ Health Perspect, 104 Suppl 5*(Suppl 5), 1065-1073. doi:10.1289/ehp.96104s51065

Marek, K., Chowdhury, S., Siderowf, A., Lasch, S., Coffey, C. S., Caspell-Garcia, C., . . . Sherer, T. (2018). The Parkinson's progression markers initiative (PPMI) – establishing a PD biomarker cohort. *5*(12), 1460-1477. doi:doi:10.1002/acn3.644

Marenco, L., Nadkarni, P., Skoufos, E., Shepherd, G., & Miller, P. (1999). Neuronal database integration: the Senselab EAV data model. *Proceedings. AMIA Symposium*, 102-106.

Margolis, R., Evans, R., & W O'Malley, B. (2005). *The Nuclear Receptor Signaling Atlas: Development of a Functional Atlas of Nuclear Receptors* (Vol. 19).

Mari, A., Mari, V., & Ronconi, A. (2005). Allergome—A database of allergenic molecules: Structure and data implementations of a web-based resource. *Journal of Allergy and Clinical Immunology, 115*(2, Supplement), S87. doi:<https://doi.org/10.1016/j.jaci.2004.12.359>

Marinelli, R. J., Montgomery, K., Liu, C. L., Shah, N. H., Prapong, W., Nitzberg, M., . . . Ball, C. A. (2008). The Stanford Tissue Microarray Database. *Nucleic Acids Research, 36*(Database issue), D871-D877. doi:10.1093/nar/gkm861

Martin, M. (2012). C-Path: Updating the Art of Pathology. *JNCI: Journal of the National Cancer Institute, 104*(16), 1202-1204. doi:10.1093/jnci/djs367

Martin, M. T., Mendez, E., Corum, D. G., Judson, R. S., Kavlock, R. J., Rotroff, D. M., & Dix, D. J. (2009). Profiling the reproductive toxicity of chemicals from multigeneration studies in the toxicity reference database. *Toxicol Sci, 110*(1), 181-190. doi:10.1093/toxsci/kfp080

Martinez, E., Moore, D. D., Keller, E., Pearce, D., Robinson, V., MacDonald, P. N., . . . Danielsen, M. (1997). The Nuclear Receptor Resource Project. *Nucleic Acids Res, 25*(1), 163-165.

Marzec, J., Dayem Ullah, A. Z., Pirro, S., Gadaleta, E., Crnogorac-Jurcevic, T., Lemoine, N. R., . . . Chelala, C. (2018). The Pancreatic Expression Database: 2018 update. *Nucleic Acids Res, 46*(D1), D1107-d1110. doi:10.1093/nar/gkx955

Mashima, J., Kodama, Y., Kosuge, T., Fujisawa, T., Katayama, T., Nagasaki, H., . . . Takagi, T. (2016). DNA data bank of Japan (DDBJ) progress report. *Nucleic Acids Research, 44*(D1), D51-D57. doi:10.1093/nar/gkv1105

Masson, P., Hulo, C., De Castro, E., Bitter, H., Gruenbaum, L., Essioux, L., . . . Le Mercier, P. (2013). ViralZone: recent updates to the virus knowledge resource. *Nucleic Acids Research, 41*(Database issue), D579-D583. doi:10.1093/nar/gks1220

Mathew, O. K., & Sowdhamini, R. (2016). PIMADb: A Database of Protein-Protein Interactions in Huge Macromolecular Assemblies. *Bioinform Biol Insights, 10*, 105-109. doi:10.4137/bbi.S38416

Mathias, S. L., Hines-Kay, J., Yang, J. J., Zahoransky-Kohalmi, G., Bologa, C. G., Ursu, O., & Oprea, T. I. (2013). The CARLSBAD Database: A Confederated Database of Chemical Bioactivities. *Database: The Journal of Biological Databases and Curation, 2013*, bat044. doi:10.1093/database/bat044

McCarthy, M., Hurt, D., Weber, N., Mtingwa, M., C Fincher, E., Alekseyev, V., . . . Swan, J. (2014). *The NIH 3D Print Exchange: A Public Resource for Bioscientific and Biomedical 3D Prints* (Vol. 1).

McDowall, M. D., Scott, M. S., & Barton, G. J. (2009). PIPs: human protein–protein interaction prediction database. *Nucleic Acids Research, 37*(suppl_1), D651-D656. doi:10.1093/nar/gkn870

Meldal, B. H M., Bye-A-Jee, H., Gajdoš, L., Hammerová, Z., Horáčková, A., Melicher, F., . . . Orchard, S. (2019). Complex Portal 2018: extended content and enhanced visualization tools for macromolecular complexes. *Nucleic Acids Research, 47*(D1), D550-D558. doi:10.1093/nar/gky1001

Melott, J. M., Weinstein, J. N., & Broom, B. M. (2016). PathwaysWeb: a gene pathways API with directional interactions, expanded gene ontology, and versioning. *Bioinformatics, 32*(2), 312-314. doi:10.1093/bioinformatics/btv554

Meng, X., Wang, J., Yuan, C., Li, X., Zhou, Y., Hofestädt, R., & Chen, M. (2015). CancerNet: a database for decoding multilevel molecular interactions across diverse cancer types. *Oncogenesis, 4*(12), e177-e177. doi:10.1038/oncsis.2015.40

Meyer, M. J., Das, J., Wang, X., & Yu, H. (2013). INstruct: a database of high-quality 3D structurally resolved protein interactome networks. *Bioinformatics, 29*(12), 1577-1579. doi:10.1093/bioinformatics/btt181

Mi, H., Muruganujan, A., Ebert, D., Huang, X., & Thomas, P. D. (2018). PANTHER version 14: more genomes, a new PANTHER GO-slim and improvements in enrichment analysis tools. *Nucleic Acids Res*. doi:10.1093/nar/gky1038

Miettinen, K., Inigo, S., Kreft, L., Pollier, J., De Bo, C., Botzki, A., . . . Goossens, A. (2018). The TriForC database: a comprehensive up-to-date resource of plant triterpene biosynthesis. *Nucleic Acids Res, 46*(D1), D586-d594. doi:10.1093/nar/gkx925

Milanesi, L., Petrillo, M., Sepe, L., Boccia, A., D'Agostino, N., Passamano, M., . . . Paolella, G. (2006). *Systematic analysis of human kinase genes: A large number of genes and alternative splicing events result in functional and structural diversity* (Vol. 6 Suppl 4).

Milanowska, K., Krwawicz, J., Papaj, G., Kosinski, J., Poleszak, K., Lesiak, J., . . . Bujnicki, J. M. (2011). REPAIRtoire--a database of DNA repair pathways. *Nucleic Acids Res, 39*(Database issue), D788-792. doi:10.1093/nar/gkq1087

Milne, G. W., Nicklaus, M. C., Driscoll, J. S., Wang, S., & Zaharevitz, D. (1994). National Cancer Institute Drug Information System 3D database. *J Chem Inf Comput Sci, 34*(5), 1219-1224.

Mitchell, A. L., Attwood, T. K., Babbitt, P. C., Blum, M., Bork, P., Bridge, A., . . . Finn, R. D. (2018). InterPro in 2019: improving coverage, classification and access to protein sequence annotations. *Nucleic Acids Res*. doi:10.1093/nar/gky1100

Mocellin, S., & Rossi, C. R. (2008). The Melanoma Molecular Map Project. *Melanoma Research, 18*(3), 163-165. doi:10.1097/CMR.0b013e328300c50b

Monga, M., & Sausville, E. A. (2002). Developmental therapeutics program at the NCI: molecular target and drug discovery process. *Leukemia, 16*(4), 520-526. doi:10.1038/sj.leu.2402464

Morawietz, G., Rittinghausen, S., & Mohr, U. (1992). RITA--Registry of Industrial Toxicology Animal-data--progress of the working group. *Exp Toxicol Pathol, 44*(6), 301-309. doi:10.1016/s0940-2993(11)80216-2

Morgat, A., Lombardot, T., Axelsen, K. B., Aimo, L., Niknejad, A., Hyka-Nouspikel, N., . . . Bridge, A. (2017). Updates in Rhea - an expert curated resource of biochemical reactions. *Nucleic Acids Res, 45*(D1), D415-d418. doi:10.1093/nar/gkw990

Morris, S. A., Gaheen, S., Lijowski, M., Heiskanen, M., & Klemm, J. (2015). Experiences in supporting the structured collection of cancer nanotechnology data using caNanoLab. *Beilstein journal of nanotechnology, 6*, 1580-1593. doi:10.3762/bjnano.6.161

Morrissey, K. M., Wen, C. C., Johns, S. J., Zhang, L., Huang, S. M., & Giacomini, K. M. (2012). The UCSF-FDA TransPortal: A Public Drug Transporter Database. *Clinical Pharmacology & Therapeutics, 92*(5), 545-546. doi:10.1038/clpt.2012.44

Mosca, R., Céol, A., Stein, A., Olivella, R., & Aloy, P. (2014). 3did: a catalog of domain-based interactions of known three-dimensional structure. *Nucleic Acids Research, 42*(D1), D374-D379. doi:10.1093/nar/gkt887

Moskalev, A., Zhikrivetskaya, S., Shaposhnikov, M., Dobrovolskaya, E., Gurinovich, R., Kuryan, O., . . . Zhavoronkov, A. (2016). Aging Chart: a community resource for rapid exploratory pathway analysis of age-related processes. *Nucleic Acids Research, 44*(D1), D894-D899. doi:10.1093/nar/gkv1287

Mottaz, A., David, F. P., Veuthey, A. L., & Yip, Y. L. (2010). Easy retrieval of single amino-acid polymorphisms and phenotype information using SwissVar. *Bioinformatics, 26*(6), 851-852. doi:10.1093/bioinformatics/btq028

Mudunuri, U., Che, A., Yi, M., & Stephens, R. M. (2009). bioDBnet: the biological database network. *Bioinformatics, 25*(4), 555-556. doi:10.1093/bioinformatics/btn654

Mukherjee, S., Stamatis, D., Bertsch, J., Ovchinnikova, G., Katta, H. Y., Mojica, A., . . . Reddy, T. B. K. (2018). Genomes OnLine database (GOLD) v.7: updates and new features. *Nucleic Acids Research*, gky977-gky977. doi:10.1093/nar/gky977

Müller, R., Weirick, T., John, D., Militello, G., Chen, W., Dimmeler, S., & Uchida, S. (2016). ANGIOGENES: knowledge database for protein-coding and noncoding RNA genes in endothelial cells. *Scientific reports, 6*, 32475. doi:10.1038/srep32475

Mungall, C. J., Torniai, C., Gkoutos, G. V., Lewis, S. E., & Haendel, M. A. J. G. B. (2012). Uberon, an integrative multi-species anatomy ontology. *13*(1), R5. doi:10.1186/gb-2012-13-1-r5

Murakami, Y., Omori, S., & Kinoshita, K. (2016). NLDB: a database for 3D protein–ligand interactions in enzymatic reactions. *Journal of Structural and Functional Genomics, 17*(4), 101-110. doi:10.1007/s10969-016-9206-0

Murugan, K., Sangeetha, S., Ranjitha, S., Vimala, A., Al-Sohaibani, S., & Rameshkumar, G. (2015). HDACiDB: a database for histone deacetylase inhibitors. *Drug design, development and therapy, 9*, 2257-2264. doi:10.2147/DDDT.S78276

Natale, D. A., Arighi, C. N., Barker, W. C., Blake, J. A., Bult, C. J., Caudy, M., . . . Wu, C. H. (2011). The Protein Ontology: a structured representation of protein forms and complexes. *Nucleic Acids Research, 39*(Database issue), D539-D545. doi:10.1093/nar/gkq907

Natsch, S., Hekster, Y. A., de Jong, R., Heerdink, E. R., Herings, R. M. C., van der Meer, J. W. M. J. E. J. o. C. M., & Diseases, I. (1998). Application of the ATC/DDD methodology to monitor antibiotic drug use. *17*(1), 20-24. doi:10.1007/bf01584358

Natsume-Kitatani, Y., Nyström-Persson, J., Igarashi, Y., Satoh, D., & Mizuguchi, K. (2017). Integrated toxicogenomics analysis with Toxygates for inferring molecular mechanisms. *Genomics and Computational Biology, 3*(1), e37. doi:10.18547/gcb.2017.vol3.iss1.e37

Nelson, J., Simpkins, S. W., Safizadeh, H., Li, S. C., Piotrowski, J. S., Hirano, H., . . . Myers, C. L. (2018). MOSAIC: a chemical-genetic interaction data repository and web resource for exploring chemical modes of action. *Bioinformatics, 34*(7), 1251-1252. doi:10.1093/bioinformatics/btx732

Neltner, T. G., Kulkarni, N. R., Alger, H. M., Maffini, M. V., Bongard, E. D., Fortin, N. D., & Olson, E. D. (2011). Navigating the U.S. Food Additive Regulatory Program. *Comprehensive Reviews in Food Science and Food Safety, 10*(6), 342-368. doi:doi:10.1111/j.1541-4337.2011.00166.x

Neveu, V., Moussy, A., Rouaix, H., Wedekind, R., Pon, A., Knox, C., . . . Scalbert, A. (2016). *Exposome-Explorer: A manually-curated database on biomarkers of exposure to dietary and environmental factors* (Vol. 45).

Newberg, J. Y., Mann, K. M., Mann, M. B., Jenkins, N. A., & Copeland, N. G. (2018). SBCDDB: Sleeping Beauty Cancer Driver Database for gene discovery in mouse models of human cancers. *Nucleic Acids Res, 46*(D1), D1011-d1017. doi:10.1093/nar/gkx956

Newburger, D. E., & Bulyk, M. L. (2009). UniPROBE: an online database of protein binding microarray data on protein-DNA interactions. *Nucleic Acids Res, 37*(Database issue), D77-82. doi:10.1093/nar/gkn660

Nguyen, D.-T., Mathias, S., Bologa, C., Brunak, S., Fernandez, N., Gaulton, A., . . . Guha, R. (2017). Pharos: Collating protein information to shed light on the druggable genome. *Nucleic Acids Research, 45*(D1), D995-D1002. doi:10.1093/nar/gkw1072

Nickel, J., Gohlke, B.-O., Erehman, J., Banerjee, P., Rong, W. W., Goede, A., . . . Preissner, R. (2014). SuperPred: update on drug classification and target prediction. *Nucleic Acids Research, 42*(Web Server issue), W26-W31. doi:10.1093/nar/gku477

Noguchi, H., Park, J., & Takagi, T. (2006). MetaGene: prokaryotic gene finding from environmental genome shotgun sequences. *Nucleic Acids Research, 34*(19), 5623-5630. doi:10.1093/nar/gkl723

Nusse, R., & Varmus, H. (2012). Three decades of Wnts: a personal perspective on how a scientific field developed. *Embo j, 31*(12), 2670-2684. doi:10.1038/emboj.2012.146

O'Mahony, C., Tozer, S., Davies, C., Dudzina, T., Finking, R., Meijster, T., . . . Zaleski, R. (2016). *ECETOC Technical Report no.126: Guidance for Effective Use of Human Exposure Data in Risk Assessment of Chemicals*.

O'Shea, K., Kattupalli, D., Mur, L. A., Hardy, N. W., Misra, B. B., & Lu, C. (2018). DIMEdb: an integrated database and web service for metabolite identification in direct infusion mass spectrometery. *bioRxiv*, 291799. doi:10.1101/291799

Oates, M. E., Romero, P., Ishida, T., Ghalwash, M., Mizianty, M. J., Xue, B., . . . Gough, J. (2013). D2P2: database of disordered protein predictions. *Nucleic Acids Research, 41*(D1), D508-D516. doi:10.1093/nar/gks1226

Oberbek, P. (2018). *NECID – nano exposure and contextual information database* (Vol. 35).

Ochoa-Montano, B., Mohan, N., & Blundell, T. L. (2015). CHOPIN: a web resource for the structural and functional proteome of Mycobacterium tuberculosis. *Database (Oxford), 2015*. doi:10.1093/database/bav026

Ohlmeier, C., Langner, I., Hillebrand, K., Schmedt, N., Mikolajczyk, R., Riedel, O., & Garbe, E. (2015). Mortality in the German Pharmacoepidemiological Research Database (GePaRD) compared to national data in Germany: results from a validation study. *BMC Public Health, 15*, 570. doi:10.1186/s12889-015-1943-7

Okuda, S., Nakao, H., & Kawasaki, T. (2021). GlycoEpitope: A Database for Carbohydrate Antigen and Antibody. In T. Endo, P. H. Seeberger, G. W. Hart, C.-H. Wong, & N. Taniguchi (Eds.), *Glycoscience: Biology and Medicine* (pp. 1-7). Tokyo: Springer Japan.

Okuno, Y., Yang, J., Taneishi, K., Yabuuchi, H., & Tsujimoto, G. (2006). GLIDA: GPCR-ligand database for chemical genomic drug discovery. *Nucleic Acids Research, 34*(suppl_1), D673-D677. doi:10.1093/nar/gkj028

Olender, T., Nativ, N., & Lancet, D. (2013). HORDE: Comprehensive Resource for Olfactory Receptor Genomics. In C. J. Crasto (Ed.), *Olfactory Receptors: Methods and Protocols* (pp. 23-38). Totowa, NJ: Humana Press.

Oliveira, F. S., Brestelli, J., Cade, S., Zheng, J., Iodice, J., Fischer, S., . . . Beiting, D. P. (2018). MicrobiomeDB: a systems biology platform for integrating, mining and analyzing microbiome experiments. *Nucleic Acids Research, 46*(D1), D684-D691. doi:10.1093/nar/gkx1027

Ong, E., Xiang, Z., Zhao, B., Liu, Y., Lin, Y., Zheng, J., . . . He, Y. (2017). Ontobee: A linked ontology data server to support ontology term dereferencing, linkage, query and integration. *Nucleic Acids Res, 45*(D1), D347-d352. doi:10.1093/nar/gkw918

Orchard, S., Kerrien, S., Abbani, S., Aranda, B., Bhate, J., Bidwell, S., . . . Hermjakob, H. (2012). Protein interaction data curation: the International Molecular Exchange (IMEx) consortium. *Nat Methods, 9*(4), 345-350. doi:10.1038/nmeth.1931

Orii, N., & Ganapathiraju, M. K. (2012). Wiki-Pi: A Web-Server of Annotated Human Protein-Protein Interactions to Aid in Discovery of Protein Function. *PloS one, 7*(11), e49029. doi:10.1371/journal.pone.0049029

Orloff, D. N., Iwasa, J. H., Martone, M. E., Ellisman, M. H., & Kane, C. M. (2013). The cell: an image library-CCDB: a curated repository of microscopy data. *Nucleic Acids Research, 41*(Database issue), D1241-D1250. doi:10.1093/nar/gks1257

Ortutay, C., Valiaho, J., Stenberg, K., & Vihinen, M. (2005). KinMutBase: a registry of disease-causing mutations in protein kinase domains. *Hum Mutat, 25*(5), 435-442. doi:10.1002/humu.20166

Oughtred, R., Stark, C., Breitkreutz, B.-J., Rust, J., Boucher, L., Chang, C., . . . Tyers, M. (2019). The BioGRID interaction database: 2019 update. *Nucleic Acids Research, 47*(D1), D529-D541. doi:10.1093/nar/gky1079

Ozawa, N., Shimizu, T., Morita, R., Yokono, Y., Ochiai, T., Munesada, K., . . . Sugiyama, Y. (2004). Transporter database, TP-Search: a web-accessible comprehensive database for research in pharmacokinetics of drugs. *Pharm Res, 21*(11), 2133-2134.

P. Cameron, T., M. Stump, J., & Schofield, L. (2019). *Chemical Carcinogenesis Research Information System (CCRIS) data bank, 1981June 1986 (1988 version). Data file*.

Paces, J., Pavlicek, A., & Paces, V. (2002). HERVd: database of human endogenous retroviruses. *Nucleic Acids Res, 30*(1), 205-206.

Pagel, P., Kovac, S., Oesterheld, M., Brauner, B., Dunger-Kaltenbach, I., Frishman, G., . . . Frishman, D. (2005). The MIPS mammalian protein–protein interaction database. *Bioinformatics, 21*(6), 832-834. doi:10.1093/bioinformatics/bti115

Paine, M. F., Shen, D. D., & McCune, J. S. (2018). Recommended Approaches for Pharmacokinetic Natural Product-Drug Interaction Research: a NaPDI Center Commentary. *46*(7), 1041-1045. doi:10.1124/dmd.117.079962 %J Drug Metabolism and Disposition

Paley, S., & Karp, P. D. (2017). Update notifications for the BioCyc collection of databases. *Database (Oxford), 2017*, bax086. doi:10.1093/database/bax086

Palmieri, V., Backes, C., Ludwig, N., Fehlmann, T., Kern, F., Meese, E., & Keller, A. (2018). IMOTA: an interactive multi-omics tissue atlas for the analysis of human miRNA-target interactions. *Nucleic Acids Research, 46*(D1), D770-D775. doi:10.1093/nar/gkx701

Pandurangan, A. P., Stahlhacke, J., Oates, M. E., Smithers, B., & Gough, J. (2019). The SUPERFAMILY 2.0 database: a significant proteome update and a new webserver. *Nucleic Acids Research, 47*(D1), D490-D494. doi:10.1093/nar/gky1130

Pándy-Szekeres, G., Munk, C., Tsonkov, T. M., Mordalski, S., Harpsøe, K., Hauser, A. S., . . . Gloriam, D. E. (2018). GPCRdb in 2018: adding GPCR structure models and ligands. *Nucleic Acids Research, 46*(D1), D440-D446. doi:10.1093/nar/gkx1109

Papadatos, G., Davies, M., Dedman, N., Chambers, J., Gaulton, A., Siddle, J., . . . Overington, J. P. (2016). SureChEMBL: a large-scale, chemically annotated patent document database. *Nucleic Acids Research, 44*(D1), D1220-D1228. doi:10.1093/nar/gkv1253

Papadopoulos, P., Viennas, E., Gkantouna, V., Pavlidis, C., Bartsakoulia, M., Ioannou, Z. M., . . . Patrinos, G. P. (2014). Developments in FINDbase worldwide database for clinically relevant genomic variation allele frequencies. *Nucleic Acids Res, 42*(Database issue), D1020-1026. doi:10.1093/nar/gkt1125

Papadopoulos, T., Krochmal, M., Cisek, K., Fernandes, M., Husi, H., Stevens, R., . . . Klein, J. (2016). Omics databases on kidney disease: where they can be found and how to benefit from them. *Clinical Kidney Journal, 9*(3), 343-352. doi:10.1093/ckj/sfv155

Papatheodorou, I., Fonseca, N. A., Keays, M., Tang, Y A., Barrera, E., Bazant, W., . . . Petryszak, R. (2018). Expression Atlas: gene and protein expression across multiple studies and organisms. *Nucleic Acids Research, 46*(D1), D246-D251. doi:10.1093/nar/gkx1158

Patterson, S., Statz, C., Yin, T., & Mockus, S. (2017). The JAX Clinical Knowledgebase: A Valuable Resource for Identifying Evidence Related to Complex Molecular Signatures in Different Types of Cancer. *Cancer Genetics, 214*, 33. doi:10.1016/j.cancergen.2017.04.006

Patwardhan, A. (2017). Trends in the Electron Microscopy Data Bank (EMDB). *Acta crystallographica. Section D, Structural biology, 73*(Pt 6), 503-508. doi:10.1107/S2059798317004181

Pavan, M., & Worth, A. P. (2008). Publicly-accessible QSAR software tools developed by the Joint Research Centre. *SAR QSAR Environ Res, 19*(7-8), 785-799. doi:10.1080/10629360802550390

Pavan, S., Rommel, K., Mateo Marquina, M. E., Höhn, S., Lanneau, V., & Rath, A. (2017). Clinical Practice Guidelines for Rare Diseases: The Orphanet Database. *PloS one, 12*(1), e0170365-e0170365. doi:10.1371/journal.pone.0170365

Pearce, N., Blair, A., Vineis, P., Ahrens, W., Andersen, A., Anto, J. M., . . . Zahm, S. H. (2015). IARC monographs: 40 years of evaluating carcinogenic hazards to humans. *Environ Health Perspect, 123*(6), 507-514. doi:10.1289/ehp.1409149

Pedersen, E., & Bongo, L. A. (2017). Large-scale biological meta-database management. *Future Generation Computer Systems, 67*, 481-489. doi:<https://doi.org/10.1016/j.future.2016.02.010>

Pedruzzi, I., Rivoire, C., Auchincloss, A. H., Coudert, E., Keller, G., de Castro, E., . . . Bridge, A. (2015). HAMAP in 2015: updates to the protein family classification and annotation system. *Nucleic Acids Research, 43*(Database issue), D1064-D1070. doi:10.1093/nar/gku1002

Pence, H. E., & Williams, A. (2010). ChemSpider: An Online Chemical Information Resource. *Journal of Chemical Education, 87*(11), 1123-1124. doi:10.1021/ed100697w

Perez-Riverol, Y., Bai, M., da Veiga Leprevost, F., Squizzato, S., Park, Y. M., Haug, K., . . . Hermjakob, H. (2017). Discovering and linking public omics data sets using the Omics Discovery Index. *Nature Biotechnology, 35*(5), 406-409. doi:10.1038/nbt.3790

Perfetto, L., Briganti, L., Calderone, A., Cerquone Perpetuini, A., Iannuccelli, M., Langone, F., . . . Cesareni, G. (2016). SIGNOR: a database of causal relationships between biological entities. *Nucleic Acids Res, 44*(D1), D548-554. doi:10.1093/nar/gkv1048

Peri, S., Navarro, J. D., Amanchy, R., Kristiansen, T. Z., Jonnalagadda, C. K., Surendranath, V., . . . Pandey, A. (2003). Development of human protein reference database as an initial platform for approaching systems biology in humans. *Genome Res, 13*(10), 2363-2371. doi:10.1101/gr.1680803

Peri, S., Navarro, J. D., Kristiansen, T. Z., Amanchy, R., Surendranath, V., Muthusamy, B., . . . Pandey, A. (2004). Human protein reference database as a discovery resource for proteomics. *Nucleic Acids Research, 32*(Database issue), D497-D501. doi:10.1093/nar/gkh070

Petretti, C., & Prigent, C. (2005). The Protein Kinase Resource: everything you always wanted to know about protein kinases but were afraid to ask. *Biol Cell, 97*(2), 113-118. doi:10.1042/bc20040077

Pickett, B. E., Sadat, E. L., Zhang, Y., Noronha, J. M., Squires, R. B., Hunt, V., . . . Scheuermann, R. H. (2012). ViPR: an open bioinformatics database and analysis resource for virology research. *Nucleic Acids Research, 40*(Database issue), D593-D598. doi:10.1093/nar/gkr859

Pietraszkiewicz, E., Firlag-Burkacka, E., Horban, A., & Kowalska, J. D. (2014). The suspected unexpected and serious adverse events of antiretroviral drugs used as HIV prophylaxis in HIV uninfected persons. *J Int AIDS Soc, 17*(4 Suppl 3), 19733. doi:10.7448/ias.17.4.19733

Pihan, E., Colliandre, L., Guichou, J. F., & Douguet, D. (2012). e-Drug3D: 3D structure collections dedicated to drug repurposing and fragment-based drug design. *Bioinformatics, 28*(11), 1540-1541. doi:10.1093/bioinformatics/bts186

Piñeiro-Yáñez, E., Reboiro-Jato, M., Gómez-López, G., Perales-Patón, J., Troulé, K., Rodríguez, J. M., . . . Al-Shahrour, F. (2018). PanDrugs: a novel method to prioritize anticancer drug treatments according to individual genomic data. *Genome Medicine, 10*(1), 41. doi:10.1186/s13073-018-0546-1

Piñero, J., Bravo, À., Queralt-Rosinach, N., Gutiérrez-Sacristán, A., Deu-Pons, J., Centeno, E., . . . Furlong, L. I. (2017). DisGeNET: a comprehensive platform integrating information on human disease-associated genes and variants. *Nucleic Acids Research, 45*(D1), D833-D839. doi:10.1093/nar/gkw943

Piovesan, D., Tabaro, F., Micetic, I., Necci, M., Quaglia, F., Oldfield, C. J., . . . Tosatto, S. C. (2017). DisProt 7.0: a major update of the database of disordered proteins. *Nucleic Acids Res, 45*(D1), D219-d227. doi:10.1093/nar/gkw1056

Piovesan, D., Tabaro, F., Paladin, L., Necci, M., Mičetić, I., Camilloni, C., . . . Tosatto, S. C. E. (2018). MobiDB 3.0: more annotations for intrinsic disorder, conformational diversity and interactions in proteins. *Nucleic Acids Research, 46*(D1), D471-D476. doi:10.1093/nar/gkx1071

Pires, D. E., Blundell, T. L., & Ascher, D. B. (2015). Platinum: a database of experimentally measured effects of mutations on structurally defined protein-ligand complexes. *Nucleic Acids Res, 43*(Database issue), D387-391. doi:10.1093/nar/gku966

Pirtskhalava, M., Gabrielian, A., Cruz, P., Griggs, H. L., Squires, R. B., Hurt, D. E., . . . Tartakovsky, M. (2016). DBAASP v.2: an enhanced database of structure and antimicrobial/cytotoxic activity of natural and synthetic peptides. *Nucleic Acids Res, 44*(D1), D1104-1112. doi:10.1093/nar/gkv1174

Placzek, S., Schomburg, I., Chang, A., Jeske, L., Ulbrich, M., Tillack, J., & Schomburg, D. (2017). BRENDA in 2017: new perspectives and new tools in BRENDA. *Nucleic Acids Res, 45*(D1), D380-d388. doi:10.1093/nar/gkw952

Plazzer, J. P., Sijmons, R. H., Woods, M. O., Peltomaki, P., Thompson, B., Den Dunnen, J. T., & Macrae, F. (2013). The InSiGHT database: utilizing 100 years of insights into Lynch syndrome. *Fam Cancer, 12*(2), 175-180. doi:10.1007/s10689-013-9616-0

Podlevsky, J. D., Bley, C. J., Omana, R. V., Qi, X., & Chen, J. J. (2008). The telomerase database. *Nucleic Acids Res, 36*(Database issue), D339-343. doi:10.1093/nar/gkm700

Poiron, C., Wu, Y., Ginestoux, C., Ehrenmann, F., Duroux, P., & Lefranc, M.-P. (2010). *IMGT/mAb-DB: the basis of IMGT data of therapeutic monoclonal antibodies* (Vol. 97).

Polak, S., Wiśniowska, B., Glinka, A., & Polak, M. (2012). Tox-database.net: a curated resource for data describing chemical triggered in vitro cardiac ion channels inhibition. *BMC pharmacology & toxicology, 13*, 6-6. doi:10.1186/2050-6511-13-6

Pornputtapong, N., Nookaew, I., & Nielsen, J. (2015). Human metabolic atlas: an online resource for human metabolism. *Database, 2015*, bav068-bav068. doi:10.1093/database/bav068

Postigo, R., Brosch, S., Slattery, J., van Haren, A., Dogne, J. M., Kurz, X., . . . Arlett, P. (2018). EudraVigilance Medicines Safety Database: Publicly Accessible Data for Research and Public Health Protection. *Drug Saf, 41*(7), 665-675. doi:10.1007/s40264-018-0647-1

Potapov, A., Liebich, I., Dönitz, J., Schwarzer, K., Sasse, N., Schoeps, T., . . . Wingender, E. (2006). EndoNet: an information resource about endocrine networks. *Nucleic Acids Research, 34*(Database issue), D540-D545. doi:10.1093/nar/gkj121

Pradeep, P., Povinelli, R. J., White, S., & Merrill, S. J. (2016). An ensemble model of QSAR tools for regulatory risk assessment. *J Cheminform, 8*, 48. doi:10.1186/s13321-016-0164-0

Prior, F., Smith, K., Sharma, A., Kirby, J., Tarbox, L., Clark, K., . . . Freymann, J. (2017). The public cancer radiology imaging collections of The Cancer Imaging Archive. *Scientific Data, 4*, 170124. doi:10.1038/sdata.2017.124

Prokai, L., Zharikova, A. D., & Stevens, S. M., Jr. (2005). Effect of chronic morphine exposure on the synaptic plasma-membrane subproteome of rats: a quantitative protein profiling study based on isotope-coded affinity tags and liquid chromatography/mass spectrometry. *J Mass Spectrom, 40*(2), 169-175. doi:10.1002/jms.736

Pruitt, K. D., Tatusova, T., & Maglott, D. R. (2007). NCBI reference sequences (RefSeq): a curated non-redundant sequence database of genomes, transcripts and proteins. *Nucleic Acids Research, 35*(Database issue), D61-D65. doi:10.1093/nar/gkl842

Pugalenthi, G., Bhaduri, A., & Sowdhamini, R. (2006). iMOTdb--a comprehensive collection of spatially interacting motifs in proteins. *Nucleic Acids Res, 34*(Database issue), D285-286. doi:10.1093/nar/gkj125

Putignano, V., Rosato, A., Banci, L., & Andreini, C. (2018). MetalPDB in 2018: a database of metal sites in biological macromolecular structures. *Nucleic Acids Res, 46*(D1), D459-d464. doi:10.1093/nar/gkx989

Qureshi, A., Thakur, N., Tandon, H., & Kumar, M. (2014). AVPdb: a database of experimentally validated antiviral peptides targeting medically important viruses. *Nucleic Acids Research, 42*(D1), D1147-D1153. doi:10.1093/nar/gkt1191

Rahmati, S., Abovsky, M., Pastrello, C., & Jurisica, I. (2017). pathDIP: an annotated resource for known and predicted human gene-pathway associations and pathway enrichment analysis. *Nucleic Acids Research, 45*(D1), D419-D426. doi:10.1093/nar/gkw1082

Raies, A. B., Mansour, H., Incitti, R., & Bajic, V. B. (2015). DDMGD: the database of text-mined associations between genes methylated in diseases from different species. *Nucleic Acids Research, 43*(D1), D879-D886. doi:10.1093/nar/gku1168

Rainville, I., & Garber, J. E. (2008). Familial Cancer Database Online. *The Lancet Oncology, 9*(10), 925-926. doi:<https://doi.org/10.1016/S1470-2045(08)70251-1>

Rajeevan, H., Cheung, K.-H., Gadagkar, R., Stein, S., Soundararajan, U., Kidd, J. R., . . . Kidd, K. K. (2005). ALFRED: An Allele Frequency Database for Microevolutionary Studies. *1*, 117693430500100006. doi:10.1177/117693430500100006

Rajput, A., Thakur, A., Sharma, S., & Kumar, M. (2018). aBiofilm: a resource of anti-biofilm agents and their potential implications in targeting antibiotic drug resistance. *Nucleic Acids Res, 46*(D1), D894-d900. doi:10.1093/nar/gkx1157

Rallapalli, P. M., Kemball-Cook, G., Tuddenham, E. G., Gomez, K., & Perkins, S. J. (2013). An interactive mutation database for human coagulation factor IX provides novel insights into the phenotypes and genetics of hemophilia B. *J Thromb Haemost, 11*(7), 1329-1340. doi:10.1111/jth.12276

Rappaport, N., Twik, M., Plaschkes, I., Nudel, R., Iny Stein, T., Levitt, J., . . . Lancet, D. (2017). MalaCards: an amalgamated human disease compendium with diverse clinical and genetic annotation and structured search. *Nucleic Acids Research, 45*(D1), D877-D887. doi:10.1093/nar/gkw1012

Rappoport, N., Linial, N., & Linial, M. (2013). ProtoNet: charting the expanding universe of protein sequences. *Nature Biotechnology, 31*, 290. doi:10.1038/nbt.2553

Rau, A., Flister, M., Rui, H., & Auer, P. L. (2019). Exploring drivers of gene expression in the Cancer Genome Atlas. *Bioinformatics, 35*(1), 62-68. doi:10.1093/bioinformatics/bty551

Rauscher, B., Heigwer, F., Breinig, M., Winter, J., & Boutros, M. (2017). GenomeCRISPR - a database for high-throughput CRISPR/Cas9 screens. *Nucleic Acids Research, 45*(D1), D679-D686. doi:10.1093/nar/gkw997

Rawlings, N. D., Barrett, A. J., Thomas, P. D., Huang, X., Bateman, A., & Finn, R. D. (2018). The MEROPS database of proteolytic enzymes, their substrates and inhibitors in 2017 and a comparison with peptidases in the PANTHER database. *Nucleic Acids Research, 46*(D1), D624-D632. doi:10.1093/nar/gkx1134

Rech de Laval, V., Deleage, G., Aouacheria, A., & Combet, C. (2014). BCL2DB: database of BCL-2 family members and BH3-only proteins. *Database (Oxford), 2014*, bau013. doi:10.1093/database/bau013

Relling, M. V., & Klein, T. E. (2011). CPIC: Clinical Pharmacogenetics Implementation Consortium of the Pharmacogenomics Research Network. *Clin Pharmacol Ther, 89*(3), 464-467. doi:10.1038/clpt.2010.279

Ren, Y., Gong, W., Zhou, H., Wang, Y., Xiao, F., & Li, T. (2009). siRecords: a database of mammalian RNAi experiments and efficacies. *Nucleic Acids Res, 37*(Database issue), D146-149. doi:10.1093/nar/gkn817

Reymond, J.-L. (2015). The Chemical Space Project. *Accounts of Chemical Research, 48*(3), 722-730. doi:10.1021/ar500432k

Richard, A. M., Judson, R. S., Houck, K. A., Grulke, C. M., Volarath, P., Thillainadarajah, I., . . . Thomas, R. S. (2016). ToxCast Chemical Landscape: Paving the Road to 21st Century Toxicology. *Chemical Research in Toxicology, 29*(8), 1225-1251. doi:10.1021/acs.chemrestox.6b00135

Richardson, L. J., Rawlings, N. D., Salazar, G. A., Almeida, A., Haft, D. R., Ducq, G., . . . Finn, R. D. (2018). Genome properties in 2019: a new companion database to InterPro for the inference of complete functional attributes. *Nucleic Acids Research*, gky1013-gky1013. doi:10.1093/nar/gky1013

RNAcentral: a hub of information for non-coding RNA sequences. (2018). *Nucleic Acids Res*. doi:10.1093/nar/gky1034

Roadmap Epigenomics, C., Kundaje, A., Meuleman, W., Ernst, J., Bilenky, M., Yen, A., . . . Kellis, M. (2015). Integrative analysis of 111 reference human epigenomes. *Nature, 518*, 317. doi:10.1038/nature14248

Roberts, G., Myatt, G. J., Johnson, W. P., Cross, K. P., & Blower, P. E., Jr. (2000). LeadScope: software for exploring large sets of screening data. *J Chem Inf Comput Sci, 40*(6), 1302-1314.

Robinson, J., Halliwell, J. A., McWilliam, H., Lopez, R., & Marsh, S. G. E. (2013). IPD—the Immuno Polymorphism Database. *Nucleic Acids Research, 41*(D1), D1234-D1240. doi:10.1093/nar/gks1140

Roider, H. G., Pavlova, N., Kirov, I., Slavov, S., Slavov, T., Uzunov, Z., & Weiss, B. (2014). Drug2Gene: an exhaustive resource to explore effectively the drug-target relation network. *BMC Bioinformatics, 15*(1), 68. doi:10.1186/1471-2105-15-68

Romagné, F., Santesmasses, D., White, L., Sarangi, G. K., Mariotti, M., Hübler, R., . . . Castellano, S. (2014). SelenoDB 2.0: annotation of selenoprotein genes in animals and their genetic diversity in humans. *Nucleic Acids Research, 42*(D1), D437-D443. doi:10.1093/nar/gkt1045

Rosenthal, A., Gabrielian, A., Engle, E., Hurt, D. E., Alexandru, S., Crudu, V., . . . Tartakovsky, M. (2017). The TB Portals: an Open-Access, Web-Based Platform for Global Drug-Resistant-Tuberculosis Data Sharing and Analysis. *55*(11), 3267-3282. doi:10.1128/JCM.01013-17 %J Journal of Clinical Microbiology

Rosikiewicz, W., Suzuki, Y., & Makałowska, I. (2018). OverGeneDB: a database of 5′ end protein coding overlapping genes in human and mouse genomes. *Nucleic Acids Research, 46*(D1), D186-D193. doi:10.1093/nar/gkx948

Ross, J. S., Ritchie, J. D., Finn, E., Desai, N. R., Lehman, R. L., Krumholz, H. M., & Gross, C. P. (2016). Data sharing through an NIH central database repository: a cross-sectional survey of BioLINCC users. *BMJ Open, 6*(9), e012769. doi:10.1136/bmjopen-2016-012769

Roth, B. L., Lopez, E., Patel, S., & Kroeze, W. K. (2000). The Multiplicity of Serotonin Receptors: Uselessly Diverse Molecules or an Embarrassment of Riches? *The Neuroscientist, 6*(4), 252-262. doi:10.1177/107385840000600408

Rual, J.-F., Venkatesan, K., Hao, T., Hirozane-Kishikawa, T., Dricot, A., Li, N., . . . Vidal, M. (2005). Towards a proteome-scale map of the human protein–protein interaction network. *Nature, 437*, 1173. doi:10.1038/nature04209

Ruddigkeit, L., van Deursen, R., Blum, L. C., & Reymond, J.-L. (2012). Enumeration of 166 Billion Organic Small Molecules in the Chemical Universe Database GDB-17. *Journal of Chemical Information and Modeling, 52*(11), 2864-2875. doi:10.1021/ci300415d

Ruepp, A., Waegele, B., Lechner, M., Brauner, B., Dunger-Kaltenbach, I., Fobo, G., . . . Mewes, H. W. (2010). CORUM: the comprehensive resource of mammalian protein complexes--2009. *Nucleic Acids Res, 38*(Database issue), D497-501. doi:10.1093/nar/gkp914

Rusinova, I., Forster, S., Yu, S., Kannan, A., Masse, M., Cumming, H., . . . Hertzog, P. J. (2013). Interferome v2.0: an updated database of annotated interferon-regulated genes. *Nucleic Acids Research, 41*(Database issue), D1040-D1046. doi:10.1093/nar/gks1215

Russo, F., Di Bella, S., Vannini, F., Berti, G., Scoyni, F., Cook, H. V., . . . Ferro, A. (2018). miRandola 2017: a curated knowledge base of non-invasive biomarkers. *Nucleic Acids Research, 46*(D1), D354-D359. doi:10.1093/nar/gkx854

Rustici, G., Kolesnikov, N., Brandizi, M., Burdett, T., Dylag, M., Emam, I., . . . Sarkans, U. (2013). ArrayExpress update--trends in database growth and links to data analysis tools. *Nucleic Acids Research, 41*(Database issue), D987-D990. doi:10.1093/nar/gks1174

Sachana, M. (2018). *Introduction to Effectopedia: a platform for creating quantitative Adverse Outcome Pathways* (Vol. 295).

Safran, M., Dalah, I., Alexander, J., Rosen, N., Iny Stein, T., Shmoish, M., . . . Lancet, D. (2010). GeneCards Version 3: the human gene integrator. *Database (Oxford), 2010*, baq020-baq020. doi:10.1093/database/baq020

Saier, M. H., Jr., Reddy, V. S., Tsu, B. V., Ahmed, M. S., Li, C., & Moreno-Hagelsieb, G. (2016). The Transporter Classification Database (TCDB): recent advances. *Nucleic Acids Res, 44*(D1), D372-379. doi:10.1093/nar/gkv1103

Saito, T., & Kinugasa, S. (2011). Development and release of a spectral database for organic compounds- Key to the continual services and success of a large-scale database. *Synthesiology, 4*(1), 26-35. doi:10.5571/synth.4.26

Salunke, S., & Tuleu, C. (2015). The STEP database through the end-users eyes—USABILITY STUDY. *International Journal of Pharmaceutics, 492*(1), 316-331. doi:<https://doi.org/10.1016/j.ijpharm.2015.06.016>

Salwinski, L., Miller, C. S., Smith, A. J., Pettit, F. K., Bowie, J. U., & Eisenberg, D. (2004). The Database of Interacting Proteins: 2004 update. *Nucleic Acids Res, 32*(Database issue), D449-451. doi:10.1093/nar/gkh086

Sam, S. A., Teel, J., Tegge, A. N., Bharadwaj, A., & Murali, T. M. (2017). XTalkDB: a database of signaling pathway crosstalk. *Nucleic Acids Research, 45*(D1), D432-D439. doi:10.1093/nar/gkw1037

Samur, M. K., Yan, Z., Wang, X., Cao, Q., Munshi, N. C., Li, C., & Shah, P. K. (2013). canEvolve: A Web Portal for Integrative Oncogenomics. *PloS one, 8*(2), e56228. doi:10.1371/journal.pone.0056228

Sandgren, A., Strong, M., Muthukrishnan, P., Weiner, B. K., Church, G. M., & Murray, M. B. (2009). Tuberculosis Drug Resistance Mutation Database. *PLOS Medicine, 6*(2), e1000002. doi:10.1371/journal.pmed.1000002

Sanz, F., Pognan, F., Steger-Hartmann, T., Díaz, C., eTox, Cases, M., . . . Zamora, I. (2017). Legacy data sharing to improve drug safety assessment: the eTOX project. *Nature Reviews Drug Discovery, 16*, 811. doi:10.1038/nrd.2017.177

Sarkans, U., Gostev, M., Athar, A., Behrangi, E., Melnichuk, O., Ali, A., . . . McEntyre, J. (2018). The BioStudies database-one stop shop for all data supporting a life sciences study. *Nucleic Acids Research, 46*(D1), D1266-D1270. doi:10.1093/nar/gkx965

Sato, T., Yuki, H., Ogura, K., & Honma, T. (2018). *Construction of an integrated database for hERG blocking small molecules* (Vol. 13).

Schaefer, C. F., Anthony, K., Krupa, S., Buchoff, J., Day, M., Hannay, T., & Buetow, K. H. (2009). PID: the Pathway Interaction Database. *Nucleic Acids Research, 37*(Database issue), D674-D679. doi:10.1093/nar/gkn653

Scheinin, I., Myllykangas, S., Borze, I., Bohling, T., Knuutila, S., & Saharinen, J. (2008). CanGEM: mining gene copy number changes in cancer. *Nucleic Acids Res, 36*(Database issue), D830-835. doi:10.1093/nar/gkm802

Schick, J. A., Seisenberger, C., Beig, J., Bürger, A., Iyer, V., Maier, V., . . . Wurst, W. (2016). CRISPR-Cas9 enables conditional mutagenesis of challenging loci. *Scientific reports, 6*, 32326. doi:10.1038/srep32326

Schluter, A., Real-Chicharro, A., Gabaldon, T., Sanchez-Jimenez, F., & Pujol, A. (2010). PeroxisomeDB 2.0: an integrative view of the global peroxisomal metabolome. *Nucleic Acids Res, 38*(Database issue), D800-805. doi:10.1093/nar/gkp935

Schmidt, C. W. (2014). NTP nonneoplastic lesion atlas: a new tool for toxicologic pathology. *Environ Health Perspect, 122*(3), A76-A79. doi:10.1289/ehp.122-A76

Schmidt, E. E., Pelz, O., Buhlmann, S., Kerr, G., Horn, T., & Boutros, M. (2013). GenomeRNAi: a database for cell-based and in vivo RNAi phenotypes, 2013 update. *Nucleic Acids Res, 41*(Database issue), D1021-1026. doi:10.1093/nar/gks1170

Schmidt, U., Struck, S., Gruening, B., Hossbach, J., Jaeger, I. S., Parol, R., . . . Preissner, R. (2009). SuperToxic: a comprehensive database of toxic compounds. *Nucleic Acids Research, 37*(Database issue), D295-D299. doi:10.1093/nar/gkn850

Schnabl, J., Suter, P., & Sigel, R. K. (2012). MINAS--a database of Metal Ions in Nucleic AcidS. *Nucleic Acids Res, 40*(Database issue), D434-438. doi:10.1093/nar/gkr920

Schofield, P. N., Bard, J. B. L., Booth, C., Boniver, J., Covelli, V., Delvenne, P., . . . Ward, A. (2004). Pathbase: a database of mutant mouse pathology. *Nucleic Acids Research, 32*(Database issue), D512-D515. doi:10.1093/nar/gkh124

Scholl, J. H. G., van Hunsel, F. P. A. M., Hak, E., & van Puijenbroek, E. P. (2018). A prediction model-based algorithm for computer-assisted database screening of adverse drug reactions in the Netherlands. *Pharmacoepidemiology and Drug Safety, 27*(2), 199-205. doi:10.1002/pds.4364

Schreyer, A. M., & Blundell, T. L. (2013). CREDO: a structural interactomics database for drug discovery. *Database, 2013*, bat049-bat049. doi:10.1093/database/bat049

Schultheisz, R. J. (1981). TOXLINE: evolution of an online interactive bibliographic database. *J Am Soc Inf Sci, 32*(6), 421-429.

Schwartz, L. M., Woloshin, S., Zheng, E., Tse, T., & Zarin, D. A. (2016). ClinicalTrials.gov and Drugs@FDA: A Comparison of Results Reporting for New Drug Approval Trials. *Ann Intern Med, 165*(6), 421-430. doi:10.7326/m15-2658

Schwenk, J. M., Omenn, G. S., Sun, Z., Campbell, D. S., Baker, M. S., Overall, C. M., . . . Deutsch, E. W. (2017). The Human Plasma Proteome Draft of 2017: Building on the Human Plasma PeptideAtlas from Mass Spectrometry and Complementary Assays. *J Proteome Res, 16*(12), 4299-4310. doi:10.1021/acs.jproteome.7b00467

Sehnal, D., Svobodová Vařeková, R., Pravda, L., Ionescu, C.-M., Geidl, S., Horský, V., . . . Koča, J. (2015). ValidatorDB: database of up-to-date validation results for ligands and non-standard residues from the Protein Data Bank. *Nucleic Acids Research, 43*(D1), D369-D375. doi:10.1093/nar/gku1118

Seiler, C. Y., Park, J. G., Sharma, A., Hunter, P., Surapaneni, P., Sedillo, C., . . . LaBaer, J. (2014). DNASU plasmid and PSI:Biology-Materials repositories: resources to accelerate biological research. *Nucleic Acids Res, 42*(Database issue), D1253-1260. doi:10.1093/nar/gkt1060

Selene, C. H., Chou, J., Holler, J., & T De Rosa, C. (1998). *Minimal Risk Levels (MRLs) for Hazardous Substances* (Vol. 7).

Seltmann, S., Lekschas, F., Müller, R., Stachelscheid, H., Bittner, M.-S., Zhang, W., . . . Kurtz, A. (2016). hPSCreg--the human pluripotent stem cell registry. *Nucleic Acids Research, 44*(D1), D757-D763. doi:10.1093/nar/gkv963

Shameer, K., Glicksberg, B. S., Hodos, R., Johnson, K. W., Badgeley, M. A., Readhead, B., . . . Dudley, J. T. (2018). Systematic analyses of drugs and disease indications in RepurposeDB reveal pharmacological, biological and epidemiological factors influencing drug repositioning. *Brief Bioinform, 19*(4), 656-678. doi:10.1093/bib/bbw136

Shao, W., Pedrioli, P. G. A., Wolski, W., Scurtescu, C., Schmid, E., Vizcaino, J. A., . . . Caron, E. (2018). The SysteMHC Atlas project. *Nucleic Acids Res, 46*(D1), D1237-d1247. doi:10.1093/nar/gkx664

Sharma, A., Deshpande, V., Ghatge, M., & Vangala, R. K. (2017). In-Cardiome: integrated knowledgebase for coronary artery disease enabling translational research. *Database, 2017*, bax077-bax077. doi:10.1093/database/bax077

Sharman, J. L., Benson, H. E., Pawson, A. J., Lukito, V., Mpamhanga, C. P., Bombail, V., . . . Harmar, A. J. (2013). IUPHAR-DB: updated database content and new features. *Nucleic Acids Res, 41*(Database issue), D1083-1088. doi:10.1093/nar/gks960

Shen, J., Cheng, F., Xu, Y., Li, W., & Tang, Y. (2010). Estimation of ADME Properties with Substructure Pattern Recognition. *Journal of Chemical Information and Modeling, 50*(6), 1034-1041. doi:10.1021/ci100104j

Shen, J., Xu, L., Fang, H., Richard, A. M., Bray, J. D., Judson, R. S., . . . Hong, H. (2013). EADB: an estrogenic activity database for assessing potential endocrine activity. *Toxicol Sci, 135*(2), 277-291. doi:10.1093/toxsci/kft164

Shen, Q., Wang, G., Li, S., Liu, X., Lu, S., Chen, Z., . . . Zhang, J. (2016). ASD v3.0: unraveling allosteric regulation with structural mechanisms and biological networks. *Nucleic Acids Research, 44*(D1), D527-D535. doi:10.1093/nar/gkv902

Shi, M. W., Zhang, N. A., Shi, C. P., Liu, C. J., Luo, Z. H., Wang, D. Y., . . . Chen, Z. X. (2018). SAGD: a comprehensive sex-associated gene database from transcriptomes. *Nucleic Acids Res*. doi:10.1093/nar/gky1040

Shiau, C. K., Gu, D. L., Chen, C. F., Lin, C. H., & Jou, Y. S. (2011). IGRhCellID: integrated genomic resources of human cell lines for identification. *Nucleic Acids Res, 39*(Database issue), D520-524. doi:10.1093/nar/gkq1075

Shimoyama, M., De Pons, J., Hayman, G. T., Laulederkind, S. J., Liu, W., Nigam, R., . . . Jacob, H. (2015). The Rat Genome Database 2015: genomic, phenotypic and environmental variations and disease. *Nucleic Acids Res, 43*(Database issue), D743-750. doi:10.1093/nar/gku1026

Shin, G., Kang, T.-W., Yang, S., Baek, S.-J., Jeong, Y.-S., & Kim, S.-Y. (2011). GENT: gene expression database of normal and tumor tissues. *Cancer informatics, 10*, 149-157. doi:10.4137/CIN.S7226

Shin, Y.-C., Shin, S.-Y., So, I., Kwon, D., & Jeon, J.-H. (2011). TRIP Database: a manually curated database of protein-protein interactions for mammalian TRP channels. *Nucleic Acids Research, 39*(Database issue), D356-D361. doi:10.1093/nar/gkq814

Shugay, M., Bagaev, D. V., Zvyagin, I. V., Vroomans, R. M., Crawford, J. C., Dolton, G., . . . Chudakov, D. M. (2018). VDJdb: a curated database of T-cell receptor sequences with known antigen specificity. *Nucleic Acids Research, 46*(D1), D419-D427. doi:10.1093/nar/gkx760

Siddiqui, A. S., Khattra, J., Delaney, A. D., Zhao, Y., Astell, C., Asano, J., . . . Marra, M. A. (2005). A mouse atlas of gene expression: Large-scale digital gene-expression profiles from precisely defined developing C57BL/6J mouse tissues and cells. *102*(51), 18485-18490. doi:10.1073/pnas.0509455102 %J Proceedings of the National Academy of Sciences of the United States of America

Simao, F. A., Waterhouse, R. M., Ioannidis, P., Kriventseva, E. V., & Zdobnov, E. M. (2015). BUSCO: assessing genome assembly and annotation completeness with single-copy orthologs. *Bioinformatics, 31*(19), 3210-3212. doi:10.1093/bioinformatics/btv351

Singleton, J. A., Lloyd, J. C., Mootrey, G. T., Salive, M. E., & Chen, R. T. (1999). An overview of the vaccine adverse event reporting system (VAERS) as a surveillance system. *Vaccine, 17*(22), 2908-2917. doi:<https://doi.org/10.1016/S0264-410X(99)00132-2>

Siramshetty, V. B., Nickel, J., Omieczynski, C., Gohlke, B.-O., Drwal, M. N., & Preissner, R. (2016). WITHDRAWN--a resource for withdrawn and discontinued drugs. *Nucleic Acids Research, 44*(D1), D1080-D1086. doi:10.1093/nar/gkv1192

Sircar, G., Saha, B., Jana, T., Dasgupta, A., Gupta Bhattacharya, S., & Saha, S. (2015). DAAB: a manually curated database of allergy and asthma biomarkers. *Clinical & Experimental Allergy, 45*(7), 1259-1261. doi:doi:10.1111/cea.12569

Skuta, C., Popr, M., Muller, T., Jindrich, J., Kahle, M., Sedlak, D., . . . Bartunek, P. (2017). Probes &amp; Drugs portal: an interactive, open data resource for chemical biology. *Nat Methods, 14*, 759. doi:10.1038/nmeth.4365

Slenter, D. N., Kutmon, M., Hanspers, K., Riutta, A., Windsor, J., Nunes, N., . . . Willighagen, E. L. (2018). WikiPathways: a multifaceted pathway database bridging metabolomics to other omics research. *Nucleic Acids Research, 46*(D1), D661-D667. doi:10.1093/nar/gkx1064

Smelter, A., Astra, M., & Moseley, H. N. B. (2017). A fast and efficient python library for interfacing with the Biological Magnetic Resonance Data Bank. *BMC Bioinformatics, 18*(1), 175. doi:10.1186/s12859-017-1580-5

Smigielski, E. M., Sirotkin, K., Ward, M., & Sherry, S. T. (2000). dbSNP: a database of single nucleotide polymorphisms. *Nucleic Acids Research, 28*(1), 352-355. doi:10.1093/nar/28.1.352

Smirnov, P., Kofia, V., Maru, A., Freeman, M., Ho, C., El-Hachem, N., . . . Haibe-Kains, B. (2018). PharmacoDB: an integrative database for mining in vitro anticancer drug screening studies. *Nucleic Acids Research, 46*(D1), D994-D1002. doi:10.1093/nar/gkx911

Smith, A. C., & Robinson, A. J. (2018). MitoMiner v4.0: an updated database of mitochondrial localization evidence, phenotypes and diseases. *Nucleic Acids Res*. doi:10.1093/nar/gky1072

Smith, B., Ashburner, M., Rosse, C., Bard, J., Bug, W., Ceusters, W., . . . Lewis, S. (2007). The OBO Foundry: coordinated evolution of ontologies to support biomedical data integration. *Nature Biotechnology, 25*, 1251. doi:10.1038/nbt1346

Solecki, R., Heinrich, V., Rauch, M., Chahoud, I., Grote, K., Wölffel, B., . . . Lingk, W. (2010). 12.22 - The DevTox Site: Harmonized Terminology and Database. In C. A. McQueen (Ed.), *Comprehensive Toxicology (Second Edition)* (pp. 339-346). Oxford: Elsevier.

Sone, H., Okura, M., Zaha, H., Fujibuchi, W., Taniguchi, T., Akanuma, H., . . . Yonemoto, J. (2010). Profiles of Chemical Effects on Cells (pCEC): a toxicogenomics database with a toxicoinformatics system for risk evaluation and toxicity prediction of environmental chemicals. *J Toxicol Sci, 35*(1), 115-123.

Spatz, M. A. (2004). Genetics Home Reference. *Journal of the Medical Library Association, 92*(2), 282-283.

Spidlen, J., Breuer, K., Rosenberg, C., Kotecha, N., & Brinkman, R. R. (2012). FlowRepository: A resource of annotated flow cytometry datasets associated with peer-reviewed publications. *Cytometry Part A, 81A*(9), 727-731. doi:doi:10.1002/cyto.a.22106

Spielmann, M., Lupiáñez, D. G., & Mundlos, S. (2018). Structural variation in the 3D genome. *Nature Reviews Genetics, 19*(7), 453-467. doi:10.1038/s41576-018-0007-0

Spjuth, O., Rydberg, P., L. Willighagen, E., Evelo, C., & Jeliazkova, N. (2016). *XMetDB: An open access database for xenobiotic metabolism* (Vol. 8).

Sprague, J., Bayraktaroglu, L., Clements, D., Conlin, T., Fashena, D., Frazer, K., . . . Westerfield, M. (2006). The Zebrafish Information Network: the zebrafish model organism database. *Nucleic Acids Res, 34*(Database issue), D581-585. doi:10.1093/nar/gkj086

Sprenger, J., Lynn Fink, J., Karunaratne, S., Hanson, K., Hamilton, N. A., & Teasdale, R. D. (2008). LOCATE: a mammalian protein subcellular localization database. *Nucleic Acids Res, 36*(Database issue), D230-233. doi:10.1093/nar/gkm950

Sreenivasaiah, P. K., Rani, S., Cayetano, J., Arul, N., & Kim, D. H. (2012). IPAVS: Integrated Pathway Resources, Analysis and Visualization System. *Nucleic Acids Research, 40*(D1), D803-D808. doi:10.1093/nar/gkr1208

Stansfeld, P. J., Goose, J. E., Caffrey, M., Carpenter, E. P., Parker, J. L., Newstead, S., & Sansom, M. S. (2015). MemProtMD: Automated Insertion of Membrane Protein Structures into Explicit Lipid Membranes. *Structure, 23*(7), 1350-1361. doi:10.1016/j.str.2015.05.006

Stergiopoulos, S., Getz, K. A., & Blazynski, C. Evaluating the Completeness of ClinicalTrials.gov. *0*(0), 2168479018782885. doi:10.1177/2168479018782885

Sterling, T., & Irwin, J. J. (2015). ZINC 15 – Ligand Discovery for Everyone. *Journal of Chemical Information and Modeling, 55*(11), 2324-2337. doi:10.1021/acs.jcim.5b00559

Su, M. G., Huang, K. Y., Lu, C. T., Kao, H. J., Chang, Y. H., & Lee, T. Y. (2014). topPTM: a new module of dbPTM for identifying functional post-translational modifications in transmembrane proteins. *Nucleic Acids Res, 42*(Database issue), D537-545. doi:10.1093/nar/gkt1221

Su, W.-H., Chao, C.-C., Yeh, S.-H., Chen, D.-S., Chen, P.-J., & Jou, Y.-S. (2007). OncoDB.HCC: an integrated oncogenomic database of hepatocellular carcinoma revealed aberrant cancer target genes and loci. *Nucleic Acids Research, 35*(suppl_1), D727-D731. doi:10.1093/nar/gkl845

Subramanian, A., Narayan, R., Corsello, S. M., Peck, D. D., Natoli, T. E., Lu, X., . . . Golub, T. R. (2017). A Next Generation Connectivity Map: L1000 Platform And The First 1,000,000 Profiles. *bioRxiv*, 136168. doi:10.1101/136168

Sud, M., Fahy, E., Cotter, D., Azam, K., Vadivelu, I., Burant, C., . . . Subramaniam, S. (2016). Metabolomics Workbench: An international repository for metabolomics data and metadata, metabolite standards, protocols, tutorials and training, and analysis tools. *Nucleic Acids Research, 44*(D1), D463-D470. doi:10.1093/nar/gkv1042

Sud, M., Fahy, E., Cotter, D., Dennis, E. A., & Subramaniam, S. (2012). LIPID MAPS-Nature Lipidomics Gateway: An Online Resource for Students and Educators Interested in Lipids. *J Chem Educ, 89*(2), 291-292. doi:10.1021/ed200088u

Sun, J., Jeliazkova, N., Chupakhin, V., Golib-Dzib, J.-F., Engkvist, O., Carlsson, L., . . . Chen, H. (2017). ExCAPE-DB: an integrated large scale dataset facilitating Big Data analysis in chemogenomics. *Journal of Cheminformatics, 9*(1), 17. doi:10.1186/s13321-017-0203-5

Sun, J., Wu, Y., Xu, H., & Zhao, Z. (2012). DTome: a web-based tool for drug-target interactome construction. *BMC Bioinformatics, 13*(9), S7. doi:10.1186/1471-2105-13-s9-s7

Sun, L. Z., Ji, Z. L., Chen, X., Wang, J. F., & Chen, Y. Z. (2002). ADME-AP: a database of ADME associated proteins. *Bioinformatics, 18*(12), 1699-1700.

Sunkin, S. M., Ng, L., Lau, C., Dolbeare, T., Gilbert, T. L., Thompson, C. L., . . . Dang, C. (2013). Allen Brain Atlas: an integrated spatio-temporal portal for exploring the central nervous system. *Nucleic Acids Res, 41*(Database issue), D996-d1008. doi:10.1093/nar/gks1042

Sushko, I., Novotarskyi, S., Körner, R., Pandey, A. K., Rupp, M., Teetz, W., . . . Tetko, I. V. (2011). Online chemical modeling environment (OCHEM): web platform for data storage, model development and publishing of chemical information. *Journal of Computer-Aided Molecular Design, 25*(6), 533-554. doi:10.1007/s10822-011-9440-2

Suzuki, A., Kawano, S., Mitsuyama, T., Suyama, M., Kanai, Y., Shirahige, K., . . . Suzuki, Y. (2018). DBTSS/DBKERO for integrated analysis of transcriptional regulation. *Nucleic Acids Res, 46*(D1), D229-d238. doi:10.1093/nar/gkx1001

Swainston, N., Baici, A., Bakker, B. M., Cornish-Bowden, A., Fitzpatrick, P. F., Halling, P., . . . Kettner, C. (2018). STRENDA DB: enabling the validation and sharing of enzyme kinetics data. *The FEBS Journal, 285*(12), 2193-2204. doi:doi:10.1111/febs.14427

Szklarczyk, D., Morris, J. H., Cook, H., Kuhn, M., Wyder, S., Simonovic, M., . . . von Mering, C. (2017). The STRING database in 2017: quality-controlled protein-protein association networks, made broadly accessible. *Nucleic Acids Res, 45*(D1), D362-d368. doi:10.1093/nar/gkw937

Takeda, J.-i., Yamasaki, C., Murakami, K., Nagai, Y., Sera, M., Hara, Y., . . . Imanishi, T. (2013). H-InvDB in 2013: an omics study platform for human functional gene and transcript discovery. *Nucleic Acids Research, 41*(D1), D915-D919. doi:10.1093/nar/gks1245

Takemoto, K., & Aie, K. J. B. B. (2017). Limitations of a metabolic network-based reverse ecology method for inferring host–pathogen interactions. *18*(1), 278. doi:10.1186/s12859-017-1696-7

Tan, Z. W., Tee, W. V., Guarnera, E., Booth, L., & Berezovsky, I. N. (2018). AlloMAPS: allosteric mutation analysis and polymorphism of signaling database. *Nucleic Acids Res*. doi:10.1093/nar/gky1028

Tanoli, Z., Alam, Z., Vähä-Koskela, M., Ravikumar, B., Malyutina, A., Jaiswal, A., . . . Aittokallio, T. (2018). Drug Target Commons 2.0: a community platform for systematic analysis of drug–target interaction profiles. *Database, 2018*, bay083-bay083. doi:10.1093/database/bay083

Temple, G., Gerhard, D. S., Rasooly, R., Feingold, E. A., Good, P. J., Robinson, C., . . . Wiemann, S. (2009). The completion of the Mammalian Gene Collection (MGC). *Genome Res, 19*(12), 2324-2333. doi:10.1101/gr.095976.109

Theodoropoulou, M. C., Bagos, P. G., Spyropoulos, I. C., & Hamodrakas, S. J. (2008). gpDB: a database of GPCRs, G-proteins, effectors and their interactions. *Bioinformatics, 24*(12), 1471-1472. doi:10.1093/bioinformatics/btn206

Thorn, C. F., Klein, T. E., & Altman, R. B. (2013). PharmGKB: the Pharmacogenomics Knowledge Base. *Methods Mol Biol, 1015*, 311-320. doi:10.1007/978-1-62703-435-7_20

Tiemeyer, M., Aoki, K., Paulson, J., Cummings, R. D., York, W. S., Karlsson, N. G., . . . Aoki-Kinoshita, K. F. (2017). GlyTouCan: an accessible glycan structure repository. *Glycobiology, 27*(10), 915-919. doi:10.1093/glycob/cwx066

Tohsato, Y., Ho, K. H. L., Kyoda, K., & Onami, S. (2016). SSBD: a database of quantitative data of spatiotemporal dynamics of biological phenomena. *Bioinformatics, 32*(22), 3471-3479. doi:10.1093/bioinformatics/btw417

Tomasulo, P. (2002). ChemIDplus-super source for chemical and drug information. *Med Ref Serv Q, 21*(1), 53-59. doi:10.1300/j115v21n01_04

Toseland, C. P., Clayton, D. J., McSparron, H., Hemsley, S. L., Blythe, M. J., Paine, K., . . . Flower, D. R. (2005). AntiJen: a quantitative immunology database integrating functional, thermodynamic, kinetic, biophysical, and cellular data. *Immunome research, 1*(1), 4-4. doi:10.1186/1745-7580-1-4

Toseland, C. P., McSparron, H., Davies, M. N., & Flower, D. R. (2006). PPD v1.0--an integrated, web-accessible database of experimentally determined protein pKa values. *Nucleic Acids Res, 34*(Database issue), D199-203. doi:10.1093/nar/gkj035

Trupp, M., Altman, T., Fulcher, C. A., Caspi, R., Krummenacker, M., Paley, S., & Karp, P. D. (2010). Beyond the genome (BTG) is a (PGDB) pathway genome database: HumanCyc. *Genome Biology, 11*(Suppl 1), O12-O12. doi:10.1186/gb-2010-11-s1-o12

Tryka, K. A., Hao, L., Sturcke, A., Jin, Y., Wang, Z. Y., Ziyabari, L., . . . Feolo, M. (2014). NCBI’s Database of Genotypes and Phenotypes: dbGaP. *Nucleic Acids Research, 42*(D1), D975-D979. doi:10.1093/nar/gkt1211

Tseng, Y.-T., Li, W., Chen, C.-H., Zhang, S., Chen, J. J., Zhou, X. J., & Liu, C.-C. (2015). IIIDB: a database for isoform-isoform interactions and isoform network modules. *BMC Genomics, 16*(2), S10. doi:10.1186/1471-2164-16-s2-s10

Tsherniak, A., Vazquez, F., Montgomery, P. G., Weir, B. A., Kryukov, G., Cowley, G. S., . . . Hahn, W. C. (2017). Defining a Cancer Dependency Map. *Cell, 170*(3), 564-576.e516. doi:<https://doi.org/10.1016/j.cell.2017.06.010>

Türei, D., Papp, D., Fazekas, D., Földvári-Nagy, L., Módos, D., Lenti, K., . . . Korcsmáros, T. (2013). NRF2-ome: an integrated web resource to discover protein interaction and regulatory networks of NRF2. *Oxidative medicine and cellular longevity, 2013*, 737591-737591. doi:10.1155/2013/737591

Turnbull, C., Scott, R. H., Thomas, E., Jones, L., Murugaesu, N., Pretty, F. B., . . . Caulfield, M. J. (2018). The 100 000 Genomes Project: bringing whole genome sequencing to the NHS. *BMJ, 361*, k1687. doi:10.1136/bmj.k1687

Turner, B., Razick, S., Turinsky, A. L., Vlasblom, J., Crowdy, E. K., Cho, E., . . . Wodak, S. J. (2010). iRefWeb: interactive analysis of consolidated protein interaction data and their supporting evidence. *Database (Oxford), 2010*, baq023-baq023. doi:10.1093/database/baq023

Tyagi, A., Tuknait, A., Anand, P., Gupta, S., Sharma, M., Mathur, D., . . . Raghava, G. P. (2015). CancerPPD: a database of anticancer peptides and proteins. *Nucleic Acids Res, 43*(Database issue), D837-843. doi:10.1093/nar/gku892

Uhlen, M., Oksvold, P., Fagerberg, L., Lundberg, E., Jonasson, K., Forsberg, M., . . . Ponten, F. (2010). Towards a knowledge-based Human Protein Atlas. *Nature Biotechnology, 28*, 1248. doi:10.1038/nbt1210-1248

UniProt, C. (2014). Activities at the Universal Protein Resource (UniProt). *Nucleic Acids Research, 42*(Database issue), D191-D198. doi:10.1093/nar/gkt1140

Ursu, O., Holmes, J., Knockel, J., Bologa, C., Yang, J., Mathias, S., . . . Oprea, T. (2016). *DrugCentral: Online drug compendium* (Vol. 45).

Usmani, S. S., Bedi, G., Samuel, J. S., Singh, S., Kalra, S., Kumar, P., . . . Raghava, G. P. S. (2017). THPdb: Database of FDA-approved peptide and protein therapeutics. *PloS one, 12*(7), e0181748. doi:10.1371/journal.pone.0181748

Vafaee, F., Krycer, J. R., Ma, X., Burykin, T., James, D. E., & Kuncic, Z. (2016). ORTI: An Open-Access Repository of Transcriptional Interactions for Interrogating Mammalian Gene Expression Data. *PloS one, 11*(10), e0164535. doi:10.1371/journal.pone.0164535

Verbruggen, B., Gunnarsson, L., Kristiansson, E., Osterlund, T., Owen, S. F., Snape, J. R., & Tyler, C. R. (2018). ECOdrug: a database connecting drugs and conservation of their targets across species. *Nucleic Acids Res, 46*(D1), D930-d936. doi:10.1093/nar/gkx1024

Veres, D. V., Gyurkó, D. M., Thaler, B., Szalay, K. Z., Fazekas, D., Korcsmáros, T., & Csermely, P. (2015). ComPPI: a cellular compartment-specific database for protein–protein interaction network analysis. *Nucleic Acids Research, 43*(D1), D485-D493. doi:10.1093/nar/gku1007

Vihinen, M. (2014). Variation ontology: annotator guide. *J Biomed Semantics, 5*(1), 9. doi:10.1186/2041-1480-5-9

Visini, R., Awale, M., & Reymond, J.-L. (2017). Fragment Database FDB-17. *Journal of Chemical Information and Modeling, 57*(4), 700-709. doi:10.1021/acs.jcim.7b00020

Vivelo, C. A., Wat, R., Agrawal, C., Tee, H. Y., & Leung, A. K. L. (2017). ADPriboDB: The database of ADP-ribosylated proteins. *Nucleic Acids Research, 45*(D1), D204-D209. doi:10.1093/nar/gkw706

Vizcaino, J. A., Csordas, A., Del-Toro, N., Dianes, J. A., Griss, J., Lavidas, I., . . . Hermjakob, H. (2016). 2016 update of the PRIDE database and its related tools. *Nucleic Acids Res, 44*(22), 11033. doi:10.1093/nar/gkw880

von Eichborn, J., Dunkel, M., Gohlke, B. O., Preissner, S. C., Hoffmann, M. F., Bauer, J. M. J., . . . Preissner, R. (2013). SynSysNet: integration of experimental data on synaptic protein-protein interactions with drug-target relations. *Nucleic Acids Research, 41*(Database issue), D834-D840. doi:10.1093/nar/gks1040

von Eichborn, J., Murgueitio, M. S., Dunkel, M., Koerner, S., Bourne, P. E., & Preissner, R. (2011). PROMISCUOUS: a database for network-based drug-repositioning. *Nucleic Acids Research, 39*(Database issue), D1060-D1066. doi:10.1093/nar/gkq1037

Waghu, F. H., Barai, R. S., Gurung, P., & Idicula-Thomas, S. (2016). CAMPR3: a database on sequences, structures and signatures of antimicrobial peptides. *Nucleic Acids Res, 44*(D1), D1094-1097. doi:10.1093/nar/gkv1051

Wallach, I., & Lilien, R. (2009). The protein-small-molecule database, a non-redundant structural resource for the analysis of protein-ligand binding. *Bioinformatics, 25*(5), 615-620. doi:10.1093/bioinformatics/btp035

Wan, Q., Dingerdissen, H., Fan, Y., Gulzar, N., Pan, Y., Wu, T. J., . . . Mazumder, R. (2015). BioXpress: an integrated RNA-seq-derived gene expression database for pan-cancer analysis. *Database (Oxford), 2015*. doi:10.1093/database/bav019

Wang, C., Hu, G., Wang, K., Brylinski, M., Xie, L., & Kurgan, L. (2016). PDID: database of molecular-level putative protein–drug interactions in the structural human proteome. *Bioinformatics, 32*(4), 579-586. doi:10.1093/bioinformatics/btv597

Wang, G., Li, X., & Wang, Z. (2016). APD3: the antimicrobial peptide database as a tool for research and education. *Nucleic Acids Res, 44*(D1), D1087-1093. doi:10.1093/nar/gkv1278

Wang, G., Luo, X., Wang, J., Wan, J., Xia, S., Zhu, H., . . . Wang, Y. (2018). MeDReaders: a database for transcription factors that bind to methylated DNA. *Nucleic Acids Research, 46*(D1), D146-D151. doi:10.1093/nar/gkx1096

Wang, X., Spandidos, A., Wang, H., & Seed, B. (2012). PrimerBank: a PCR primer database for quantitative gene expression analysis, 2012 update. *Nucleic Acids Research, 40*(D1), D1144-D1149. doi:10.1093/nar/gkr1013

Wanichthanarak, K., Cvijovic, M., Molt, A., & Petranovic, D. (2013). yApoptosis: yeast apoptosis database. *Database (Oxford), 2013*, bat068. doi:10.1093/database/bat068

Weatherspoon, D., & Chattopadhyay, A. (2013). International Classification of Diseases Codes and their Use in Dentistry. *Journal of dental, oral and craniofacial epidemiology, 1*(4), 20-26.

Whetzel, P. L., Noy, N. F., Shah, N. H., Alexander, P. R., Nyulas, C., Tudorache, T., & Musen, M. A. (2011). BioPortal: enhanced functionality via new Web services from the National Center for Biomedical Ontology to access and use ontologies in software applications. *Nucleic Acids Research, 39*(suppl_2), W541-W545. doi:10.1093/nar/gkr469

Whitmore, L., Miles, A. J., Mavridis, L., Janes, R. W., & Wallace, B. A. (2017). PCDDB: new developments at the Protein Circular Dichroism Data Bank. *Nucleic Acids Research, 45*(D1), D303-D307. doi:10.1093/nar/gkw796

Whyte, J., Woodcock, J., & Wang, J. (2017). Review of the Drug Trials Snapshots Program of the US Food and Drug Administration: Women in Cardiovascular Drug Trials. *JAMA internal medicine, 177*(5), 724-727. doi:10.1001/jamainternmed.2017.0033

Wicker, J., Lorsbach, T., Gutlein, M., Schmid, E., Latino, D., Kramer, S., & Fenner, K. (2016). enviPath--The environmental contaminant biotransformation pathway resource. *Nucleic Acids Res, 44*(D1), D502-508. doi:10.1093/nar/gkv1229

Wiederkehr, C., Basavaraj, R., Sarrauste de Menthiere, C., Hermida, L., Koch, R., Schlecht, U., . . . Primig, M. (2004). GermOnline, a cross-species community knowledgebase on germ cell differentiation. *Nucleic Acids Res, 32*(Database issue), D560-567. doi:10.1093/nar/gkh055

Wilks, C., Cline, M. S., Weiler, E., Diehkans, M., Craft, B., Martin, C., . . . Maltbie, D. (2014). The Cancer Genomics Hub (CGHub): overcoming cancer through the power of torrential data. *Database (Oxford), 2014*. doi:10.1093/database/bau093

Williams-DeVane, C. R., Wolf, M. A., & Richard, A. M. (2009). DSSTox chemical-index files for exposure-related experiments in ArrayExpress and Gene Expression Omnibus: enabling toxico-chemogenomics data linkages. *Bioinformatics, 25*(5), 692-694. doi:10.1093/bioinformatics/btp042

Williams, A. J., Grulke, C. M., Edwards, J., McEachran, A. D., Mansouri, K., Baker, N. C., . . . Richard, A. M. (2017). The CompTox Chemistry Dashboard: a community data resource for environmental chemistry. *Journal of Cheminformatics, 9*(1), 61. doi:10.1186/s13321-017-0247-6

Williams, A. J., Harland, L., Groth, P., Pettifer, S., Chichester, C., Willighagen, E. L., . . . Mons, B. (2012). Open PHACTS: semantic interoperability for drug discovery. *Drug Discovery Today, 17*(21), 1188-1198. doi:<https://doi.org/10.1016/j.drudis.2012.05.016>

Williams, E., Moore, J., Li, S. W., Rustici, G., Tarkowska, A., Chessel, A., . . . Swedlow, J. R. (2017). Image Data Resource: a bioimage data integration and publication platform. *Nat Methods, 14*, 775. doi:10.1038/nmeth.4326

Williams, F. (2004). *EDETOX. Evaluations and Predictions of Dermal Absorption of Toxic Chemicals* (Vol. 77).

Winslow, R., Saltz, J., Foster, I., Carr, J., Ge, Y., I. Miller, M., . . . Shipway, M. (2011). *The Cardiovascular Research (CVRG) Grid*.

Winter, C., Henschel, A., Kim, W. K., & Schroeder, M. (2006). SCOPPI: a structural classification of protein-protein interfaces. *Nucleic Acids Res, 34*(Database issue), D310-314. doi:10.1093/nar/gkj099

Wirth, M., Zoete, V., Michielin, O., & Sauer, W. (2012). *SwissBioisostere: A database of molecular replacements for ligand design* (Vol. 41).

Wishart, D., Arndt, D., Pon, A., Sajed, T., Guo, A. C., Djoumbou, Y., . . . Rappaport, S. M. (2015). T3DB: the toxic exposome database. *Nucleic Acids Res, 43*(Database issue), D928-934. doi:10.1093/nar/gku1004

Wishart, D. S., Arndt, D., Berjanskii, M., Guo, A. C., Shi, Y., Shrivastava, S., . . . Lin, G. (2008). PPT-DB: the protein property prediction and testing database. *Nucleic Acids Research, 36*(suppl_1), D222-D229. doi:10.1093/nar/gkm800

Wishart, D. S., Feunang, Y. D., Marcu, A., Guo, A. C., Liang, K., Vazquez-Fresno, R., . . . Scalbert, A. (2018). HMDB 4.0: the human metabolome database for 2018. *Nucleic Acids Res, 46*(D1), D608-d617. doi:10.1093/nar/gkx1089

Wishart, D. S., Knox, C., Guo, A. C., Cheng, D., Shrivastava, S., Tzur, D., . . . Hassanali, M. (2008). DrugBank: a knowledgebase for drugs, drug actions and drug targets. *Nucleic Acids Research, 36*(Database issue), D901-D906. doi:10.1093/nar/gkm958

Wiwatwattana, N., & Kumar, A. (2005). Organelle DB: a cross-species database of protein localization and function. *Nucleic Acids Research, 33*(Database issue), D598-604. doi:10.1093/nar/gki071

Wood, E. H. (2004). The National Comprehensive Cancer Network (NCCN). *Journal of the Medical Library Association, 92*(3), 382-383.

Wu, C. H., Huang, H., Nikolskaya, A., Hu, Z., & Barker, W. C. (2004). The iProClass integrated database for protein functional analysis. *Comput Biol Chem, 28*(1), 87-96.

Wullenweber, A., Kroner, O., Kohrman, M., Maier, A., Dourson, M., Rak, A., . . . Tomljanovic, C. (2008). Resources for global risk assessment: The International Toxicity Estimates for Risk (ITER) and Risk Information Exchange (RiskIE) databases. *Toxicology and Applied Pharmacology, 233*(1), 45-53. doi:<https://doi.org/10.1016/j.taap.2007.12.035>

Xie, H. (2017). Validated Antibody Database (VAD): a database of antibodies curated from publications. *31*(1_supplement), 983.983-983.983. doi:10.1096/fasebj.31.1_supplement.983.3

Xu, L.-M., Li, J.-R., Huang, Y., Zhao, M., Tang, X., & Wei, L. (2012). AutismKB: an evidence-based knowledgebase of autism genetics. *Nucleic Acids Research, 40*(D1), D1016-D1022. doi:10.1093/nar/gkr1145

Xu, Q., Liu, K., Lin, X., Qin, Y., Chen, L., Cheng, J., . . . Ji, Z. (2017). *ADMETNet: The knowledge base of pharmacokinetics and toxicology network* (Vol. 44).

Xue, R., Fang, Z., Zhang, M., Yi, Z., Wen, C., & Shi, T. (2013). TCMID: Traditional Chinese Medicine integrative database for herb molecular mechanism analysis. *Nucleic Acids Research, 41*(Database issue), D1089-D1095. doi:10.1093/nar/gks1100

Yang, C., Bienfait, B., Cronin, M. T. D., Fioravanzo, E., Gatnik, M., Kleinöder, T., . . . Tarkhov, A. (2018). *ToxGPS, a solution guiding read-across workflow based on chemoinformatics and safety assessment* (Vol. 295).

Yang, W., Soares, J., Greninger, P., Edelman, E. J., Lightfoot, H., Forbes, S., . . . Garnett, M. J. (2013). Genomics of Drug Sensitivity in Cancer (GDSC): a resource for therapeutic biomarker discovery in cancer cells. *Nucleic Acids Research, 41*(Database issue), D955-D961. doi:10.1093/nar/gks1111

Yaniv, Z., Faruque, J., Howe, S., Dunn, K., Sharlip, D., Bond, A., . . . Yoo, T. S. (2016). The National Library of Medicine Pill Image Recognition Challenge: An Initial Report. *IEEE Applied Imagery Pattern Recognition Workshop : [proceedings]. IEEE Applied Imagery Pattern Recognition Workshop, 2016*, 10.1109/AIPR.2016.8010584. doi:10.1109/AIPR.2016.8010584

Yeung, C. K., Yoshida, K., Kusama, M., Zhang, H., Ragueneau-Majlessi, I., Argon, S., . . . Huang, S. M. (2015). Organ Impairment—Drug–Drug Interaction Database: A Tool for Evaluating the Impact of Renal or Hepatic Impairment and Pharmacologic Inhibition on the Systemic Exposure of Drugs. *CPT: Pharmacometrics & Systems Pharmacology, 4*(8), 489-494. doi:10.1002/psp4.55

Yi, Y., Zhao, Y., Li, C., Zhang, L., Huang, H., Li, Y., . . . Wang, D. (2017). RAID v2.0: an updated resource of RNA-associated interactions across organisms. *Nucleic Acids Res, 45*(D1), D115-d118. doi:10.1093/nar/gkw1052

Yoo, M., Shin, J., Kim, J., Ryall, K. A., Lee, K., Lee, S., . . . Tan, A. C. (2015). DSigDB: drug signatures database for gene set analysis. *Bioinformatics, 31*(18), 3069-3071. doi:10.1093/bioinformatics/btv313

Yoo, M., Shin, J., Kim, J., Ryall, K. A., Lee, K., Lee, S., . . . Tan, A. C. (2015). DSigDB: drug signatures database for gene set analysis. *Bioinformatics, 31*(18), 3069-3071. doi:10.1093/bioinformatics/btv313

Yu, K., Zhang, J., Chen, M., Xu, X., Suzuki, A., Ilic, K., & Tong, W. (2014). Mining hidden knowledge for drug safety assessment: topic modeling of LiverTox as a case study. *BMC Bioinformatics, 15 Suppl 17*, S6. doi:10.1186/1471-2105-15-s17-s6

Yue, M., Zhou, D., Zhi, H., Wang, P., Zhang, Y., Gao, Y., . . . Li, X. (2018). MSDD: a manually curated database of experimentally supported associations among miRNAs, SNPs and human diseases. *Nucleic Acids Research, 46*(D1), D181-D185. doi:10.1093/nar/gkx1035

Zanzi, A., & Wittwehr, C. (2017). Searching Online Chemical Data Repositories via the ChemAgora Portal. *Journal of Chemical Information and Modeling, 57*(12), 2905-2910. doi:10.1021/acs.jcim.7b00086

Zeng, K., Bodenreider, O., Kilbourne, J., & Nelson, S. J. (2006). RxNav: Providing Standard Drug Information. *AMIA Annual Symposium Proceedings, 2006*, 1198-1198.

Zeng, X., Tao, L., Zhang, P., Qin, C., Chen, S., He, W., . . . Chen, Y. Z. (2017). HEROD: a human ethnic and regional specific omics database. *Bioinformatics, 33*(20), 3276-3282. doi:10.1093/bioinformatics/btx340

Zeng, X., Zhang, P., He, W., Qin, C., Chen, S., Tao, L., . . . Chen, Y. Z. (2018). NPASS: natural product activity and species source database for natural product research, discovery and tool development. *Nucleic Acids Res, 46*(D1), D1217-d1222. doi:10.1093/nar/gkx1026

Zerbino, D. R., Achuthan, P., Akanni, W., Amode, M R., Barrell, D., Bhai, J., . . . Flicek, P. (2018). Ensembl 2018. *Nucleic Acids Research, 46*(D1), D754-D761. doi:10.1093/nar/gkx1098

Zhang, C., Tao, L., Qin, C., Zhang, P., Chen, S., Zeng, X., . . . Chen, Y. Z. (2015). CFam: a chemical families database based on iterative selection of functional seeds and seed-directed compound clustering. *Nucleic Acids Research, 43*(Database issue), D558-D565. doi:10.1093/nar/gku1212

Zhang, J., Baran, J., Cros, A., Guberman, J. M., Haider, S., Hsu, J., . . . Kasprzyk, A. (2011). International Cancer Genome Consortium Data Portal--a one-stop shop for cancer genomics data. *Database (Oxford), 2011*, bar026-bar026. doi:10.1093/database/bar026

Zhang, L., Chang, S., Li, Z., Zhang, K., Du, Y., Ott, J., & Wang, J. (2012). ADHDgene: a genetic database for attention deficit hyperactivity disorder. *Nucleic Acids Res, 40*(Database issue), D1003-1009. doi:10.1093/nar/gkr992

Zhang, Q., Yang, B., Chen, X., Xu, J., Mei, C., & Mao, Z. (2014). Renal Gene Expression Database (RGED): a relational database of gene expression profiles in kidney disease. *Database (Oxford), 2014*. doi:10.1093/database/bau092

Zhang, Q. C., Petrey, D., Garzón, J. I., Deng, L., & Honig, B. (2013). PrePPI: a structure-informed database of protein-protein interactions. *Nucleic Acids Research, 41*(Database issue), D828-D833. doi:10.1093/nar/gks1231

Zhang, Y., Li, J., Kong, L., Gao, G., Liu, Q.-R., & Wei, L. (2007). NATsDB: Natural Antisense Transcripts DataBase. *Nucleic Acids Research, 35*(Database issue), D156-D161. doi:10.1093/nar/gkl782

Zhang, Y., Yang, C., Wang, S., Chen, T., Li, M., Wang, X., . . . He, F. (2013). LiverAtlas: a unique integrated knowledge database for systems-level research of liver and hepatic disease. *Liver International, 33*(8), 1239-1248. doi:doi:10.1111/liv.12173

Zhao, M., Sun, J., & Zhao, Z. (2013). TSGene: a web resource for tumor suppressor genes. *Nucleic Acids Res, 41*(Database issue), D970-976. doi:10.1093/nar/gks937

Zhu, Q., Freimuth, R. R., Pathak, J., Durski, M. J., & Chute, C. G. (2013). Disambiguation of PharmGKB drug–disease relations with NDF-RT and SPL. *Journal of Biomedical Informatics, 46*(4), 690-696. doi:<https://doi.org/10.1016/j.jbi.2013.05.005>

Zuo, D., Mohr, S. E., Hu, Y., Taycher, E., Rolfs, A., Kramer, J., . . . LaBaer, J. (2007). PlasmID: a centralized repository for plasmid clone information and distribution. *Nucleic Acids Research, 35*(Database issue), D680-D684. doi:10.1093/nar/gkl898
